# Supplementary material for: Exploration of recovery of people living with severe mental illness (SMI) in low/middle-income countries (LMICs): a scoping review
Source: BMJ Open. 2021 Mar 24;11(3):e045005. doi: 10.1136/bmjopen-2020-045005 (PMC7993175; doi:10.1136/bmjopen-2020-045005)
Supplement: Supplementary data [file bmjopen-2020-045005supp001.pdf]

Open access Protocol

BMJ Open

Exploration of recovery of people living with severe mental illness (SMI) in low-income and middle-income countries (LMIC): a scoping review protocol

Fadia Gamieldien ,[1,2] Roshan Galvaan,[2] Bronwyn Myers,[3,4] Katherine Sorsdahl[1]

To cite: Gamieldien F, Galvaan R, Myers B, *et al*. Exploration of recovery of people living with severe mental illness (SMI) in low-income and middle-income countries (LMIC): a scoping review protocol. *BMJ Open* 2020;**10**:e032912. doi:10.1136/bmjopen-2019-032912

► Prepublication history and additional material for this paper are available online. To view these files, please visit the journal online (http://dx.doi.org/10.1136/bmjopen-2019-032912).

Received 11 July 2019
Revised 24 December 2019
Accepted 06 January 2020

Check for updates

© Author(s) (or their employer(s)) 2020. Re-use permitted under CC BY. Published by BMJ.

[1]Alan J. Flisher Centre for Public Mental Health, Department of Psychiatry and Mental Health, University of Cape Town, Cape Town, South Africa
[2]Division of Occupational Therapy, Department of Health and Rehabilitation Sciences, University of Cape Town, Cape Town, South Africa
[3]Alcohol, Tobacco and Other Drug Research Unit, South African Medical Research Council, Cape Town, South Africa
[4]Division of Addiction Psychiatry, Department of Psychiatry and Mental Health, University of Cape Town, Cape Town, South Africa

Correspondence to
Fadia Gamieldien;
fadia.gamieldien@uct.ac.za

ABSTRACT

Introduction The construct of recovery was conceptualised in high-income countries and its applicability in low-income and middle- income countries is underexplored. A scoping review is proposed to synthesise knowledge, review conceptual overlap and map key elements of recovery from severe mental illness in low-income and middle-income countries. We aim to appraise the literature so as to inform future recovery-oriented services that consider the cultural and contextual influences on recovery from severe mental illness.

Methods and analysis The following electronic databases: MEDLINE via PubMed, SCOPUS (which included contents of Embase), PsycINFO, CINAHL, Africa-Wide Information, PsycARTICLES, Health source: Nursing/Academic Edition, Academic Search Premier and SocINDEX all via the EBSCOHOST platform, the Latin American and Caribbean Health Sciences Literature, the Cochrane Centre Register of Controlled Trials) and grey literature sources will be searched between May and December 2019. Eligible studies will be independently screened for inclusion and exclusion by two reviewers using a checklist developed for this purpose. Studies published between January 1993 and November 2019 that focus on recovery from severe mental illness in a low-income and middle-income country will be included. Findings will be compared and discrepancies will be discussed. Unresolved discrepancies will be referred to a third reviewer. All bibliographic data and study characteristics will be extracted and thematically analysed using a tool developed through an iterative process by the research team. Indicators will be classified according to a predefined conceptual framework and categorised and described using qualitative content analysis.

Ethics and dissemination The review aims to synthesise information from available publications, hence it does not require ethical approval. The results will be disseminated through publications, conference presentations and future workshops with stakeholders involved within the recovery paradigm of mental health policy and practice. The scoping review title is registered with the Joanna Briggs Institute.

Strengths and limitations of this study

- This scoping review allows for the systematic synthesis of knowledge on recovery from severe mental illness in low-income and middle-income countries (LMICs) in a rigorous and methodological manner.
- This review will highlight the diversity of severe mental illness since there is no agreed on criteria for including mental illnesses into this category.
- Knowledge gaps related to recovery as a multidimensional construct in LMICs will be identified.
- Only conceptual and theoretical underpinnings of recovery as a construct in LMIC will be discussed without a focus on intervention outcomes.
- Data synthesis will be limited to full-text articles available in English only and published between January 1993 and November 2019. Grey literature and unpublished studies in Latin America will be affected by the decision to exclude articles not available in English.

INTRODUCTION

Mental, neurological and substance use (MNS) disorders contribute significantly to the global burden of disease. Whiteford *et al*[1] report that MNS disorders account for 10% of disability-adjusted life years (DALYs). However, it has been argued that the true global burden of mental health problems may be underestimated by more than one-third and that DALYs are closer to 13%.[2] The lifetime prevalence of severe mental illness (SMIs) ranges between 1% and 4%.[3] Although this is relatively low in comparison to common mental disorders, SMIs are more disabling and require complex, long-term interventions for the person and their family.[2] Given the move towards the deinstitutionalisation of people with psychiatric disorders, shorter hospital stays are resulting in premature discharge rates and repeated relapse, creating a revolving door phenomenon.[4] These frequent hospital readmissions are costly to the healthcare system.[5]

In low-income and middle-income countries (LMICs), many people living with an SMI are not detected and as a result do not receive the treatment they need.[3 6 7] In a service

BMJ Open: first published as 10.1136/bmjopen-2019-032912 on 3 February 2020. Downloaded from http://bmjopen.bmj.com/ on February 4, 2020 at University of Cape Town Libraries. Protected by copyright.

utilisation review of WHO member countries, it was found that the lifetime prevalence of mental disorders among adults was between 12% and 49%.[6] The review found the treatment gap to be wide across the psychiatric disorders examined. The treatment gap for schizophrenia and non-affective psychosis was 32%. The gap for other disorders was: major depressive disorder, 56%; bipolar disorder, 50%; and alcohol abuse and dependence, 78%.[6] While present in all countries, this treatment gap is greatest in LMICs where there is a need to scale up mental health services.[8]

A systematic review on the scaling up of mental health services in LMICs found that in many countries the majority of resources for mental healthcare are rendered on an inpatient or outpatient basis at large psychiatric hospitals, with little provision of services at primary healthcare facilities or via integration between hospitals and the community.[8] Although there are some nascent programmes to provide services for SMI at primary care facilities, these are generally limited because of a poor integration of mental health into primary healthcare. Additionally, while the lack of coordination between sectors providing mental health services has seen some countries, such as India, have made progress in adopting the District Mental Health Programme, the treatment gap continues in rural areas.[9 10]

Despite the drive for deinstitutionalisation and for the rendering of services at decentralised locations, in many countries most of the mental healthcare budget continues to be allocated to large psychiatric hospitals. In these hospitals, the treatment approach is primarily through medication to alleviate acute clinical symptoms, giving little to no attention to the psychosocial needs of service users.[4 8 11] Unfortunately, the primary focus on clinical recovery and the limited availability of interventions that focus on rehabilitation and personal recovery further exacerbates the revolving door phenomenon and ongoing treatment gap.[4 12]

Recovery-oriented approaches offer an extension to medical models of intervention, as they are not solely focused on symptomatic improvement as a marker of clinical recovery.[13] Personal recovery for people with SMI is conceptually distinct from the present medical definition of recovery, which is clinical in nature and mostly values symptom remission.[14 15] Personal recovery proposes that a person develops new meaning and purpose in their life over the longitudinal course of their illness.[13 16] Psychiatric rehabilitation has highlighted the need to move beyond treating the symptoms of the illness, to treating its functional and disabling consequences as well.[17] Deegan, a mental health professional and person living with schizophrenia, suggested that mental health service users do not 'get rehabilitated' by others, but instead they are active participants in their journey to recover new and valued personal meaning and purpose.[18]

The recovery model was initiated in high-income countries (HICs) with the mental health consumer and survivor movement at a time when having a diagnosis of SMI was highly stigmatised and the prognosis was poor.[18] The idea that people with an SMI cannot recover was challenged by people living with schizophrenia who were leading meaningful lives in their communities after deinstitutionalisation, highlighting that recovery was not dependant on long-term psychiatric treatment.[19 20]For a person living with an SMI, receiving a diagnosis and psychiatric treatment focused on clinical recovery is a necessary step towards recovery, but personal recovery is also critical given the cyclical, non-linear, long-term course of the illness. In Anthony's seminal work,[17] he defined recovery as:

> a deeply personal, unique process of changing ones' attitude, values, feelings, goals, skills and/or roles. It is a way of living a satisfying, hopeful and contributing life even within the limitations caused by illness

Research exploring the meaning of recovery among people living with SMI[21–23] found that recovery means different things to different people, supporting a view that there is no definition of recovery that applies to everyone. A literature review conducted in 2018 explored the meaning of recovery among people living with schizophrenia.[21] This review included 17 studies explaining the subjective meaning of recovery. A majority of the included studies were qualitative, with a few taking a quantitative approach to exploring the meaning of recovery. Of the 17 papers, only four studies were from LMICs (India (n=2), China and South Africa). The results revealed that recovery is complex, non-linear and perceived as both a process and an outcome. The process was viewed as being individual and it was recommended that more consumer perspectives to inform recovery-oriented interventions, which are aligned to individuals' personal goals and aspirations were needed. The complexity embedded in viewing recovery as a process and an outcome with multidimensional indicators has been documented by others.[24–26] Further to this, it was recommended that clinicians, caregivers and researchers explore more qualitative and personal narratives of recovery, the tools needed to measure recovery and the development of community-based recovery- oriented services.[21]

The aforementioned review was limited to recovery in people living with schizophrenia and only included four studies from LMICs. There is current debate around whether people with SMI in LMICs have a better prognosis and a call to re-examine the realities of living with an SMI amidst social, cultural and economic changes in LMICs.[27 28]

Recovery has been viewed as a process or an outcome, with current debates and research seeking to offer conceptual clarity.[12 29] A systematic review conducted by Leamy *et al*[12] led to a proposed conceptual framework for personal recovery in mental illness. The review focused on 97 papers from 13 HICs and identified characteristics, processes and stages of recovery. While the review included individuals from black and minority ethnic groups, it did not include any LMICs.[12] The CHIME

BMJ Open: first published as 10.1136/bmjopen-2019-032912 on 3 February 2020. Downloaded from http://bmjopen.bmj.com/ on February 4, 2020 at University of Cape Town Libraries. Protected by copyright.

framework emerged from this review process. This frameworkconceptualises personal recovery to incorporate the following concepts: **c**onnectedness, **h**ope and **o**ptimism about the future, **i**dentity, **m**eaning in life and **e**mpowerment.[12] This highlights that literature on personal recovery is dominated by research conducted in HICs and that there is a gap in understanding recovery from culturally diverse groups.[30]

Research seeking to understand recovery as a process and an outcome is ongoing.[31] The complexity of the concept and the range of factors impacting recovery has been the topic of recent studies. One such focal area has been on the relationship between homelessness among people living with SMIs and the contribution of stable housing towards recovery.[32 33] Additionally, a recent exploration of metacognition and recovery from mental illness challenged conventional treatment to embrace the notion that recovery is unique, individualised and self-directed and that service users need to be active participants in their recovery processes.[26] A call has been made for mental health service users to be partners in conceptualising recovery as a concept and treatment orientation in order to integrate the notion of recovery into mainstream psychiatry.[31]

A scoping review of systematic reviews and meta-analyses conducted by van Weeghel *et al*[29] mapped the concept of recovery and its assessment. It found that recovery is a personal process that is dynamic and evolves over time.[29] Although 25 studies were included in the review, only one study was conducted by authors from India. Conducting a scoping review focusing specifically on studies conducted in LMICs will contribute to the literature on recovery in these specific contexts and will be useful for the following reasons. First, little attention has been given to describing recovery as a personal and individual journey from LMIC perspectives. As recovery for the person with an SMI takes place in a social context through a range of relationships, more understanding about how contexts can help or hinder recovery is needed.[34]

Second, individual meaning making within recovery is influenced by social, political, economic and human rights factors but further research is needed to understand the diversity of recovery narratives from the perspectives of different population groups.[35] A systematic review and narrative analysis synthesising descriptions and models of personal recovery was undertaken to inform the development of a conceptual framework to guide recovery oriented research and practice.[12] Studies conducted in HIC specifically the USA featured predominantly in the review which included 97 papers. The review culminated in the emergence of a conceptual framework to describe the characteristics of recovery, the processes of recovery and the stages of recovery. The authors reported a difference in studies which included ethnic minorities and where more emphasis was placed on stigma, spirituality, culture and the collectivist nature of recovery. They acknowledged that this area is underexplored.[12]

Third, in many African cultures, an individual's personhood is manifested through their agency in relation to spirituality, society and self.[36] This may impact on the person and how they experience mental illness and recovery. Understanding cultural concepts and the dynamics of interconnectedness means recognising that personhood in LMICs is weaved into how people experience health, illness and recovery.[36] Considering the person as a spiritual and social being provides opportunities to collaborate with caregivers and communities in developing appropriate interventions.[36] In countries that are spiritually diverse, multicultural and home to people of different religious backgrounds, it is important to contextualise mental illness and explore health professionals own perspectives on culture, psychiatry and mental health and where mental illness must be contextualised.[37 38] Given the reasons cited previously, further exploration into the recovery journeys of people living with SMI in LMICs and how they view the dimensions of recovery is warranted.[21]

A scoping review is proposed as a means of synthesising knowledge on a broadly defined topic and systematically mapping key concepts, theories, evidence and research gaps in the topic area, while still being rigorous and methodological in its approach.[39 40] While systematic reviews and meta-analyses limit their parameters to research trials and quantitative data synthesis, scoping reviews adopt a broader approach to the narrative integration of evidence.[41] We aim to appraise the definitions of recovery and its current utilisation for people with SMI in LMICs. The review findings could be used to inform future practice applications, which consider the cultural and contextual factors impacting on recovery[15] from SMI outside of HICs. This scoping review title is registered with the Joanna Briggs Institute (JBI) although JBI and the International Prospective Register of Systematic Reviews (PROSPERO) do not provide registry numbers for scoping reviews at this time.[39]

METHODS

A scoping review was deemed as the most suitable method to map existing research on recovery from SMI in LMICs because it allows for the topic to be located, examined, summarised and be presented rapidly and systematically. Additionally, it could serve as a precursor to a systematic review.[42] The proposed review will be conducted using the Arksey and O'Malley,[40] scoping review methodology and will be reported on using the Preferred Reporting Items for Systematic Reviews and Meta-Analyses guidelines[43] with an understanding that there is much development as scoping reviews gain popularity especially in health-related topics.[39 44]

Arksey and O'Malley[40] offer a methodological framework for conducting a scoping review. Their approach describes six stages: (1) identification of the research question, (2) identification of relevant studies, (3) selection of studies, (4) charting the data, (5) collating, summarising

BMJ Open: first published as 10.1136/bmjopen-2019-032912 on 3 February 2020. Downloaded from http://bmjopen.bmj.com/ on February 4, 2020 at University of Cape Town Libraries. Protected by copyright.

and reporting of results and (6) consultation with stakeholders.[40] The last stage is optional according to Arksey and O'Malley,[40] but others have deemed it a necessary step to aide in methodological rigour.[45] Recommendations to enhance the methodology at each stage will be incorporated into this review.[39 43 45]

Stage 1: identifying the research question

In order to guide the search strategy an iterative process of discussion among authors occurred in order to develop and refine the research question. The question needed to be clear enough to inform the subsequent stages while still reflecting the scope of inquiry.[45] Given that scoping studies are focused on summarising the breadth of evidence on a given topic the authors refined the exploratory question to ask the following:

What is known about recovery from SMIs in LMICs?

This broad question was developed to allow for a comprehensive review of the literature in order to review and appraise the definition of recovery and how it is understood in mental health research and practice in LMICs. While many have written about this in HICs,[23 46] there is a dearth of such information in LMICs .

Stage 2: identifying relevant studies

Given the aim of the study is to identify primary published and unpublished studies which will answer the research question, a comprehensive search strategy was developed to aid this. Two health sciences librarians assisted the first author in the iterative process of developing a search strategy including inclusion and exclusion criteria. Decisions were made about time frames, language, search terms and search strategies. Trial searches were run to assess whether relevant literature could be identified using the proposed strategy. A discussion of the identified databases, search strategy and selection criteria follow.

Databases

Electronic databases will be searched to identify studies published between January 1993 and November 2019. This period was selected as it covers roughly 25 years in the recovery movement. Personal recovery is a construct that has already been defined by Anthony,[17] hence the authors were looking specifically for literature on this construct. Terms used in other disciplines that could also mean recovery were not included as the focus of the search is on recovery as explicitly defined by the recovery movement. Furthermore, recovery knowledge and attitudes among health professionals will be included if reported in the selected studies.

An expert librarian assisted the first author in identifying information sources that are relevant to the research question. The following databases were selected: MEDLINE via PubMed, SCOPUS (which included contents of Embase), PsycINFO, CINAHL, Africa-Wide Information, PsycARTICLES, Health source: Nursing/Academic Edition, Academic Search Premier and SocINDEX all via the EBSCOHOST platform. Additionally, the Latin American and Caribbean Health Sciences Literature, the Cochrane Centre Register of Controlled Trials. Grey literature sources will be identified to expand the search and enhance the data sources. Additional sources will be located by means of handsearching reference lists of relevant papers, contacting study authors, searching trial registers and contacting key personnel involved in recovery-focused programmes in LMICs. In this way, peer-reviewed and grey literature from the biomedical sciences, allied health sciences, social sciences and other disciplinary fields will be included.

Search strategy

Two librarians and the first author developed the eligibility criteria for the scoping review. They identified various definitions of SMIs as well as recovery to include in this search strategy. A list of preliminary search terms and filtering methods was developed. The main filtering methods related to the date range of 25 years (January 1993–November 2019). This period was extended so that new publications would not be missed. The search strategy was refined to include Medical Subject Headings (MeSH terms), filters and Boolean operations to comply with searches across different databases. A search strategy for the PubMed database is included in online supplementary file 1. After the initial search, key words and index terms used across the databases were identified and plotted on a table as indicated below (table 1).

There are no universally accepted guidelines to operationalise the concept of recovery and there is no measure of a gold standard of recovery.[47] The CHIME framework is one proposed guide for the development of recovery measures and identifying recovery outcomes for consideration in clinical practice, but this is limited to HICs. In this review, the key concept is personal recovery as defined by the seminal work of Anthony.[17] In the search strategy, the concept of recovery will be explored through the MeSH terms: Psychiatric Rehabilitation; Mental Health Recovery and Recovery of Function as well as through the free-text terms: Recovery OR recover OR psychosocial rehabilitation OR mental health rehabilitation OR psychiatric rehabilitation.

Selection criteria

Articles will be included in the review if they meet the following inclusion and exclusion criteria (table 2).

Stage 3: study selection

The scoping review guideline for identifying and selecting studies informs article selection and data extraction.[40] Two screening levels are proposed: a title and abstract review and a full-text review. For the first level of screening, two authors (FG and KS) will independently screen the title and abstracts of all retrieved citations against a few select inclusion criteria. Any articles that seem relevant by either or both reviewers will be included in the full-text review. Next, these two reviewers will independently screen the full-text articles to assess whether they meet

BMJ Open: first published as 10.1136/bmjopen-2019-032912 on 3 February 2020. Downloaded from http://bmjopen.bmj.com/ on February 4, 2020 at University of Cape Town Libraries. Protected by copyright.

BMJ Publishing Group Limited (BMJ) disclaims all liability and responsibility arising from any reliance placed on this supplemental material which has been supplied by the author(s)

Table 1 Search strategy terms

Keyword	Alternative
Recovery	Recovery OR recover OR psychosocial rehabilitation OR mental health rehabilitation OR psychiatric rehabilitation OR mental health recovery OR Recovery of function OR Quality of life
Severe mental illness	Severe mental illness OR bipolar OR delusional disorder OR delusional disorders OR major depressive disorder OR major depressive disorders OR schizophrenia OR manic OR manic-depressive OR paranoid disorder OR paranoid disorders OR psychoses OR psychosis OR psychotic disorder OR psychotic disorders OR schizoaffective disorder OR schizoaffective disorders OR Schizophreniform OR serious mental disorder OR serious mental disorders Schizophrenia Spectrum and Other Psychotic Disorders<br>Bipolar and Related Disorders<br>Depressive Disorder
Low-income and middle- income countries	Deprived Countries OR Deprived Population OR Deprived Populations OR Developing Countries OR Developing Country OR Developing Economies OR Developing Economy OR Developing Nation OR Developing Nations OR Developing Population OR Developing Populations OR Developing World OR LAMI Countries OR LAMI Country OR Less Developed Countries OR Less Developed Country OR Less Developed Economies OR Less Developed Nation OR Less Developed Nations OR Less Developed World OR Lesser Developed Countries OR Lesser Developed Nations OR LMIC OR LMICS OR Low GDP OR Low GNP OR Low Gross Domestic OR Low Gross National OR Low Income Countries OR Low Income Country OR Low Income Economies OR Low Income Economy OR Low Income Nations OR Low Income Population OR Low Income Populations OR Lower GDP OR lower gross domestic OR Lower Income Countries OR Lower Income Country OR Lower Income Nations OR Lower Income Population OR Lower Income Populations OR Middle Income Countries OR Middle Income Country OR Middle Income Economies OR Middle Income Nation OR Middle Income Nations OR Middle Income Population OR Middle Income Populations OR Poor Countries OR Poor Country OR Poor Economies OR Poor Economy OR Poor Nation OR Poor Nations OR Poor Population OR Poor Populations OR poor world OR Poorer Countries OR Poorer Economies OR Poorer Economy OR Poorer Nations OR Poorer Population OR Poorer Populations OR Third World OR Transitional Countries OR Transitional Country OR Transitional Economies OR Transitional Economy OR Under Developed Countries OR Under Developed Country OR under developed nations OR Under Developed World OR Under Served Population OR Under Served Populations OR Underdeveloped Countries OR Underdeveloped Country OR underdeveloped economies OR underdeveloped nations OR underdeveloped population OR Underdeveloped World OR Underserved Countries OR Underserved Nations OR Underserved Population OR Underserved Populations<br>OR<br>Afghanistan OR Albania OR Algeria OR American Samoa OR Angola OR Armenia OR Azerbaijan OR Bangladesh OR Belarus OR Byelarus OR Belorussia OR Belize OR Benin OR Bhutan OR Bolivia OR Bosnia OR Botswana OR Brazil OR Bulgaria OR Burma OR Burkina Faso OR Burundi OR Cabo Verde OR Cape Verde OR Cambodia OR Cameroon OR Central African Republic OR Chad OR China OR Colombia OR Comoros OR Comores OR Comoro OR Congo OR Costa Rica OR Côte d'Ivoire OR Cuba OR Djibouti OR Dominica OR Dominican Republic OR Ecuador OR Egypt OR El Salvador OR Equatorial Guinea OR Eritrea OR Ethiopia OR Fiji OR Gabon OR Gambia OR Gaza OR Georgia OR Georgia Republic OR Ghana OR Grenada OR Grenadines OR Guatemala OR Guinea OR Guinea- Bissau OR Guyana OR Haiti OR Herzegovina OR Hercegovina OR Honduras OR India OR Indonesia OR Iran OR Iraq OR Jamaica OR Jordan OR Kazakhstan OR Kenya OR Kiribati OR Democratic People's Republic of Korea OR Kosovo OR Kyrgyz OR Kirghizia OR Kirghiz OR Kyrgyzstan OR Lao PDR OR Laos OR Lebanon OR Lesotho OR Liberia OR Libya OR Macedonia OR Madagascar OR Malawi OR Malay OR Malaya OR Malaysia OR Maldives OR Mali OR Marshall Islands OR Mauritania OR Mauritius OR Mexico OR Micronesia OR Moldova OR Mongolia OR Montenegro OR Morocco OR Mozambique OR Myanmar OR Namibia OR Nepal OR Nicaragua OR Niger OR Nigeria OR Pakistan OR Palau OR Panama OR Papua New Guinea OR Paraguay OR Peru OR Philippines OR Principe OR Romania OR Ruanda OR Rwanda OR Samoa OR Sao Tome OR Senegal OR Serbia OR Sierra Leone OR Solomon Islands OR Somalia OR South Africa OR South Sudan OR Sri Lanka OR St Lucia OR St Vincent OR Sudan OR Surinam OR Suriname OR Swaziland OR Syria OR Syrian Arab Republic OR Tajikistan OR Tadzhikistan OR Tajikistan OR Tadzhik OR Tanzania OR Thailand OR Timor OR Togo OR Tonga OR Tunisia OR Turkey OR Turkmen OR Turkmenistan OR Tuvalu OR Uganda OR Ukraine OR Uzbek OR Uzbekistan OR Vanuatu OR Venezuela OR Vietnam OR West Bank OR Yemen OR Zambia OR Zimbabwe

BMJ Open: first published as 10.1136/bmjopen-2019-032912 on 3 February 2020. Downloaded from http://bmjopen.bmj.com/ on February 4, 2020 at University of Cape Town Libraries. Protected by copyright.

Table 2 Inclusion and exclusion criteria

Criteria for inclusion	Criteria for exclusion
► Published between January 1993 and November 2019.<br>► Qualitative and quantitative empirical (ie, primary research) study designs.<br>► Theoretical literature on the construct of recovery.<br>► Studies will be included if they have been conducted in LMICs.<br>► Peer-reviewed scientific literature.<br>► Literature focusing on the population of interest, that is, adults with SMI in LMICs will be included. The diagnosis of SMI includes schizophrenia spectrum disorders, schizoaffective disorders, bipolar affective disorders, major depressive disorder or related psychotic disorders (schizophreniform, delusional disorder, substance induced psychotic disorder or disorders not otherwise specified) as well as those with a comorbid substance use disorder. Studies and reports will be excluded if they do not include the keywords or alternatives as outlined in table 1.<br>► Explicit mention of severe mental illness and recovery in LMICS.	► The review will be limited to full-text articles written in English. However, the authors acknowledge that this practical decision could mean that potentially relevant papers are missed.<br>► Conference abstracts will be excluded although they will be reviewed to see if full reports were published.<br>► Children will be excluded as the age range for inclusion is 18 years and older.<br>► Duplicate articles from the same study will be excluded.<br>► Policy documents will be excluded although they will be reviewed to access research in LMICs that they might have referenced.<br>► Studies that occurred only in high-income countries, without an LMIC focus will be excluded.<br>► Studies must be conducted in LMIC and not in HIC with participants from LMICs.

LMICs, low-income and middle-income countries.

the inclusion and exclusion criteria. At both levels of screening, inter-rater agreement will be assessed by calculating Cohen's kappa. Any disagreement on study selection will be resolved by discussion with a third rater, who will be a senior member of the research team.

Quality appraisal

The Mixed Methods Appraisal Tool V.2018[48] and the Critical Appraisal Skills Programme Qualitative checklist will be used to assess the quality of included studies although this is not required for a scoping review nor will it influence the inclusion of the study.

Using the aforementioned criteria, the number of studies identified and selected for inclusion in the scoping review will be reported on. A narrative description[39] will accompany the search decision flow chart.[43] The flow chart will show the results from the search, the removal of duplicates, study selection, full retrieval numbers, additional searches from reference lists and a final summary of decisions related to the search decision and an explanation of the review decision process.[39]

Stage 4: charting the data

Next, the two reviewers (FG and KS) will independently extract data from the identified articles and chart it.[40] A draft data charting form will be created in the MS Excel programme to facilitate this process.[40] This form will be pretested and an iterative process of refining the form will be undertaken as the review commences to ensure that all relevant information is extracted.[39]

The form will allow the reviewers to confirm study relevance, record study characteristics and extract information relevant to the review question. The following information will be extracted from the articles: (1) study title; (2) author; (3) study population; (4) participant characteristics; (5) research question; (6) study methodology; (7) study description; (8) intervention type (if applicable); (9) intervention duration (if applicable); (10) outcome measures; 11 summary of findings; (12) definitions of key concepts (recovery and SMIs); (13) conceptual links identified; (14) practice implications and (15) recommendations for further development.

While charting the data, it is important to maintain flexibility around including emerging categories and consulting with the team as this occurs. The final version of the charting form along with definitions of included items will be provided as an appendix to the review. This process will facilitate the timeous completion of the review while also maintaining research rigour.

Stage 5: collating, summarising and reporting the results

All results will be collated, summarised and reported on in order to provide a comprehensive and thorough overview of all the reviewed literature. The authors plan to follow an iterative process and not pre-empt or fit findings into what has already been written about recovery in HICs. This will allow new understandings to be uncovered. While the CHIME framework is considered to offer a comprehensive overview of the process of recovery, it has only been used in HICs with predominantly White populations so its applicability with ethnically and culturally diverse groups is unknown.[12 30] The role of non-government organisations and traditional healers in providing services that facilitate recovery for people living with SMIs will be examined if this arises from the scoping review.

BMJ Open: first published as 10.1136/bmjopen-2019-032912 on 3 February 2020. Downloaded from http://bmjopen.bmj.com/ on February 4, 2020 at University of Cape Town Libraries. Protected by copyright.

A three-step approach[45] of analysing the data, reporting results and applying meaning to results will be followed to provide methodological rigour. Collating and summarising results will include a summary of the types of studies conducted (including the quantity, types of study design, populations, setting) as well as a thematic analysis of the results. More specifically, we will code how studies describe the concept of recovery from SMI and how it is defined and understood in LMICs. The framework used to report the findings will be developed iteratively through the examination of results. Additionally, the authors will report on the broader implications of the review in terms of research, policy and practice.

Stage 6: consultation

While Arksey and O'Malley[40] view the consultation stage as an optional step, Levac *et al*[45] views it as a requirement to ensure methodological rigour. During each stage of the review process, we will consult with an existing stakeholder advisory committee. This advisory committee consists of the research team and key stakeholders including mental health service providers and service users from within the public health and non-profit organisation sectors. This committee has been constituted from the onset of the project and some members are part of a pre-existing group for public mental health specialists. The advisory committee will be consulted to get their views on the relevance of the review, guidance for accessing grey literature and their perspectives on the data extraction process and preliminary findings. Including an advisory committee in the review is informed by a pragmatic approach[49] where the research is treated as a human experience with the understanding that this consultation process is likely to enhance the relevance of this review. It will also support collaborative efforts to facilitate consumer participation, public involvement, access to information and transfer of knowledge.[45]

Patient and public involvement

There is no patient involvement in the protocol development aspect of the study. Service users forming part of the advisory committee referred to stage 6 will be consulted on findings and dissemination of results.

Ethics and dissemination

Since the scoping review methodology aims at synthesising information from available publications, this study does not require ethical approval. Reviews of primary research allow for the mapping of evidence in fields where the body of literature has not yet been comprehensively reviewed or where working definitions and concepts are still developing. The findings will be made available in different formats to facilitate its dissemination. An article reporting on the results will be submitted for publication in a peer-reviewed journal. It will also be presented at relevant conferences and stakeholder engagements involved in mental health policy and practice. These stakeholders include mental health service users (MHSUs), academics, policy-makers, researcher and clinicians. Findings will also be shared via newsletters, policy briefs and social media forums.

Twitter Fadia Gamieldien @fadiagam

Contributors This protocol was developed by the intellectual contributions of all the authors. All authors were involved in developing the review question and conceptualising the approach. FG developed and tested the search terms in consultation with a subject librarian. FG and KS developed the data extraction guideline, and this was revised based on feedback from the other authors. RG, KS and BM provided substantial critique and review of the draft protocol.

Funding This work was supported by the DELTAS Africa Initiative (DEL-15-01). The DELTAS Africa Initiative is an independent funding scheme of the African Academy of Sciences (AAS)'s Alliance for Accelerating Excellence in Science in Africa (AESA) and supported by the New Partnership for Africa's Development Planning and Coordinating Agency (NEPAD Agency) with funding from the Wellcome Trust (DEL-15-01) and the UK government. BM is funded through the South African Medical Research Council.

Disclaimer The views expressed in this publication are those of the author(s) and not necessarily those of AAS, NEPAD Agency, WellcomeTrust or the UK government.

Competing interests None declared.

Patient consent for publication Not required.

Provenance and peer review Not commissioned; externally peer reviewed.

Open access This is an open access article distributed in accordance with the Creative Commons Attribution 4.0 Unported (CC BY 4.0) license, which permits others to copy, redistribute, remix, transform and build upon this work for any purpose, provided the original work is properly cited, a link to the licence is given, and indication of whether changes were made. See: https://creativecommons.org/licenses/by/4.0/.

ORCID iD

Fadia Gamieldien http://orcid.org/0000-0003-2820-6484

REFERENCES

1. Whiteford HA, Ferrari AJ, Degenhardt L, *et al*. The global burden of mental, neurological and substance use disorders: an analysis from the global burden of disease study 2010. *PLoS One* 2015;10:e0116820.
2. Vigo D, Thornicroft G, Atun R. Estimating the true global burden of mental illness. *Lancet Psychiatry* 2016;3:171–8.
3. Kessler RC, Berglund PA, Bruce ML, *et al*. The prevalence and correlates of untreated serious mental illness. *Health Serv Res* 2001;36:987.
4. Petersen I, Lund C, Stein DJ. Optimizing mental health services in low-income and middle-income countries. *Curr Opin Psychiatry* 2011;24:318–23.
5. Sfetcu R, Musat S, Haaramo P, *et al*. Overview of post-discharge predictors for psychiatric re-hospitalisations: a systematic review of the literature. *BMC Psychiatry* 2017;17.
6. Kohn R, Saxena S, Levav I, *et al*. The treatment gap in mental health care. *Bull World Health Organ* 2004;82:858–66.
7. Demyttenaere K, Bruffaerts R, Posada-Villa J, *et al*. Prevalence, severity, and unmet need for treatment of mental disorders in the World Health Organization world mental health surveys. *JAMA* 2004;291:2581–90.
8. Eaton J, McCay L, Semrau M, *et al*. Scale up of services for mental health in low-income and middle-income countries. *Lancet* 2011;378:1592–603.
9. Thornicroft G, Cooper S, Bortel TV, *et al*. Capacity building in global mental health research. *Harv Rev Psychiatry* 2012;20:13–24.
10. Kumar A. Mental health services in rural India: challenges and prospects. *Health* 2011;03:757–61.
11. Lund C, Oosthuizen P, Flisher AJ, *et al*. Pathways to inpatient mental health care among people with schizophrenia spectrum disorders in South Africa. *PS* 2010;61:235–40.
12. Leamy M, Bird V, Boutillier CL, *et al*. Conceptual framework for personal recovery in mental health: systematic review and narrative synthesis. *Br J Psychiatry* 2011;199:445–52.
13. Slade M. *Personal recovery and mental illness: a guide for mental health professionals*. Cambridge University Press, 2009.

BMJ Open: first published as 10.1136/bmjopen-2019-032912 on 3 February 2020. Downloaded from http://bmjopen.bmj.com/ on February 4, 2020 at University of Cape Town Libraries. Protected by copyright.

14 Slade M. Mental illness and well-being: the central importance of positive psychology and recovery approaches. *BMC Health Serv Res* 2010;10:26.
15 Myers NL. Culture, stress and recovery from schizophrenia: lessons from the field for global mental health. *Cult Med Psychiatry* 2010;34:500–28.
16 Tew J, Ramon S, Slade M, *et al*. Social factors and recovery from mental health difficulties: a review of the evidence. *Br J Soc Work* 2012;42:443–60.
17 Anthony WA. Recovery from mental illness: the guiding vision of the mental health service system in the 1990s. *Psychiatr Rehabil J* 1993;16:11–23.
18 Deegan PE. Recovery: the lived experience of rehabilitation. *Psychiatr Rehabil J* 1988;11:11–19.
19 Webb L. The recovery model and complex health needs: what health psychology can learn from mental health and substance misuse service provision. *J Health Psychol* 2012;17:731–41.
20 Farkas M. The vision of recovery today: what it is and what it means for services. *World Psychiatry* 2007;6:68.
21 Mathew ST, Nirmala BP, Kommu JVS. Meaning of recovery among persons with schizophrenia: a literature review. *J Psychosoc Rehabil Mental Health* 2018;5:73–81.
22 Kelly M, Lamont S, Brunero S. An occupational perspective of the recovery journey in mental health. *Br J Occup Ther* 2010;73:129–35.
23 Cabassa LJ, Nicasio A, Whitley R. Picturing recovery: a photovoice exploration of recovery dimensions among people with serious mental illness. *Psychiatr Serv* 2013;64:837–42.
24 Aldersey HM, Adeponle AB, Whitley R. Diverse approaches to recovery from severe mental illness. The Palgrave Handbook of sociocultural perspectives on global mental health 2017:109–27.
25 Frost BG, Tirupati S, Johnston S, *et al*. An integrated Recovery-oriented model (IRM) for mental health services: evolution and challenges. *BMC Psychiatry* 2017;17:22.
26 Lysaker PH, Hamm JA, Hasson-Ohayon I, *et al*. Promoting recovery from severe mental illness: implications from research on metacognition and metacognitive reflection and insight therapy. *World J Psychiatry* 2018;8:1–11.
27 Cohen A, Patel V, Thara R, *et al*. Questioning an axiom: better prognosis for schizophrenia in the developing world? *Schizophr Bull* 2008;34:229–44.
28 Patel V, Saxena S, Lund C, *et al*. The Lancet Commission on global mental health and sustainable development. *Lancet* 2018;392:1553–98.
29 van Weeghel J, van Zelst C, Boertien D, *et al*. Conceptualizations, assessments, and implications of personal recovery in mental illness: a scoping review of systematic reviews and meta-analyses. *Psychiatr Rehabil J* 2019;42:169–81.
30 Brijnath B. Applying the CHIME recovery framework in two culturally diverse Australian communities: qualitative results. *Int J Soc Psychiatry* 2015;61:660–7.
31 Leonhardt BL, Huling K, Hamm JA, *et al*. Recovery and serious mental illness: a review of current clinical and research paradigms and future directions. *Expert Rev Neurother* 2017;17:1117–30.
32 Kirst M, Zerger S, Wise Harris D, *et al*. The promise of recovery: narratives of hope among homeless individuals with mental illness participating in a housing first randomised controlled trial in Toronto, Canada: Table 1. *BMJ Open* 2014;4:e004379.
33 Smartt C, Prince M, Frissa S, *et al*. Homelessness and severe mental illness in low- and middle-income countries: scoping review. *BJPsych Open* 2019;5.
34 Schön U-K, Denhov A, Topor A. Social relationships as a decisive factor in recovering from severe mental illness. *Int J Soc Psychiatry* 2009;55:336–47.
35 Llewellyn-Beardsley J, Rennick-Egglestone S, Callard F, *et al*. Characteristics of mental health recovery narratives: systematic review and narrative synthesis. *PLoS One* 2019;14:e0214678.
36 Kpanake L. Cultural concepts of the person and mental health in Africa. *Transcult Psychiatry* 2018;55:198–218.
37 Janse van Rensburg ABR. South African Society of Psychiatrists guidelines for the integration of spirituality in the approach to psychiatric practice. *S Afr J Psychiatr* 2014;20:7–39.
38 Swartz L, Foster D. Images of culture and mental illness : South African psychiatric approaches. *Soc Dyn* 1984;10:17–25.
39 Peters MDJ, Godfrey CM, Khalil H, *et al*. Guidance for conducting systematic scoping reviews. *Int J Evid Based Healthc* 2015;13:141–6.
40 Arksey H, O'Malley L. Scoping studies: towards a methodological framework. *Int J Soc Res Methodol* 2005;8:19–32.
41 Dijkers M. What is a scoping review?. KT Update 2015;4.
42 McKinstry C, Brown T, Gustafsson L. Scoping reviews in occupational therapy: the what, why, and how to. *Aust Occup Ther J* 2014;61:58–66.
43 Tricco AC, Lillie E, Zarin W, *et al*. Prisma extension for scoping reviews (prisma-scr): checklist and explanation. *Ann Intern Med* 2018;169:467–73.
44 Pham MT, Rajić A, Greig JD, *et al*. A scoping review of scoping reviews: advancing the approach and enhancing the consistency. *Res Synth Methods* 2014;5:371–85.
45 Levac D, Colquhoun H, O'Brien KK. Scoping studies: advancing the methodology. *Implement Sci* 2010;5:69.
46 Slade M, Longden E. Empirical evidence about recovery and mental health. *BMC Psychiatry* 2015;15:285.
47 Shanks V, Williams J, Leamy M, *et al*. Measures of personal recovery: a systematic review. *Psychiatr Serv* 2013;64:974–80.
48 Hong QN, Pluye P, Fàbregues S, *et al*. Mixed methods appraisal tool (MMAT), version 2018. *IC Canadian Intellectual Property Office, Industry Canada* 2018.
49 Morgan DL. Pragmatism as a paradigm for social research. *Qual Inq* 2014;20:1045–53.

BMJ Open: first published as 10.1136/bmjopen-2019-032912 on 3 February 2020. Downloaded from http://bmjopen.bmj.com/ on February 4, 2020 at University of Cape Town Libraries. Protected by copyright.
