# Supplementary material for: Exploration of recovery of people living with severe mental illness (SMI) in low/middle-income countries (LMICs): a scoping review
Source: BMJ Open. 2021 Mar 24;11(3):e045005. doi: 10.1136/bmjopen-2020-045005 (PMC7993175; doi:10.1136/bmjopen-2020-045005)
Supplement: Supplementary data [file bmjopen-2020-045005supp002.pdf]

Supplementary file 2

PubMed Search

Population, Concept, Context (PCC)

**Population:**		
#1	MeSH terms:	Schizophrenia Spectrum and Other Psychotic Disorders<br>Bipolar and Related Disorders<br>Depressive disorder, Major
#2	Free text:	Severe mental illness OR bipolar OR delusional disorder OR delusional disorders OR major depressive disorder OR major depressive disorders OR schizophrenia OR manic OR manic-depressive OR paranoid disorder OR paranoid disorders OR psychoses OR psychosis OR psychotic disorder OR psychotic disorders OR schizoaffective disorder OR schizoaffective disorders OR Schizophreniform OR serious mental disorder OR serious mental disorders
#3	#1 OR #2	
**Concept:**		
#4	MeSH terms:	Psychiatric Rehabilitation<br>Mental Health Recovery<br>Recovery of Function
#5	Free text:	Recovery OR recover OR psychosocial rehabilitation OR mental health rehabilitation OR psychiatric rehabilitation
#6	#4 OR #5	
**Context: Low and Middle Income Countries (LMICS)**		
#7	Free text:	Deprived Countries OR Deprived Population OR Deprived Populations OR Developing Countries OR Developing Country OR Developing Economies OR Developing Economy OR Developing Nation OR Developing Nations OR Developing Population OR Developing Populations OR Developing World OR LAMI Countries OR LAMI Country OR Less Developed Countries OR Less Developed Country OR Less Developed Economies OR Less Developed Nation OR Less Developed Nations OR Less Developed World OR Lesser Developed Countries OR Lesser Developed Nations OR LMIC OR LMICS OR Low GDP OR Low GNP OR Low Gross Domestic OR Low Gross National OR Low Income Countries OR Low Income Country OR Low Income Economies OR Low Income Economy OR Low Income Nations OR Low Income Population OR Low Income Populations OR Lower GDP OR lower gross domestic OR Lower Income Countries OR Lower Income Country OR Lower Income Nations OR Lower Income Population OR Lower Income Populations OR Middle Income Countries OR Middle Income Country OR Middle Income Economies OR Middle Income Nation OR Middle Income Nations OR Middle Income Population OR Middle Income Populations OR Poor Countries OR Poor Country OR Poor Economies OR Poor Economy OR Poor Nation OR Poor Nations OR Poor Population OR Poor Populations OR poor world OR Poorer Countries OR Poorer Economies OR Poorer Economy OR Poorer Nations OR Poorer Population OR Poorer Populations OR Third World OR Transitional Countries OR Transitional Country OR Transitional Economies OR Transitional Economy OR Under Developed Countries OR Under Developed Country OR under developed nations OR Under Developed World OR Under Served Population OR Under Served Populations OR Underdeveloped Countries OR Underdeveloped Country OR

		underdeveloped economies OR underdeveloped nations OR underdeveloped population OR Underdeveloped World OR Underserved Countries OR Underserved Nations OR Underserved Population OR Underserved Populations
#8	Free text:	Afghanistan OR Albania OR Algeria OR American Samoa OR Angola OR Armenia OR Azerbaijan OR Bangladesh OR Belarus OR Byelarus OR Belorussia OR Belize OR Benin OR Bhutan OR Bolivia OR Bosnia OR Botswana OR Brazil OR Bulgaria OR Burma OR Burkina Faso OR Burundi OR Cabo Verde OR Cape Verde OR Cambodia OR Cameroon OR Central African Republic OR Chad OR China OR Colombia OR Comoros OR Comores OR Comoro OR Congo OR Costa Rica OR Côte d'Ivoire OR Cuba OR Djibouti OR Dominica OR Dominican Republic OR Ecuador OR Egypt OR El Salvador OR Equatorial Guinea OR Eritrea OR Ethiopia OR Fiji OR Gabon OR Gambia OR Gaza OR Georgia OR Georgia Republic OR Ghana OR Grenada OR Grenadines OR Guatemala OR Guinea OR Guinea- Bissau OR Guyana OR Haiti OR Herzegovina OR Hercegovina OR Honduras OR India OR Indonesia OR Iran OR Iraq OR Ivory Coast OR Jamaica OR Jordan OR Kazakhstan OR Kenya OR Kiribati OR Democratic People's Republic of Korea OR Kosovo OR Kyrgyz OR Kirghizia OR Kirghiz OR Kyrgyzstan OR Lao PDR OR Laos OR Lebanon OR Lesotho OR Liberia OR Libya OR Macedonia OR Madagascar OR Malawi OR Malay OR Malaya OR Malaysia OR Maldives OR Mali OR Marshall Islands OR Mauritania OR Mauritius OR Mexico OR Micronesia OR Moldova OR Mongolia OR Montenegro OR Morocco OR Mozambique OR Myanmar OR Namibia OR Nepal OR Nicaragua OR Niger OR Nigeria OR Pakistan OR Palau OR Papua New Guinea OR Paraguay OR Peru OR Philippines OR Principe OR Romania OR Ruanda OR Rwanda OR Samoa OR Sao Tome OR Senegal OR Serbia OR Sierra Leone OR Solomon Islands OR Somalia OR South Africa OR South Sudan OR Sri Lanka OR St Lucia OR St Vincent OR Sudan OR Surinam OR Suriname OR Swaziland OR Syria OR Syrian Arab Republic OR Tajikistan OR Tadzhikistan OR Tajikistan OR Tadzhik OR Tanzania OR Thailand OR Timor OR Togo OR Tonga OR Tunisia OR Turkey OR Turkmen OR Turkmenistan OR Tuvalu OR Uganda OR Ukraine OR Uzbek OR Uzbekistan OR Vanuatu OR Venezuela OR Vietnam OR West Bank OR Yemen OR Zambia OR Zimbabwe
#9	#7 OR #8	
#10	#3 AND #6 AND #9	887 Results (290519)
Filter	Only language filter used for PubMed as a preliminary search showed that all recent articles not yet indexed were excluded when filtered for NOT animals and year range	
