# Supplementary material for: Exploration of recovery of people living with severe mental illness (SMI) in low/middle-income countries (LMICs): a scoping review
Source: BMJ Open. 2021 Mar 24;11(3):e045005. doi: 10.1136/bmjopen-2020-045005 (PMC7993175; doi:10.1136/bmjopen-2020-045005)
Supplement: Author's manuscript [file bmjopen-2020-045005.draft_revisions.pdf]

BMJ Open

BMJ Open is committed to open peer review. As part of this commitment we make the peer review history of every article we publish publicly available.

When an article is published we post the peer reviewers' comments and the authors' responses online. We also post the versions of the paper that were used during peer review. These are the versions that the peer review comments apply to.

The versions of the paper that follow are the versions that were submitted during the peer review process. They are not the versions of record or the final published versions. They should not be cited or distributed as the published version of this manuscript.

BMJ Open is an open access journal and the full, final, typeset and author-corrected version of record of the manuscript is available on our site with no access controls, subscription charges or pay-per-view fees (http://bmjopen.bmj.com).

If you have any questions on BMJ Open's open peer review process please email info.bmjopen@bmj.com

BMJ Open

An exploration of recovery of people living with severe mental illness (SMI) in low- and- middle -income countries (LMIC): A scoping review

Journal:	*BMJ Open*
Manuscript ID	bmjopen-2020-045005
Article Type:	Original research
Date Submitted by the Author:	21-Sep-2020

Complete List of Authors:	Gamieldien, Fadia; Alan J Flisher Centre for Public Mental Health, Psychiatry; University of Cape Town Faculty of Health Sciences, Health and Rehabilitation Sciences<br>Galvaan, Roshan ; University of Cape Town Faculty of Health Sciences, Health and Rehabilitation Sciences<br>Myers, Bronwyn; South African Medical Research Council, Alcohol, Tobacco and Other Drug Research Unit; University of Cape Town Faculty of Health Sciences, Division of Addiction Psychiatry, Department of Psychiatry and Mental Health<br>Syed, Zarina; University of Cape Town Faculty of Health Sciences, Health and Rehabilitation Sciences<br>Sorsdahl, Katherine; Alan J Flisher Centre for Public Mental Health, Department of Psychiatry

Keywords:	Adult psychiatry < PSYCHIATRY, Schizophrenia & psychotic disorders < PSYCHIATRY, PUBLIC HEALTH

SCHOLARONE™ Manuscripts

I, the Submitting Author has the right to grant and does grant on behalf of all authors of the Work (as defined in the below author licence), an exclusive licence and/or a non-exclusive licence for contributions from authors who are: i) UK Crown employees; ii) where BMJ has agreed a CC-BY licence shall apply, and/or iii) in accordance with the terms applicable for US Federal Government officers or employees acting as part of their official duties; on a worldwide, perpetual, irrevocable, royalty-free basis to BMJ Publishing Group Ltd ("BMJ") its licensees and where the relevant Journal is co-owned by BMJ to the co-owners of the Journal, to publish the Work in this journal and any other BMJ products and to exploit all rights, as set out in our licence.

The Submitting Author accepts and understands that any supply made under these terms is made by BMJ to the Submitting Author unless you are acting as an employee on behalf of your employer or a postgraduate student of an affiliated institution which is paying any applicable article publishing charge ("APC") for Open Access articles. Where the Submitting Author wishes to make the Work available on an Open Access basis (and intends to pay the relevant APC), the terms of reuse of such Open Access shall be governed by a Creative Commons licence – details of these licences and which Creative Commons licence will apply to this Work are set out in our licence referred to above.

Other than as permitted in any relevant BMJ Author's Self Archiving Policies, I confirm this Work has not been accepted for publication elsewhere, is not being considered for publication elsewhere and does not duplicate material already published. I confirm all authors consent to publication of this Work and authorise the granting of this licence.

For peer review only

An exploration of recovery of people living with severe mental illness (SMI) in low- and-middle -income countries (LMIC): A scoping review

***Fadia Gamieldien*[1]*,* *Roshan Galvaan*[2], *Bronwyn Myers,*[3,4] *Zarina Syed*[2]
***Katherine Sorsdahl*[1]**

1. Alan J. Flisher Centre for Public Mental Health, Department of Psychiatry and Mental Health, University of Cape Town, Cape Town, South Africa

2. Division of Occupational Therapy, Department of Health and Rehabilitation Sciences, University of Cape Town, Cape Town, South Africa

3. Alcohol, Tobacco and Other Drug Research Unit, South African Medical Research Council

4. Division of Addiction Psychiatry, Department of Psychiatry and Mental Health, University of Cape Town, Cape Town, South Africa

Corresponding Author: Ms Fadia Gamieldien

Orcid number 0000-0003-2820-6484

Address: Alan J Flisher Centre for Public Mental Health
Department of Psychiatry and Mental Health
University of Cape Town
46 Sawkins Road
Rondebosch, 7700
Cape Town
South Africa
Fadia.Gamieldien@uct.ac.za
+27 82 974 7468

Bronwyn Myers
0000-0003-0235-6716
Roshan Galvaan
0000-0003-0014-1842
Katherine Sorsdahl
0000-0002-5517-1697
Zarina Syed
0000-0002-5849-9524

Keywords

Recovery, severe mental illness, low-and-middle-income countries, scoping review

Conflict of interest: None

For peer review only
For peer review only - http://bmjopen.bmj.com/site/about/guidelines.xhtml

Word Count 5165

ABSTRACT

Objective

To examine the literature on how recovery of people with SMI is conceptualised in low-and-middle-income countries (LMICs), and in particular what factors are thought to facilitate recovery.

Design:

Scoping review

Data sources and eligibility

We searched 14 electronic databases, handsearched citations and consulted with experts during the period May- December 2019. Eligible studies were independently screened for inclusion and exclusion by two reviewers. Unresolved discrepancies were referred to a third reviewer.

Data extraction and synthesis

All bibliographic data and study characteristics were extracted using a data charting form. Selected studies were analysed through a thematic analysis emerging from extracted data.

Results

The PRISMA flow diagram offers a summary of the results: 4201 titles, 1530 abstracts and 109 full-text articles were screened. Ten articles were selected for inclusion: two from Turkey, two from India, and one each from China, Swaziland, Indonesia, Egypt, South Africa and Vietnam. Although most studies used qualitative methods, data collection and sampling methods were heterogenous. One study reported on service provider perspectives while the rest provided perspectives from a combination of service users and caregivers. Three themes emerged from the data analysis. First, studies frame recovery as a personal journey occurring along a continuum. Second, there was an emphasis on social relationships as a facilitator of recovery. Third, spirituality emerged as both a facilitator and an indicator of recovery. These themes were not mutually exclusive and some overlap exists.

Conclusion

Although there were commanalities with how high income countries describe recovery, we also found differences in conceptualisation. These differences in how recovery was understood reflect the importance of framing the personal recovery concept in relation to local needs and contextual issues found in LMICs. This review highlighted the current sparse evidence base and the need to better understand recovery from SMI in LMICs.

For peer review only - http://bmjopen.bmj.com/site/about/guidelines.xhtml

Protocol information

The scoping review title is registered with the Joanna Briggs Institute. The protocol for this scoping review was published in BMJ Open[1] (doi:10.1136/ bmjopen-2019-032912) (supplementary file 1)

Patient involvement

There is no patient involvement in this scoping review.

Strengths and limitations of the study

- A comprehensive search strategy was used which allowed the literature to be mapped as a basis for further exploration of the concept of recovery in LMICs so as to inform research, policy and practice.
- The review aimed to analyse conceptual and theoretical underpinnings of recovery from SMI as a construct in LMICs and as such interventions and intervention outcomes were not focused on.
- Data synthesis was limited to full text articles available in English only and published between January 1993 and December 2019.
- Grey literature and unpublished studies not available in English were excluded from the scoping review and this practical decision means that potentially relevant papers were excluded.
- Future reviews should search the grey literature extensively as there are non-government organisations active in providing recovery-oriented support and care to people with SMI in LMICs but do not publish in the peer reviewed literature.

For peer review only

INTRODUCTION

Mental, neurological and substance use (MNS) disorders are significant contributors to the global burden of disease, including severe mental illness (SMI). The lifetime prevalence of severe mental illness (SMIs) ranges between 1% and 4%.[2] Although this is relatively low in comparison to the prevalence of common mental disorders, people with SMIs require complex, long-term interventions.[3] Historically most resources to respond to MNS were allocated to specialised psychiatric hospitals, with limited provision of mental health services at primary levels of care.[4]

Deinstitutionalisation initiatives in high income countries (HICs) shifted people out of institutions to community living. The goal of deinstitutionalisation was to promote social inclusion for people with MNS disorders, a goal which remains in progress in low- and-middle- income countries (LMICs). The drive to de-centralise services has led to an increase in premature discharge rates, shorter hospital stays, and repeated relapses.[5 6 9] This is exacerbated by a scarcity of community-based resources[7] to address the psychosocial needs of service users.[4 5 8]

Personal recovery for people with SMI is conceptually distinct from clinical recovery which places more value on medication adherence and symptom remission.[9 10 11] At a time when having a diagnosis of SMI was highly stigmatised and the clinical prognosis was poor, the recovery model was initiated by the mental health consumer and survivor movement in HICs.[12] The notion that recovery was impossible for people with SMI was challenged by people living with schizophrenia, some of whom went on to lead meaningful lives post deinstutionalisation.[13 14]

Psychiatric rehabilitation has highlighted the need to manage the functional and disabling consequences of SMI. However, Deegan (a mental health professional and person living with schizophrenia) says that people do not “get rehabilitated”[12:1] by others, instead they are active participants in their journey to recover new and valued personal meaning and purpose. Personal recovery thus requires a shift beyond treating symptoms to the development of meaning and purpose across the lifespan.[15 9 16]

Recovery means different things to different people, consequently there are multiple interpretations and definitions of recovery. For instance, 17 studies included in a review on the meaning of recovery amongst people living with schizophrenia,[17] described recovery as both a process and an outcome with multidimensional indicators. Recovery depended on

For peer review only - http://bmjopen.bmj.com/site/about/guidelines.xhtml

perceptions related to social support, absence of symptoms, minimal to no relapse, regaining regular functioning and resuming responsibilities. This review found that the process of recovery was influenced by acceptance of illness, developing meaning in life, gaining coping mechanisms and regaining functional abilities.[17-19] The complexity embedded in viewing recovery as a process and an outcome with multidimensional indicators has been acknowledged elsewhere.[20-22] Although recovery cannot be defined in a single way, cognisance must be taken that various elements of recovery identified might be common to peoples' understandings of the experience. Further to this, scholars recommended that clinicians, caregivers and researchers conduct more research to qualitatively explore personal narratives of recovery, develop tools needed to measure recovery and design community-based recovery- oriented services.[17]

Most of the research on personal recovery has emerged from a HIC perspective, with little attention given to describing personal recovery from LMIC perspectives. Of the 17 studies in the aforementioned review,[17] the majority (13) were from HICs (n=13). Similarly, all 97 papers included in a systematic review and narrative synthesis of models of personal recovery[23] originated from just 13 HICs. Although some studies did include ethnic minorities. The value of including perspectives from LMICs and from diverse cultural settings is reflected in the richness and additional themes that emerged from the studies that included ethnic minorities. These additional themes included stigma, spirituality, culture and the collectivist aspects of recovery.[23] Given that recovery is influenced by social, cultural, political, economic factors and respect for human rights, recovery in LMICs is likely to be influenced by socio-cultural context. [24] Consequently, a synthesis of the current body of evidence regarding recovery from SMI of people living in LMICs is warranted so as to offer an alternate perspective to the literature predominantly emanating from HICs; this may allow for more diverse perspectives to emerge.[19 25 26 27] This scoping review thus set out to explore and report on how recovery is described, and the factors that influence personal recovery of people with SMI in LMICs.

METHODS

The protocol for this review[1] provides full details on the methods followed; and the protocol title was registered with the Joanna Briggs Institute. Following Arksey and O'Malley's[27] methodological framework this scoping review comprised six iterative stages, namely: (1) identification of the review question; (2) identification of relevant studies; (3) selection of studies; (4) charting the data; (5) collating, summarising and reporting of results; and (6) consultation with stakeholders. The PRISMA ScR checklist was used as a reporting standard for documenting the process and results.[28 27]

For peer review only - http://bmjopen.bmj.com/site/about/guidelines.xhtml

Stage 1: Identify the research question

We developed a broad research question for our literature search, namely

What is known about recovery from severe mental illness in low-and-middle-income countries?

Stage 2: Identify the relevant studies

A detailed description of the inclusion and exclusion criteria for study selection has been published.[29] Only studies published between 1 January 1993- December 2019 were included. This covers the roughly 25 years of scholarship since Anthony's seminal work on defining personal recovery from mental illness.[15]

To assist us with accessing relevant publications we consulted two mental health service providers working in the field of recovery while conducting an electronic search. A comprehensive search strategy[1] was developed by the first author in collaboration with two librarians (MS and DB). The search strategy and filtering methods were tested using preliminary search terms to comply with searches across different databases. The main filtering methods related to the date range (January 1993- December 2019). (supplementary file 2)

Databases describedin the protocol were searched.[1] Grey literature sources were also pursued through Google Scholar searches, contacting study authors, and connecting with key personnel involved in recovery-focused programmes in LMICs. Reference lists of included full text papers were hand searched for additional sources. Policy documents, conference abstracts, reviews, opinion pieces and commentaries were excluded although they were reviewed to identify published literature.

Stage 3: Literature selection

We followed two independent screening levels for selecting studies for inclusion:(1) title and abstract review; and (2) full-text review. For the first level of review, all citations from the database searches were downloaded into Endnote[30] and duplicates were removed. Thereafter all citations were imported into Rayyan QCRI software[31] which allowed reviewers (FG and ZS) to screen and select titles and abstracts independently according to the inclusion and exclusion criteria. Inter-rater agreement was assessed by calculating Cohen's Kappa. A score of 0.89 was attained, suggesting good interrater reliability.[32]

For peer review only - http://bmjopen.bmj.com/site/about/guidelines.xhtml

One hundred and nine articles (n=109) were selected for full-text review and assessed to determine if they met criteria for study inclusion. Disagreements on study selection were minimal (n=10) and based on interpretations of the outcome, diagnosis or intervention type described in the article. Disagreements were resolved through consensus seeking with a third rater (KS). At this level of screening, inter-rater agreement was calculated as 0.86. Figure 1 summarises the literature search and selection process.

Stage 4: Charting the data

All 109 articles selected for full review were read. The two reviewers (FG and ZS) independently extracted and summarised data based on Joanna Briggs Institute data extraction template.[26 27 37] Extracted information included: 1) study title, 2) author, 3) year of study, 4) country, 5) study population, 6) participant characteristics; 7) number of participants, 8) gender distribution, 9) research question and aim, 10) study methodology, 11) study description, 12) data collection methods, 13) data analysis, 14) outcome measures, 15) results, 16) summary of findings; and 17) definitions and conceptual links of recovery. Supplementary File 2 provides information on the questions applied when charting the data. To ensure rigour, there was flexibility regarding emerging categories for data extraction. Ongoing consultation with the team occurred throughout the process.

The Mixed Methods Appraisal Tool (MMAT) version 2018[33] and the Critical Appraisal Skills Programme (CASP) Qualitative checklist[34] were used to assess the quality of the included studies. Additionally, we assessed compliance to ethical standards as part of the quality appraisal process. Indicators included (a) obtaining human research ethics before study commencement, (b) documentation of consent to participate, and (c) management of vulnerability given that the focus was on people living with an SMI.

Stage 5: Collating, summarising and reporting the results

Collated data were extracted and summarised into a descriptive and narrative synthesis of study characteristics that addressed the review questions. Data were summarised to answer the following questions from LMIC settings how recovery is conceptualised; what the descriptors of recovery are; and what influences recovery. FG completed a qualitative data-driven thematic analysis where findings were coded in qualitative software as recommended by Levac et al.[35] using QSR's International NVivo 11 data analysis software.[36] The coding process was recorded in a memo and reviewed with ZS and KS. All authors contributed to reporting the summarised results.

Stage 6: Consultation

For peer review only

During each stage of the review process the authors consulted with an advisory committee. This advisory committee consists of the research team and key stakeholders including mental health service providers and service users from the public health and non-profit organisation (NPO) sectors. This committee was constituted from the onset of the project and some members are part of a pre-existing group of public mental health specialists. The advisory committee was consulted to gain their opinion on the relevance of the review. They guided access to grey literature, offered their perspectives on the data extraction and charting process, and reviewed the findings. We included the inputs of an advisory committee in this review to enhance its relevance and support collaborative efforts to facilitate consumer participation and public involvement in mental health research.[37 35]

RESULTS

Search findings

Of the 1530 articles originally identified, 109 articles were selected for full text review. Ten were deemed relevant to the research question. Figure 1 outlines the study selection and exclusion process.

Figure 1: Flow Diagram: PRISMA (preferred Reporting for System Reviews and Meta-Analysis (PRISMA)/ PRISMA Extension for Scoping Review-(PRISMA-ScR)[38]

The results are presented in two sections: (i) a summary of characteristics and quality of the included studies and (ii) the results of the thematic analysis.

Description of included studies

Table 1 summarises the ten studies included in this review. All studies were published between 2014 and 2019 and were conducted in: Turkey (n=2), India (n=2), China (n=1), Swaziland (n=1), Indonesia (n=1), Egypt (n=1), South Africa (n=1) and Vietnam (n=1). According to the World Bank classification system, this covered the range of LMICs. Four studies were conducted in upper-middle income countries, one in a middle-income country and five in lower- middle income countries.

The studies followed different study designs (qualitative: n=8; quantitative n=2). Qualitative studies were guided by the following approaches: grounded theory (n=1), phenomenology (n=3), ethnography (n=2) and qualitative descriptive (n=2). In all the qualitative studies, data were collected through semi-structured and unstructured interviews. Five studies focused solely on MHSUs' perspectives[39-43], one described psychiatrists' perspectives[44], and three offered a combination of MHSU and family members perspectives.[45-47] One study reported on multiple stakeholder perspectives by including a combination of MHSUs, family members,

For peer review only

community members and healers.[48] Although there was heterogeneity across studies in terms of participants, MHSUs' perspectives were central to conceptualising recovery in most studies.

All participants were adults and the majority of studies focused on a diagnosis of schizophrenia (n=8) while two studies looked at first episode psychosis.[43 46] The age of participants varied between 21-70, participants had been living with an SMI for between one and forty years and recovery was described as occurring over time. [39-42 45 47 48] The participant's gender profile was described in six studies.[39-42 45 47] In studies where the gender profile was reported, more males were included in the service user groups with the exception of one study which had more females.[41] One study included only female service users.[42] In the two quantitative studies, the majority of caregivers were female.

MHSUs in all of the studies were recruited either directly or indirectly from psychiatric services (n=7) or schizophrenia associations (n=2), with inclusion criteria stipulating a DSMIV-R or ICD 10 coding diagnosis. Two studies stipulated that medication adherence was required for participation in the study.[39 40]

For peer review only

For peer review only - http://bmjopen.bmj.com/site/about/guidelines.xhtml

Table 1: Studies included in the review (n=)

	Authors (year)	Aim(s)	Country; Setting according to World Bank Classification; Recruitment site	Design and methods	Participants
1	Chen et al.[47] (2018)	To develop an instrument to examine the opinions of consumers and their family members regarding autonomous decision making in family matters	Chengdu, China<br>**Upper- middle income country**<br>Psychiatric Hospital	Quantitative<br>Descriptive	**MHSUs: n=182**<br>Female: n= 85<br>Male: n=97<br>**Family members: n= 182**<br>Female: n=103<br>Male: n=79
2	de Wet et al.[43]	To investigate how MHSUs with first episode psychosis experience their recovery	Cape Town, South Africa<br>**Upper- middle income country**<br>Psychiatric Hospital research Unit	Qualitative<br>Descriptive	**MHSUs: n= 7**<br>Gender not reported
3	Gandhi et al.[41] (2019)	To explore patients' perspectives about factors affecting recovery from schizophrenia	Bengaluru, India<br>**Lower- middle-income country**<br>Psychiatric Hospital (Outpatients department)	Qualitative<br>Grounded theory	**MHSUs: n=18**<br>Males: n= 7<br>Females: n=11
4	Gopal et al.[45] (2019)	To identify clients and family members' perspectives of what constitutes recovery from schizophrenia in the Indian context and to examine gender differences in recovery indictors	Chennai, India<br>**Lower- middle-income country**<br>Psychiatric Hospital (Outpatients department)	Quantitative<br>Descriptive	**MHSUs n=100**<br>Males: n=55<br>Females: n=45<br>**Caregivers: n= 80**<br>Male: n=43<br>Female: n=37
5	Guner[39] (2014)	To understand patients views and experiences of schizophrenia	Istanbul, Turkey<br>**Upper- middle income country**<br>Schizophrenia Association	Qualitative<br>Descriptive	**MHSUs: n=9**<br>Male: n=8<br>Female: n=1
6	Humphries et al.[44]	To understand psychiatrists' views on outcomes for people	Danang, Hanoi, HoChi Minh City and Hue, Vietnam	Qualitative<br>Descriptive	**Psychiatrists: n=15**

For peer review only - http://bmjopen.bmj.com/site/about/guidelines.xhtml

	(2015)	with schizophrenia in a developing country	**Middle- income country**<br>Public hospitals		
7	Nxumalo Ngubane et al.[42] (2019)	To explore the experiences and meanings of recovery for Swazi women living with schizophrenia	Swaziland<br>**Lower- middle-income country**<br>Psychiatric Hospital (Outpatients department)	Qualitative<br>Phenomenological	**MHSUs: n=15**<br>Females only
8	Rashed[48] (2015)	To explore the use of spirit-based frameworks to take control over symptoms in their cultural contexts	Dakhla, Western Desert Egypt<br>**Lower- middle-income country**<br>General Hospital with psychiatric service	Qualitative<br>Ethnographic	**MHSUs: n=2**<br>Male: n=1<br>Female: n=1<br>**Family members n= not specified**<br>**Healers**: n=11<br>**Community members**: n=56
9	Soygur[40] (2017)	To identify the factors contributing to recovery, as observed from the perspectives of schizophrenia patients working at a supported employment project	Ankara, Turkey<br>**Upper- middle income country**<br>Schizophrenia Association	Qualitative, Phenomenological;	**MHSUs: n=24**<br>Male: n=15<br>Female: n=9
10	Subandi[46] (2015)	To explore participants' experience of illness and recovery in a Javanese cultural context	Yogyakarta, Java<br>**Lower- middle-income country**<br>Psychiatric Hospital	Qualitative, Ethnographic	**MHSUs: n=7**<br>**Family members**: n= not specified

For peer review only

Table 2: Summary of findings

	Title, Author, Year	Aim(s)	Country and Setting	Key Findings
1	Family Decision Making and Self-Determination Among Consumers With Schizophrenia in China: Cross-Cultural Implications Chen et al, 2018	To develop an instrument to examine the opinions of consumers and their respective family members about autonomous decision making in family matters	Chengdu, China	-Autonomy in decision making contributes to the MHSUs functioning during recovery.<br>-In Asian contexts it is accepted practice that family members make decisions on behalf of the MHSUs especially with regard to money management and community and daily living.<br>-Decisions related to psychiatric care are often deferred to health professionals.<br>-The concept of autonomy for MHSUs includes situations where they endorse and agree with decisions made by family members on their behalf.
2	Hearing their voices: The lived experience of recovery from first episode psychosis in schizophrenia in South Africa.[43]	To investigate how MHSUs with first episode psychosis experience their recovery	Cape Town, South Africa	-Caring for others and being cared for was identified as the biggest contributor to recovery of persons with schizophrenia.<br>Spirituality was seen to build resilience more so than adherence to medication.<br>Understanding the mental illness and managing associated stigma influenced disclosure, especially at work.
3	Perspectives of consumers in India on factors affecting recovery from schizophrenia. Gandhi et al, 2019	To explore patients' perspectives about factors affecting recovery from schizophrenia	Bengaluru, India	-MHSUs in India indicated that recovery was affected by facilitators and barriers related to individual, familial and societal influences.<br>-Holding valued roles in a supportive family structure facilitated the recovery of MHSUs.<br>-MHSUs reported that spirituality and their engagement in prayer and religious rituals anchored them and contributed towards their recovery.

For peer review only - http://bmjopen.bmj.com/site/about/guidelines.xhtml

				-Engaging in activities with others was important for MHSUs as they reported that having meaningful social relationships helped them combat loneliness<br>- Barriers to recovery included side effects of medication, inconsistent treatment approaches and religious beliefs which delayed access to treatment
4	What constitutes recovery in schizophrenia? Client and caregiver perspectives from South India Gopal et al; 2019	To identify clients and family members' perspectives of what constitutes recovery from schizophrenia in Indian context and examine gender differences in recovery indictors	Chennai, India	-Recovery according to caregivers and MHSUs entailed being symptom free; being able to work; and being independent.<br>-Recovery was equated with no longer needing medication.<br>-Recovery was based on MHSUs subjective feelings and experiences and not on the opinions of family members and health professionals.
5	Illness perception in Turkish schizophrenia patients: A qualitative explorative study Guner; 2014	To understand patients views and experiences of schizophrenia	Istanbul, Turkey	-Recovery was variably defined and included a lack of symptoms; being able to work; getting married and having opportunities to became part of community networks.<br>- MHSUs took ownership for their own personal recovery by engaging in meaningful occupations, hobbies and religious practices including prayers.<br>-In Turkey, almost all MHSUs with schizophrenia live with their families<br>For some, having a supportive family facilitated recovery, but recovery was hindered when the family was overprotective.<br>-Recovery was further hindered by difficulties accessing mental health services<br>MHSUs did not disclose their mental illness for fear of stigma

For peer review only - http://bmjopen.bmj.com/site/about/guidelines.xhtml

6	Psychiatrists' perceptions of what determines outcomes for people diagnosed with schizophrenia in Vietnam. Humphries et al; 2015	How psychiatrists view outcomes for people with schizophrenia in a developing country	Danang, Hanoi, HoChi Minh City and Hue, Vietnam	-Access to contemporary treatment was seen as influencing the outcomes for people with schizophrenia. Contemporary treatment is defined as including generic second-generation antipsychotics, staff, facilities and psychosocial interventions.<br>-Psychosocial interventions were not described.<br>-MHSUs feared stigma and this influenced help seeking behaviour.<br>- Psychiatrists reported that in families who hold traditional beliefs, mental illness was attributed to spiritual causes, families access traditional treatment before seeking medical assistance, especially in rural areas.<br>- The extended family system allows family members to share the burden of caring for a MHSU, especially in the absence of formal services<br>-MHSUs were able to access unskilled work in rural parts of Vietnam more readily than MHSUs in urban settings.
7	The experiences and meanings of recovery for Swazi women living with "schizophrenia". Nxumalo Ngubane et al, 2019	To explore the experiences and meanings of recovery for Swazi women living with schizophrenia	District not specified, Swaziland	--In Swaziland, MHSUs referred to any mental illness as "an illness of the brain"<br>-Schizophrenia is not a known term in Swaziland<br>-Diagnostic labels such as schizophrenia were used by health professionals but not shared with MHSUs.<br>-Families and significant others provided emotional and financial support to MHSUs.<br>-The presence of therapeutic rapport between MHSUs and health professionals contributed to personal recovery.
8	From Powerlessness to Control: Psychosis, spirit possession & recovery in the Western desert of Egypt Rashed, 2015	Exploration of the use of spirit-based frameworks to take control over symptoms in their cultural contexts	Dakhla Western Desert, Egypt	-Mental illness was contextualised through spirit-based understandings of the illness and its symptoms.<br>-The relationship MHSUs had with the spirit world was used as a means of describing their recovery journeys<br>- Clinical recovery was contested as it suggested that mental illness' can be cured whereas personal recovery was described as an ongoing process that did not require medical intervention.

For peer review only

				-Personal recovery involved MHSUs gaining control over their symptoms.<br>-MHSUs were positioned as being actively involved in their recovery rather than as passive recipients of services.<br>-Spirituality was foregrounded through the religion of Islam and Quranic healing in the lives of people with schizophrenia.
9	Lessons learned from experiencing Mavi at Café (Blue Horse Café) during six years: A qualitative analysis of factors contributing to recovery from the perspective of schizophrenia patients. Soygur; 2016	"To identify the factors contributing to recovery, as observed from the perspectives of schizophrenia patients working at The Blue Horse Café"	Ankara, Turkey	-This study was located in a therapeutic community and supported employment setting.<br>- Long term relational support between MHSUs and stakeholders involved in health service delivery was seen as being central to recovery.<br>-Recovery was promoted through a number of factors which were embedded in supportive relationships and unrestrictive environments.<br>-MHSUs reported that having work gave them a sense of purpose, responsibility and motivation which added meaning to their lives.
10	Bangkit: The Processes of Recovery from First Episode Psychosis in Java. Subandi; 2015	To explore participants' experience of illness and recovery in a Javanese cultural context	Yogyakarta, Java	-MHSUs viewed their recovery in the cultural context in which they lived<br>-Recovery had different aspects to it and involved medical treatment, family care in the home and integration into natural community settings.<br>-MHSUs reported being guided by spirituality which required their active participation in the performance of Islamic religious practices.

For peer review only

Themes from included studies

Table 2 provides a summary if the key findings of each of the ten included studies. The thematic analysis yielded three themes describing recovery as a personal and complex social process involving healthcare providers, family and community members.[39-45 47 48] These themes, namely (i) recovery is a personal journey occurring along a continuum, (ii) aspects of social relationships supportive of recovery, and (iii) finding meaning and hope through spirituality and religion are elaborated upon below.

1. Recovery is a personal journey occurring along a continuum

The majority of studies included in this review, conceptualised recovery as an ongoing, non-linear process rather than an as an event with a finite end.[39-41 45 49 42 46 48] The age at onset of illness and duration of illness was highlighted[39-42 45 47 48] as being a signal of when the recovery process began. Onset of illness was usually noticed by family members first and they facilitated health-seeking behaviour on behalf of the MHSU.

Some studies[42 44 46-48] defined the starting point for the recovery process as being from the time that people were back in their homes and communities and not from the start of their admission to hospital. This was confirmed by the methodological decisions taken in all of the included studies to not include in-patients or make reference to in-patient services as facilitating recovery. One study described an outpatient service where the programme included psychosocial skills training, family and group therapy sessions and group outings.[39] It is notable that recognising symptoms as cues of relapse was not explored since it appeared that the emphasis was on managing symptoms within the home and community settings after the onset of illness.[41 43 45] This approach occurred after a diagnosis had been established for affected MHSUs.[42 44 46-48]

Since the process of recovery was described from the point of discharge, coping strategies related to managing symptoms of their illness and their re-integration within their families and communities emerged as critical facilitators to recovery. MHSUs needed strategies to facilitate this recovery process. Strategies identified in the studies included the development of self-awareness; being able to self-regulate; spirituality including prayer; attending to basic needs; understanding the mental illness; having purpose and hope for the future; gaining a sense of autonomy; engagement in meaningful occupations; contributing to community; and adopting a positive attitude to living with a mental illness.[39-43 45 47 48] The studies did not describe the development of these coping strategies. It was also noted that learning to self-

For peer review only - http://bmjopen.bmj.com/site/about/guidelines.xhtml

manage facilitated the recovery process.[39 42 46 50] Key aspects that MHSUs had to learn to self-manage included their temperament, learning to live with and derive meaning from having a mental illness and being hopeful for their future.

There was a lack of consensus across the studies regarding the use of medication as an indicator of recovery.[39-42 44 47 45 46 48] Some studies emphasised treatment adherence as the first steps towards recovery,[39-42 44 47] while others viewed the choice to stop taking medication as an indicator of recovery.[45 46 48] When describing recovery as it relates to the medical model and clinical recovery, studies appeared to focus on the individual and described contemporary treatment as including a combination of access to medication, health personnel, mental health services at health facilities and psychosocial interventions.[41 44] According to Rashed,[48] clinical recovery foregrounds biological aspects, implicitly highlighting a curative approach to the illness, often demonstrated through administering medication. However, in exploring psychosis and spirit possession, Rashed[48] found that recovery was not dependent solely on medication or medical intervention. Instead, recovery was conceptualised as an opportunity to expand personal agency in the way MHSU's respond to their illness, with or without medication.[48]

In the study conducted by Subandi,[46] the recovery process involved different phases of participation and integration across social spheres. Recovery as an active process started as MHSUs gained insight into the social isolation that resulted from their mental illness. Social isolation and withdrawal was viewed as the opposite of recovery as it represented inactivity and passivity.[46 48] Across studies, the effort exerted by MHSUs to expand their social interactions from their immediate physical environment, to engaging with family members and other stakeholders in health and community spaces was as an indicator of recovery.[39-43 45-48]

From the aforementioned it can be seen that the people involved in the recovery process included healthcare providers, family and community members. It is also noteworthy that personal recovery required action in a range of interconnected contexts. MHSUs ability to navigate these different environments indicated what was considered as successful recovery. Indicators of recovery described in the selected articles included: being symptom free, returning to work or supported employment, being independent, getting married, being self-sufficient, making decisions, integrating into social or community networks, and involvement in spiritual and religious activities. Through this, recovery was described as active participation in daily activities. This participation was viewed as an indicator of recovery.[39-48]

For peer review only - http://bmjopen.bmj.com/site/about/guidelines.xhtml

2. Aspects of social relationships supportive of recovery

Across included studies, the perspectives and contributions of healthcare providers, family and community members were identified as crucial to the MHSUs recovery process. From these studies it seems that the ways in which these stakeholders engaged with the recovery process potentially facilitated or hindered the recovery journey.[39-48]

Relational support from health professionals was identified as a facilitator of recovery.[39 40 42 43 47] Access to service providers was influenced by the availability of and access to psychiatric treatment.[44 47] Health professionals supported recovery when they communicated clearly, spent time with service users, offered alternative medication options and provided opportunities for engagement in activities.[39 42] On the contrary, the poor continuity of psychiatric care together with limited and brief consultations with a doctor and a focus on access and adherence to medication was seen to limit the recovery process.[39 41 42 48]

Socio-cultural values and variations in explanatory models of mental illness between the MHSUs and health professionals as well as family members influenced views of recovery.[44 47 48] Family members were more likely to seek help from traditional or spiritual healers before seeking contemporary psychiatric treatment.[44 46] The differences between socio-cultural values and explanatory models of mental illness included perceived causes of mental illness, traditional beliefs attributing mental illness to spiritual causes, fear of stigma and community attitudes. For example, discrepancies in the language used to refer to mental illness obscured a common understanding of recovery.[42] MHSUs[42] referred to any mental illness as an "illness of the brain"(p156) while the diagnostic label of schizophrenia was used by health professionals. For one participant the voices of her grandmother was soothing to her but unacceptable to her family and health service providers[42].There was an element of dissonance between how the MHSUs, their families and service providers made sense of the mental illness, with a disconnect occurring around how their symptoms were understood by different parties.[48] When the family, community or health professionals held different understandings and attitudes towards mental illness, recovery was obstructed.

The studies reported that all participants lived with family members or significant others.[39 41-45 48] Although the complexity of large families was acknowledged,[41 44] the extended family supported recovery through sharing the burden of care in the absence of formal health services. The studies highlighted that family and cultural beliefs along with societal values and expectations were used as indicators of recovery[41 46 47]. These indicators were described

For peer review only - http://bmjopen.bmj.com/site/about/guidelines.xhtml

in different ways and included whether the individual could take care of or provide for their family as they recovered.[39 41-45 48]

Families provided emotional and financial support needed by the MHSUs for daily living during their journey of recovery.[39-46 48] While the family assumed responsibility for decision making when the MHSUs were acutely ill, they gave up some of this responsibility as the MHSUs recovered.[39 47 48] The families role as stewards of recovery continued in different ways as they provided flexible access to social networks for MHSUs on an ongoing basis.[39-46 48] Once MHSUs started engaging in spheres such as personal and social functioning, community and daily living and money management; the family's decision making in these spheres decreased.[42 47 51]

The importance of social support was recognised as a facilitator of recovery. MHSUs accessed support from different sources which included professionals, family members, therapeutic community networks and supported employment.[39 44 48] Peer support was mentioned once as an important facilitator of recovery.[41] Professional support was symptom related, family support involved assisting with tangible aspects such as financial management or with support for community reintegration. Different networks thus facilitated recovery as they provided relational and emotional support to MHSUs.

MHSUs described having social connections and being able to engage in activities alone and with others as key components of personal recovery.[41 45] The MHSUs' social identity and social position in relation to their family, their community and broader society was seen to influence their functioning and social inclusion.[41 45] Stigma was mentioned as a barrier to recovery but it was not elaborated on in the studies.[39 42-44]

In four studies, MHSUs reported that being able to work facilitated access to social networks.[39 40 42 43] For MHSUs, recovery meant becoming part of a social network where they felt valued and which allowed them to meaningfully contribute to their family and community, whether this was in paid employment or not.[39 40 42 43] One study focussed on MHSUs working in a therapeutic community and supported employment setting.[40] The emphasis in this study[40] was on the relational support and social interactions provided to MHSUs via the supported employment project. While employment was important as it gave MHSUs the opportunity to contribute to the financial situation of the family, its role as a facilitator of social support networks was foregrounded. Recovery as a social process which is influenced positively by collaboration between MHSUs and stakeholders through long term relational support and encouragement was emphasised.[40]

For peer review only - http://bmjopen.bmj.com/site/about/guidelines.xhtml

3. Finding meaning and hope through spirituality and religion

Spirituality and religion emerged as resources that the MHSUs and caregivers used for understanding mental illness and the recovery process in eight of the ten studies.[39 41-44 46-48] Five studies[39 41 43 44 47] referred to the significance of spirituality and religion. With regards to religion, Islam was referred to in two studies[46 48] and Christianity in one study.[42] Across these studies, spiritual and religious practices were seen to build resilience and were identified as contributing to recovery when they were equally valued by MHSUs, their families and the community.[39 41-44 46-48]

While in recovery, MHSUs underwent internal spiritual struggles when participating in religious practices outside of their homes. The ways in which this was enacted included directly sharing feelings with God through prayer and not through an intermediary; use of religious rituals to relieve stress; surrendering healing to God’s will; resisting spirit possession as an act of faith and personal struggle; and by practically supporting places of worship through engaging in cleaning chores as part of church maintenance.[39 41-43 46 48]

MHSUs relationship with and understanding of the spirit world was seen as an indicator of recovery in one study.[48] Recovery was aided by MHSUs ability to manage the psychotic symptoms of the mental illness while drawing on the philosophical explanations offered by spirituality. In Egypt, spirit possession featured as part of the experience of psychotic phenomenon and psychosis was not necessarily viewed as a medical problem.[48] According to this study, when MHSUs actively engage in accepted individual and communal religious practices, they are acknowledged as positively navigating personal recovery.

MHSUs reported that spirituality and religious practices provided an anchor for MHSUs and their families as it offered hope for recovery through their own efforts while also having faith and trust in a divine power. Engaging in spiritual practices offered MHSUs structured opportunities to relax, relieve their stress and be hopeful about their future.[41 46] Being hopeful about the future required MHSUs to have faith in themselves and their religion as this allowed them to find peace while living with the challenges of having a mental illness.[39 42 43 48] Furthermore, spirituality was viewed as a resource which contributed positively to the recovery process when MHSUs used it to instil hope for the future and as a mechanism for making sense of the mental illness.

For peer review only - http://bmjopen.bmj.com/site/about/guidelines.xhtml

The views held by MHSUs and their family members regarding the influence of spirituality and religious practices on recovery differed from the views held by health professionals.[40-42 44 48] In one study, psychiatrists indicated that religious beliefs and practices could hinder the recovery process particularly where symptoms were attributed to spiritual causes and where traditional treatment was sought before psychiatric treatment.[44] MHSUs viewed professional explanations of mental illness as unhelpful when they discounted the role of spirituality or religion in recovery, especially if they used coercion to support their own ideas of recovery.[42 48] Relying only on medication dispensed by healthcare providers positioned MHSUs as patients and service recipients while adopting a spiritual and cultural interpretation of mental illness, MHSUs were viewed as people who were in control of their illness and who were agents in their own personal recovery process.[46 48] The focus on clinical recovery and the medical interpretation of mental illness minimised the contribution of spirituality to personal recovery.

DISCUSSION

This review contributes to the growing body of research exploring the meaning of recovery from severe mental lillness[16 17 24] by synthesizing qualitative and quantitative research from LMICs. The ten articles selected for inclusion were published between 2014-2019 suggesting recent and possibly growing interest in understanding perceptions of recovery in these contexts. While there was heterogeneity in terms of design and setting, all studies endorsed the power of individual narrative accounts of recovery. This review highlighted commonalities but also differences between how studies from HICs and LMICs (1) conceptualise the recovery process; (2) outlines the individual, interpersonal and social factors that facilitate or impede recovery; (3) define indicators of recovery; and (4) view the role of spirituality and religion in the recovery process, and as an indicator of recovery.

Findings suggest that recovery for MHSUs is conceptualised as a personal, non-linear, and complex process. While this view is echoed in the literature from HICs,[12 17 23 25] the starting point of the process differs between HICs and LMICs. In HICs, MHSUs generally have access to specialised in-patient mental health care and their recovery journeys were thought to begin pre-discharge.[52] In the included studies, none of the MHSUs were in-patients at the time of the study; their recovery journeys were described post discharge. The conceptualisation of recovery as being non-linear in nature incorporates the frequent occurrence of relapses among this populations[5] and explains the growing interest in the socio- cultural contexts where recovery takes place,[53] particularly in LMICs.

For peer review only - http://bmjopen.bmj.com/site/about/guidelines.xhtml

Given the variability in socio-cultural contexts, a range of individual, interpersonal and social factors were found to facilitate or impede the process of recovery. First, these publications highlighted the role of explanatory models of mental illness including pathways to care and perceptions regarding the use of medication in the process of recovery. Explanatory models of mental illness[54] provide a framework for understanding SMI and treatment seeking behaviour in a particular context. Systematic reviews have highlighted how in many instances traditional healers are often the first point of contact in many LMICs, including African countries.[55 56] Unlike literature from HICs, views were mixed regarding the role of clinical recovery emphasising medication adherence. While some studies highlighted the role of medication in facilitating recovery, [57-59] others emphasised the additional importance of having an awareness of socio-cultural and explanatory models of illness as these factors influence MHSUs, caregivers and community members and can either facilitate or impede recovery.[56 60 61]

Second, included studies highlighted how recovery requires long term relational and emotional support provided by family and friends. The role of the family in MHSUs recovery journeys are emphasised differently in studies from HICs and those from LMICs. In HICs, the deinstitutionalisation process shifted care for MHSUs from hospital to community care. This increased the responsibility placed on family members,[62] but in LMICs access to mental healthcare is a challenge which necessitates that families are involved in the MHSUs recovery journey from the onset, given the limited availability of community care services[20].

According to several of the included studies,[39 41 42 44-47] the family, including significant others are responsible for the establishment of supportive living arrangements which involved living with family members, another key facilitator in the process of recovery. These living arrangements seem to reflect norms that are embedded in particular cultures globally and were not viewed as a lack of independence or aspiration. In Western society, gaining independence is seen an indicator of recovery.[12] In contrast, in LMICs, living with supportive family provided MHSUs with opportunities for social cohesion which improved recovery outcomes for MHSUs.[20 63 64] Although supported housing is viewed as a key facilitator of recovery in HICs,[53 65 66] it was not mentioned in any of the included studies described here. Studies in LMICs have examined supported housing for people who are homeless,[67] in the absence of family support. Despite the potentially important role that families can play in the recovery of MHSUs, there is limited literature available on how living with family or extended family facilitates recovery in LMICs.[68]

For peer review only - http://bmjopen.bmj.com/site/about/guidelines.xhtml

Similar to HICs, accessing opportunities to build social connectedness and community relationships emerged as a facilitator to recovery. Although research indicates that people with SMI have poorer social connections than the general population[23] social relationships allows MHSUs to connect with their family, community and work environments. Employment in the skilled or unskilled labour market, was found to promote personal recovery of people with SMIs.[69-71 44 72 29] Although MHSUs have difficulty accessing employment opportunities, in a number of included studies,[40 42 44-47] the ability to work was described as a significant indicator of recovery as well as a means of accessing social networks and supportive relationships which facilitated recovery.

Third, spirituality emerged not only as an important facilitator of the recovery process, but a significant indicator of recovery for MHSUs in LMICs. This is in keeping with a review on religion, spirituality and mental health[73] in HIC and LMICs which concluded that engagement in religious practices was a facilitator of recovery. On an individual level, religion was seen as a source of emotional support,[74] but the influence of religion on family and cultural perspectives and practices, even for ethnic minorities in HICs was not reported on in this review. Included studies in the current review described community religious groups as accessible social networks which provide support to MHSUs and their families.[75] As a facilitator of recovery, spirituality was also seen as a way of finding meaning and hope for MHSUs and their families thought the rituals and social connections embedded in practice.[60 76]

The findings of this scoping review need to be considered in light of some limitations. The review aimed to analyse conceptual and theoretical underpinnings of recovery from SMI in LMICs; as such interventions and intervention outcomes were not focused on. Data synthesis was limited to full text articles available in English and published between January 1993 and December r 2019. This practical decision may have led to potentially relevant unpublished papers in other langauges being excluded.

CONCLUSION

This review aimed to examine the literature on how recovery of people with SMI is conceptualised in LMICs, and factors thought to facilitate the recovery process. In this review, we found that understandings of recovery overlapped with those from HICs, but there were important differences. Unlike HIC where independence and autonomy were emphasised, social connectedness and interdependence were emphasised as important indicators of recovery in LMICs..[20] In addition, family support played a much larger role in LMICs than in HICs where peer support as a strategy for supporting the recovery process is

For peer review only - http://bmjopen.bmj.com/site/about/guidelines.xhtml

emphasised. Additionally, spirituality emerged as both a facilitator and an indicator of recovery. In HICs, spirituality is rarely considered in the context of recovery, whereas interdependence with the spiritual realm is viewed as an important indicator and facilitator of recovery in LMICsThese differences highlight the importance of assessing whether tools and measures of recovery from SMI, developed in HICs capture all of the key indicators of recovery that are important for MHSU living in LMICs. In conclusion, this review highlights the need to expand the recovery concept to be more inclusive of cultural conceptualisatons of recovery and illness in LMICs that extend beyond the individual.

Author Contributions

This scoping review involved the intellectual contributions of all the authors. All authors were involved in developing the review question and conceptualising the approach. FG developed and tested the search terms in consultation with a subject librarian. FG, ZS and KS developed the data extraction guideline, and this was revised based on feedback from the other authors. FG and ZS reviewed all the articles for inclusion and discrepancies were referred to KS. RG, KS and BM provided substantial critique and review of the article prior to submission.

Acknowledgements

The two Faculty of Health Science librarians who assisted with the protocol for this review were Mary Shelton and Dilshaad Brey. Our appreciation also goes to Dr Edina Amponsah-Dacosta for her technical insight during the scoping review process.

Funding

This work was supported by the DELTAS Africa Initiative [DEL-15-01]. The DELTAS Africa Initiative is an independent funding scheme of the African Academy of Sciences (AAS)'s Alliance for Accelerating Excellence in Science in Africa (AESA) and supported by the New Partnership for Africa's Development Planning and Coordinating Agency (NEPAD Agency) with funding from the Wellcome Trust [DEL-15-01] and the UK government. The views expressed in this publication are those of the author(s) and not necessarily those of AAS, NEPAD Agency, WellcomeTrust or the UK government. The funder has no role in the design, writing, or submission of the manuscript. This paper is part of a research project funded by DELTAS, awarded to FG. BM is funded through the South African Medical Research Council.

Disclosure statement

No potential conflict of interest was reported by the authors

For peer review only

Paper context

To our knowledge, this is the first scoping review to examine the English, published peer reviewed literature specifically related to recovery from SMI in LMICs. The findings suggest that there is growing interest in understanding recovery outside of HICs contexts.

References

1. Gamieldien F, Galvaan R, Myers B, et al. Exploration of recovery of people living with severe mental illness (SMI) in low-income and middle-income countries (LMIC): a scoping review protocol. *BMJ Open* 2020;10(2):e032912. doi: 10.1136/bmjopen-2019-032912
2. Kessler RC, Berglund PA, Bruce ML, et al. The prevalence and correlates of untreated serious mental illness. *Health Serv Res* 2001;36(6 Pt 1):987.
3. Vigo D, Thornicroft G, Atun R. Estimating the true global burden of mental illness. *Lancet Psychiatry* 2016;3(2):171-78. doi: 10.1016/S2215-0366(15)00505-2
4. Eaton J, McCay L, Semrau M, et al. Scale up of services for mental health in low-income and middle-income countries. *Lancet* 2011;378(9802):1592-603. doi: https://doi.org/10.1016/S0140-6736(11)60891-X
5. Petersen I, Lund C, Stein DJ. Optimizing mental health services in low-income and middle-income countries. *Curr Opin Psychiatry* 2011;24(4):318-23. doi: 10.1097/YCO.0b013e3283477afb
6. Sfetcu R, Musat S, Haaramo P, et al. Overview of post-discharge predictors for psychiatric re-hospitalisations: a systematic review of the literature. *BMC Psychiatry* 2017;17(1) doi: 10.1186/s12888-017-1386-z
7. Razzouk D. Accommodation and Health Costs of Deinstitutionalized People with Mental Illness Living in Residential Services in Brazil. *Pharmacoecon Open* 2019;3(1):31-42. doi: 10.1007/s41669-018-0078-z [published Online First: 2018/05/02]
8. Lund C, Oosthuizen P, Flisher AJ, et al. Pathways to inpatient mental health care among people with schizophrenia spectrum disorders in South Africa. *Psychiatr Serv* 2010;61(3):235-40. doi: doi:10.1176/ps.2010.61.3.235
9. Slade M. Personal recovery and mental illness: A guide for mental health professionals: Cambridge University Press 2009.
10. Slade M. Mental illness and well-being: the central importance of positive psychology and recovery approaches. *BMC Health Serv Res* 2010;10:26-26. doi: 10.1186/1472-6963-10-26
11. Myers NL, Myers NL. Culture, stress and recovery from schizophrenia: lessons from the field for global mental health. *Cult Med Psychiatry* 2010;34(3):500-28. doi: 10.1007/s11013-010-9186-7
12. Deegan PE. Recovery: The lived experience of rehabilitation. *Psychiatr Rehabil J* 1988;11(4):11.
13. Webb L. The recovery model and complex health needs: what health psychology can learn from mental health and substance misuse service provision. *J Health Psychol* 2012;17(5):731-41. doi: 10.1177/1359105311425276

For peer review only

14. Farkas M. The vision of recovery today: what it is and what it means for services. *World Psychiatry* 2007;6(2):68.
15. Anthony WA. Recovery from mental illness: the guiding vision of the mental health service system in the 1990s. *Psychiatr Rehabil J* 1993;16(4):11.
16. Tew J, Ramon S, Slade M, et al. Social factors and recovery from mental health difficulties: a review of the evidence. *Br J Soc Work* 2012;42(3):443-60.
17. Mathew ST, Nirmala BP, Kommu JVS. Meaning of Recovery Among Persons with Schizophrenia: A Literature Review. *J Psychosoc Rehabil Mental Health* 2018;5(1):73-81. doi: 10.1007/s40737-018-0111-7
18. Kelly M, Lamont S, Brunero S. An Occupational Perspective of the Recovery Journey in Mental Health. *Br J Occup Ther* 2010;73(3):129-35. doi: 10.4276/030802210x12682330090532
19. Cabassa LJ, Nicasio A, Whitley R. Picturing Recovery: A Photovoice Exploration of Recovery Dimensions Among People With Serious Mental Illness. *Psychiatr Serv* 2013;64(9):10.1176/appi.ps.201200503. doi: 10.1176/appi.ps.201200503
20. Aldersey HM, Adeponle AB, Whitley R. Diverse Approaches to Recovery from Severe Mental Illness. The Palgrave Handbook of Sociocultural Perspectives on Global Mental Health2017:109-27.
21. Frost BG, Tirupati S, Johnston S, et al. An Integrated Recovery-oriented Model (IRM) for mental health services: evolution and challenges. *BMC Psychiatry* 2017;17:22. doi: 10.1186/s12888-016-1164-3
22. Lysaker PH, Hamm JA, Hasson-Ohayon I, et al. Promoting recovery from severe mental illness: Implications from research on metacognition and metacognitive reflection and insight therapy. *World J Psychiatry* 2018;8(1):1-11. doi: 10.5498/wjp.v8.i1.1 [published Online First: 2018/03/24]
23. Leamy M, Bird V, Le Boutillier C, et al. Conceptual framework for personal recovery in mental health: systematic review and narrative synthesis. *Br J Psychiatry* 2011;199(6):445-52. doi: 10.1192/bjp.bp.110.083733
24. Llewellyn-Beardsley J, Rennick-Egglestone S, Callard F, et al. Characteristics of mental health recovery narratives: Systematic review and narrative synthesis. *PLoS One* 2019;14:e0214678. doi: 10.1371/journal.pone.0214678
25. Slade M, Longden E. Empirical evidence about recovery and mental health. *BMC Psychiatry* 2015;15:285. doi: 10.1186/s12888-015-0678-4
26. Peters MD, Godfrey CM, Khalil H, et al. Guidance for conducting systematic scoping reviews. *Int J Evid Based Healthc* 2015;13(3):141-46.
27. Arksey H, O'Malley L. Scoping studies: towards a methodological framework. *Int J Soc Res* 2005;8(1):19-32. doi: 10.1080/1364557032000119616
28. Tricco AC, Lillie E, Zarin W, et al. Prisma extension for scoping reviews (prisma-scr): Checklist and explanation. *Ann Intern Med* 2018;169(7):467-73. doi: 10.7326/M18-0850
29. Gamieldien F, van Niekerk L. Street vending in South Africa: An entrepreneurial occupation. *South African Journal of Occupational Therapy* 2017;47(1):24-29.
30. Analytics C. EndNote version X8. San Francisco, CA: Clarivate Analytics, 2018.
31. Ouzzani M, Hammady H, Fedorowicz Z, et al. Rayyan—a web and mobile app for systematic reviews. *Systematic reviews* 2016;5(1):210.

For peer review only - http://bmjopen.bmj.com/site/about/guidelines.xhtml

32. Blackman NJ-M, Koval JJ. Interval estimation for Cohen's kappa as a measure of agreement. *Stat Med* 2000;19(5):723-41. doi: 10.1002/(sici)1097-0258(20000315)19:5<723::Aid-sim379>3.0.Co;2-a
33. Hong QN, Pluye P, Fàbregues S, et al. Mixed methods appraisal tool (MMAT), version 2018. *IC Canadian Intellectual Property Office, Industry Canada* 2018
34. Singh J. Critical appraisal skills programme. *Journal of pharmacology and Pharmacotherapeutics* 2013;4(1):76.
35. Levac D, Colquhoun H, O'Brien KK. Scoping studies: advancing the methodology. *Implement Sci* 2010;5(1):69.
36. Castleberry A. NVivo 10 [software program]. Version 10. QSR International; 2012. *Am J Pharm Educ* 2014;78(1)
37. Morgan DL. Pragmatism as a Paradigm for Social Research. *Qual Inq* 2014;20(8):1045-53. doi: 10.1177/1077800413513733
38. Moher D, Shamseer L, Clarke M, et al. Preferred reporting items for systematic review and meta-analysis protocols (PRISMA-P) 2015 statement. *Systematic reviews* 2015;4(1):1.
39. Güner P. Illness perception in Turkish schizophrenia patients: A qualitative explorative study. *Arch Psychiatr Nurs* 2014;28(6):405-12. doi: 10.1016/j.apnu.2014.08.016
40. Soygür H, Yüksel Avcioğlu MM, Tekin Eraslan P, et al. Lessons learned from experiencing Mavi at Café (Blue Horse Café) during six years: A qualitative analysis of factors contributing to recovery from the perspective of schizophrenia patients. *Turkish Journal of Psychiatry* 2017;28(2):1-7.
41. Gandhi S, Jose D, Desai G. Perspectives of consumers in India on factors affecting recovery from schizophrenia. *Int J Soc Psychiatry* 2019:20764019877425. doi: 10.1177/0020764019877425 [published Online First: 2019/09/24]
42. Nxumalo Ngubane S, McAndrew S, Collier E. The experiences and meanings of recovery for Swazi women living with "schizophrenia". *J Psychiatr Ment Health Nurs* 2019;26(5-6):153-62. doi: 10.1111/jpm.12520 [published Online First: 2019/05/03]
43. de Wet A, Swartz L, Chiliza B. Hearing their voices: The lived experience of recovery from first-episode psychosis in schizophrenia in South Africa. *Int J Soc Psychiatry* 2015;61(1):27-32. doi: 10.1177/0020764014535753 [published Online First: 2014/05/31]
44. Humphries SH, King RJ, Dunne MP, et al. Psychiatrists' perceptions of what determines outcomes for people diagnosed with schizophrenia in Vietnam. *ASEAN Journal of Psychiatry* 2015;16(2):181-92.
45. Gopal S, Mohan G, John S, et al. What constitutes recovery in schizophrenia? Client and caregiver perspectives from South India. *Int J Soc Psychiatry* 2019;0(0):0020764019886339. doi: 10.1177/0020764019886339
46. Subandi MA. Bangkit: The Processes of Recovery from First Episode Psychosis in Java. *Cult Med Psychiatry* 2015;39(4):597-613. doi: 10.1007/s11013-015-9427-x [published Online First: 2015/01/21]
47. Chen Y, Lam CS, Deng H, et al. Family Decision Making and Self-Determination Among Consumers With Schizophrenia in China: Cross-Cultural Implications. *Psychiatr Serv* 2018;69(4):462-68. doi: 10.1176/appi.ps.201500255
48. Rashed MA. From Powerlessness to Control: Psychosis, spirit possession & recovery in the Western desert of Egypt. *Health, Culture & Society* 2015;8(2):10-26. doi: 10.5195/hcs.2015.194

For peer review only

For peer review only - http://bmjopen.bmj.com/site/about/guidelines.xhtml

49. De Wet A, Parker J, Pretorius C. The Spring Foundation: a recovery approach to institutional public mental health services in South Africa. *Perspectives in Public Health* 2019;139(3):123-24. doi: 10.1177/1757913919838767
50. Are we Overdiagnosing Bipolar Disorder?, 2017:698-702.
51. Sweet D, Byng R, Webber M, et al. Personal well-being networks, social capital and severe mental illness: Exploratory study. *Br J Psychiatry* 2018;212(5):308-17. doi: 10.1192/bjp.bp.117.203950
52. van Weeghel J, van Zelst C, Boertien D, et al. Conceptualizations, assessments, and implications of personal recovery in mental illness: A scoping review of systematic reviews and meta-analyses. *Psychiatr Rehabil J* 2019;42(2):169.
53. Doroud N, Fossey E, Fortune T. Place for being, doing, becoming and belonging: A meta-synthesis exploring the role of place in mental health recovery. *Health Place* 2018;52:110-20. doi: 10.1016/j.healthplace.2018.05.008
54. Makanjuola V, Esan Y, Oladeji B, et al. Explanatory model of psychosis: impact on perception of self-stigma by patients in three sub-saharan African cities. *Soc Psychiatry Psychiatr Epidemiol* 2016;51(12):1645-54. doi: 10.1007/s00127-016-1274-8
55. Burns JK, Tomita A. Traditional and religious healers in the pathway to care for people with mental disorders in Africa: a systematic review and meta-analysis. *Soc Psychiatry Psychiatr Epidemiol* 2015;50(6):867-77. doi: 10.1007/s00127-014-0989-7
56. Green B, Colucci E. Traditional healers' and biomedical practitioners' perceptions of collaborative mental healthcare in low- and middle-income countries: A systematic review. *Transcult Psychiatry* 2020;57(1):94-107. doi: 10.1177/1363461519894396 [published Online First: 2020/01/16]
57. Winsper C, Crawford-Docherty A, Weich S, et al. How do recovery-oriented interventions contribute to personal mental health recovery? A systematic review and logic model. *Clin Psychol Rev* 2020;76:101815.
58. Le Boutillier C, Chevalier A, Lawrence V, et al. Staff understanding of recovery-orientated mental health practice: a systematic review and narrative synthesis. *Implement Sci* 2015;10:87. doi: 10.1186/s13012-015-0275-4
59. Chester P, Ehrlich C, Warburton L, et al. What is the work of recovery oriented practice? A systematic literature review. *International journal of mental health nursing* 2016;25(4):270-85.
60. Bhikha AG, Farooq S, Chaudhry N, et al. A systematic review of explanatory models of illness for psychosis in developing countries. *Int Rev Psychiatry* 2012;24(5):450-62.
61. Thornicroft G, Deb T, Henderson C. Community mental health care worldwide: current status and further developments. *World Psychiatry* 2016;15(3):276-86. doi: 10.1002/wps.20349
62. Ward-Griffin C, Schofield R, Vos S, et al. Canadian families caring for members with mental illness: A vicious cycle. *J Fam Nurs* 2005;11(2):140-61.
63. Ran MS, Weng X, Liu YJ, et al. Changes in treatment status of patients with severe mental illness in rural China, 1994-2015. *BJPsych Open* 2019;5(2):e31. doi: 10.1192/bjo.2019.13 [published Online First: 2019/05/10]
64. Chan SKW, Hui CLM, Chang WC, et al. Ten-year follow up of patients with first-episode schizophrenia spectrum disorder from an early intervention service: Predictors of clinical remission and functional recovery. *Schizophr Res* 2019;204:65-71. doi: 10.1016/j.schres.2018.08.022

For peer review only - http://bmjopen.bmj.com/site/about/guidelines.xhtml

65. McPherson P, Krotofil J, Killaspy H. Mental health supported accommodation services: A systematic review of mental health and psychosocial outcomes. *BMC Psychiatry* 2018;18(1) doi: 10.1186/s12888-018-1725-8
66. Carpenter-Song E, Hipolito MMS, Whitley R. 'Right here is an oasis': How 'recovery communities' contribute to recovery for people with serious mental illnesses. *Psychiatric Rehabilitation Journal* 2012;35(6):435-40. doi: 10.1037/h0094576
67. Padmakar A, De Wit EE, Mary S, et al. Supported Housing as a recovery option for long-stay patients with severe mental illness in a psychiatric hospital in South India: Learning from an innovative de-hospitalization process. *PLoS One* 2020;15(4):e0230074. doi: 10.1371/journal.pone.0230074
68. Gopal S, Henderson AR. Trans-cultural study of recovery from severe enduring mental illness in Chennai, India and Perth, Western Australia. *J Psychosoc Rehabil Mental Health* 2015;2(1):51-57.
69. Young DK, Cheng D, Ng P. Predictors of Personal Recovery of People with Severe Mental Illness in a Chinese Society: a Cross-Sectional Study with a Random Sample. *International Journal of Mental Health and Addiction* 2019 doi: 10.1007/s11469-019-00134-w
70. Thomas TL, Prasad Muliyala K, Jayarajan D, et al. Vocational challenges in severe mental illness: A qualitative study in persons with professional degrees. *Asian J Psychiatr* 2019;42:48-54. doi: 10.1016/j.ajp.2019.03.011 [published Online First: 2019/04/07]
71. Doroud N, Fossey E, Fortune T. Recovery as an occupational journey: A scoping review exploring the links between occupational engagement and recovery for people with enduring mental health issues. *Aust Occup Ther J* 2015;62(6):378-92. doi: 10.1111/1440-1630.12238
72. Lund C, Brooke-Sumner C, Baingana F, et al. Social determinants of mental disorders and the Sustainable Development Goals: a systematic review of reviews. *Lancet Psychiatry* 2018;5(4):357-69. doi: 10.1016/s2215-0366(18)30060-9
73. Borthwick CHDKA. The relevance of moral treatment to contemporary mental health care. *Journal of Mental Health* 2001;10(4):427-39. doi: 10.1080/09638230124277
74. Koenig HG. Research on Religion, Spirituality, and Mental Health: A Review. *The Canadian Journal of Psychiatry* 2009;54(5):283-91. doi: 10.1177/070674370905400502
75. Griffith JL, Myers N, Compton MT. How Can Community Religious Groups Aid Recovery for Individuals with Psychotic Illnesses? *Community Ment Health J* 2016;52(7):775-80. doi: 10.1007/s10597-015-9974-1 [published Online First: 2015/12/30]
76. Abdaleati NS, Mohd Zaharim N, Mydin YO. Religiousness and Mental Health: Systematic Review Study. *Journal of Religion and Health* 2016;55(6):1929-37. doi: 10.1007/s10943-014-9896-1

For peer review only

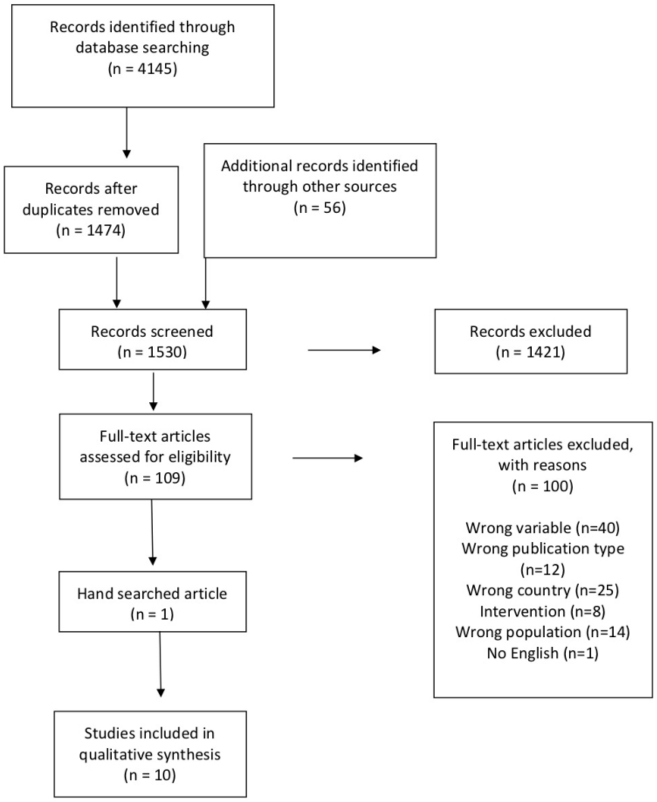

Flow Diagram: PRISMA (preferred Reporting for System Reviews and Meta- Analysis (PRISMA)/ PRISMA Extension for Scoping Review-(PRISMA-ScR

209x297mm (150 x 150 DPI)

Open access Protocol

BMJ Open

Exploration of recovery of people living with severe mental illness (SMI) in low-income and middle-income countries (LMIC): a scoping review protocol

Fadia Gamieldien ,[1,2] Roshan Galvaan,[2] Bronwyn Myers,[3,4] Katherine Sorsdahl[1]

To cite: Gamieldien F, Galvaan R, Myers B, *et al*. Exploration of recovery of people living with severe mental illness (SMI) in low-income and middle-income countries (LMIC): a scoping review protocol. *BMJ Open* 2020;**10**:e032912. doi:10.1136/bmjopen-2019-032912

▶ Prepublication history and additional material for this paper are available online. To view these files, please visit the journal online (http://dx.doi.org/10.1136/bmjopen-2019-032912).

Received 11 July 2019
Revised 24 December 2019
Accepted 06 January 2020

Check for updates

© Author(s) (or their employer(s)) 2020. Re-use permitted under CC BY. Published by BMJ.

[1]Alan J. Flisher Centre for Public Mental Health, Department of Psychiatry and Mental Health, University of Cape Town, Cape Town, South Africa
[2]Division of Occupational Therapy, Department of Health and Rehabilitation Sciences, University of Cape Town, Cape Town, South Africa
[3]Alcohol, Tobacco and Other Drug Research Unit, South African Medical Research Council, Cape Town, South Africa
[4]Division of Addiction Psychiatry, Department of Psychiatry and Mental Health, University of Cape Town, Cape Town, South Africa

Correspondence to
Fadia Gamieldien;
fadia.gamieldien@uct.ac.za

ABSTRACT

Introduction The construct of recovery was conceptualised in high-income countries and its applicability in low-income and middle- income countries is underexplored. A scoping review is proposed to synthesise knowledge, review conceptual overlap and map key elements of recovery from severe mental illness in low-income and middle-income countries. We aim to appraise the literature so as to inform future recovery-oriented services that consider the cultural and contextual influences on recovery from severe mental illness.

Methods and analysis The following electronic databases: MEDLINE via PubMed, SCOPUS (which included contents of Embase), PsycINFO, CINAHL, Africa-Wide Information, PsycARTICLES, Health source: Nursing/Academic Edition, Academic Search Premier and SocINDEX all via the EBSCOHOST platform, the Latin American and Caribbean Health Sciences Literature, the Cochrane Centre Register of Controlled Trials) and grey literature sources will be searched between May and December 2019. Eligible studies will be independently screened for inclusion and exclusion by two reviewers using a checklist developed for this purpose. Studies published between January 1993 and November 2019 that focus on recovery from severe mental illness in a low-income and middle-income country will be included. Findings will be compared and discrepancies will be discussed. Unresolved discrepancies will be referred to a third reviewer. All bibliographic data and study characteristics will be extracted and thematically analysed using a tool developed through an iterative process by the research team. Indicators will be classified according to a predefined conceptual framework and categorised and described using qualitative content analysis.

Ethics and dissemination The review aims to synthesise information from available publications, hence it does not require ethical approval. The results will be disseminated through publications, conference presentations and future workshops with stakeholders involved within the recovery paradigm of mental health policy and practice. The scoping review title is registered with the Joanna Briggs Institute.

Strengths and limitations of this study

- ▶ This scoping review allows for the systematic synthesis of knowledge on recovery from severe mental illness in low-income and middle-income countries (LMICs) in a rigorous and methodological manner.
- ▶ This review will highlight the diversity of severe mental illness since there is no agreed on criteria for including mental illnesses into this category.
- ▶ Knowledge gaps related to recovery as a multidimensional construct in LMICs will be identified.
- ▶ Only conceptual and theoretical underpinnings of recovery as a construct in LMIC will be discussed without a focus on intervention outcomes.
- ▶ Data synthesis will be limited to full-text articles available in English only and published between January 1993 and November 2019. Grey literature and unpublished studies in Latin America will be affected by the decision to exclude articles not available in English.

INTRODUCTION

Mental, neurological and substance use (MNS) disorders contribute significantly to the global burden of disease. Whiteford *et al*[1] report that MNS disorders account for 10% of disability-adjusted life years (DALYs). However, it has been argued that the true global burden of mental health problems may be underestimated by more than one-third and that DALYs are closer to 13%.[2] The lifetime prevalence of severe mental illness (SMIs) ranges between 1% and 4%.[3] Although this is relatively low in comparison to common mental disorders, SMIs are more disabling and require complex, long-term interventions for the person and their family.[2] Given the move towards the deinstitutionalisation of people with psychiatric disorders, shorter hospital stays are resulting in premature discharge rates and repeated relapse, creating a revolving door phenomenon.[4] These frequent hospital readmissions are costly to the healthcare system.[5]

In low-income and middle-income countries (LMICs), many people living with an SMI are not detected and as a result do not receive the treatment they need.[3 6 7] In a service

For peer review only - http://bmjopen.bmj.com/site/about/guidelines.xhtml

For peer review only

BMJ Open: first published as 10.1136/bmjopen-2019-032912 on 3 February 2020. Downloaded from http://bmjopen.bmj.com/ on February 4, 2020 at University of Cape Town Libraries. Protected by copyright.

utilisation review of WHO member countries, it was found that the lifetime prevalence of mental disorders among adults was between 12% and 49%.[6] The review found the treatment gap to be wide across the psychiatric disorders examined. The treatment gap for schizophrenia and non-affective psychosis was 32%. The gap for other disorders was: major depressive disorder, 56%; bipolar disorder, 50%; and alcohol abuse and dependence, 78%.[6] While present in all countries, this treatment gap is greatest in LMICs where there is a need to scale up mental health services.[8]

A systematic review on the scaling up of mental health services in LMICs found that in many countries the majority of resources for mental healthcare are rendered on an inpatient or outpatient basis at large psychiatric hospitals, with little provision of services at primary healthcare facilities or via integration between hospitals and the community.[8] Although there are some nascent programmes to provide services for SMI at primary care facilities, these are generally limited because of a poor integration of mental health into primary healthcare. Additionally, while the lack of coordination between sectors providing mental health services has seen some countries, such as India, have made progress in adopting the District Mental Health Programme, the treatment gap continues in rural areas.[9 10]

Despite the drive for deinstitutionalisation and for the rendering of services at decentralised locations, in many countries most of the mental healthcare budget continues to be allocated to large psychiatric hospitals. In these hospitals, the treatment approach is primarily through medication to alleviate acute clinical symptoms, giving little to no attention to the psychosocial needs of service users.[4 8 11] Unfortunately, the primary focus on clinical recovery and the limited availability of interventions that focus on rehabilitation and personal recovery further exacerbates the revolving door phenomenon and ongoing treatment gap.[4 12]

Recovery-oriented approaches offer an extension to medical models of intervention, as they are not solely focused on symptomatic improvement as a marker of clinical recovery.[13] Personal recovery for people with SMI is conceptually distinct from the present medical definition of recovery, which is clinical in nature and mostly values symptom remission.[14 15] Personal recovery proposes that a person develops new meaning and purpose in their life over the longitudinal course of their illness.[13 16] Psychiatric rehabilitation has highlighted the need to move beyond treating the symptoms of the illness, to treating its functional and disabling consequences as well.[17] Deegan, a mental health professional and person living with schizophrenia, suggested that mental health service users do not 'get rehabilitated' by others, but instead they are active participants in their journey to recover new and valued personal meaning and purpose.[18]

The recovery model was initiated in high-income countries (HICs) with the mental health consumer and survivor movement at a time when having a diagnosis of SMI was highly stigmatised and the prognosis was poor.[18] The idea that people with an SMI cannot recover was challenged by people living with schizophrenia who were leading meaningful lives in their communities after deinstitutionalisation, highlighting that recovery was not dependant on long-term psychiatric treatment.[19 20]For a person living with an SMI, receiving a diagnosis and psychiatric treatment focused on clinical recovery is a necessary step towards recovery, but personal recovery is also critical given the cyclical, non-linear, long-term course of the illness. In Anthony's seminal work,[17] he defined recovery as:

> a deeply personal, unique process of changing ones' attitude, values, feelings, goals, skills and/or roles. It is a way of living a satisfying, hopeful and contributing life even within the limitations caused by illness

Research exploring the meaning of recovery among people living with SMI[21–23] found that recovery means different things to different people, supporting a view that there is no definition of recovery that applies to everyone. A literature review conducted in 2018 explored the meaning of recovery among people living with schizophrenia.[21] This review included 17 studies explaining the subjective meaning of recovery. A majority of the included studies were qualitative, with a few taking a quantitative approach to exploring the meaning of recovery. Of the 17 papers, only four studies were from LMICs (India (n=2), China and South Africa). The results revealed that recovery is complex, non-linear and perceived as both a process and an outcome. The process was viewed as being individual and it was recommended that more consumer perspectives to inform recovery-oriented interventions, which are aligned to individuals' personal goals and aspirations were needed. The complexity embedded in viewing recovery as a process and an outcome with multidimensional indicators has been documented by others.[24–26] Further to this, it was recommended that clinicians, caregivers and researchers explore more qualitative and personal narratives of recovery, the tools needed to measure recovery and the development of community-based recovery- oriented services.[21]

The aforementioned review was limited to recovery in people living with schizophrenia and only included four studies from LMICs. There is current debate around whether people with SMI in LMICs have a better prognosis and a call to re-examine the realities of living with an SMI amidst social, cultural and economic changes in LMICs.[27 28]

Recovery has been viewed as a process or an outcome, with current debates and research seeking to offer conceptual clarity.[12 29] A systematic review conducted by Leamy *et al*[12] led to a proposed conceptual framework for personal recovery in mental illness. The review focused on 97 papers from 13 HICs and identified characteristics, processes and stages of recovery. While the review included individuals from black and minority ethnic groups, it did not include any LMICs.[12] The CHIME

For peer review only - http://bmjopen.bmj.com/site/about/guidelines.xhtml

BMJ Open: first published as 10.1136/bmjopen-2019-032912 on 3 February 2020. Downloaded from http://bmjopen.bmj.com/ on February 4, 2020 at University of Cape Town Libraries. Protected by copyright.

framework emerged from this review process. This framework conceptualises personal recovery to incorporate the following concepts: **c**onnectedness, **h**ope and **o**ptimism about the future, **i**dentity, **m**eaning in life and **e**mpowerment.[12] This highlights that literature on personal recovery is dominated by research conducted in HICs and that there is a gap in understanding recovery from culturally diverse groups.[30]

Research seeking to understand recovery as a process and an outcome is ongoing.[31] The complexity of the concept and the range of factors impacting recovery has been the topic of recent studies. One such focal area has been on the relationship between homelessness among people living with SMIs and the contribution of stable housing towards recovery.[32 33] Additionally, a recent exploration of metacognition and recovery from mental illness challenged conventional treatment to embrace the notion that recovery is unique, individualised and self-directed and that service users need to be active participants in their recovery processes.[26] A call has been made for mental health service users to be partners in conceptualising recovery as a concept and treatment orientation in order to integrate the notion of recovery into mainstream psychiatry.[31]

A scoping review of systematic reviews and meta-analyses conducted by van Weeghel *et al*[29] mapped the concept of recovery and its assessment. It found that recovery is a personal process that is dynamic and evolves over time.[29] Although 25 studies were included in the review, only one study was conducted by authors from India. Conducting a scoping review focusing specifically on studies conducted in LMICs will contribute to the literature on recovery in these specific contexts and will be useful for the following reasons. First, little attention has been given to describing recovery as a personal and individual journey from LMIC perspectives. As recovery for the person with an SMI takes place in a social context through a range of relationships, more understanding about how contexts can help or hinder recovery is needed.[34]

Second, individual meaning making within recovery is influenced by social, political, economic and human rights factors but further research is needed to understand the diversity of recovery narratives from the perspectives of different population groups.[35] A systematic review and narrative analysis synthesising descriptions and models of personal recovery was undertaken to inform the development of a conceptual framework to guide recovery oriented research and practice.[12] Studies conducted in HIC specifically the USA featured predominantly in the review which included 97 papers. The review culminated in the emergence of a conceptual framework to describe the characteristics of recovery, the processes of recovery and the stages of recovery. The authors reported a difference in studies which included ethnic minorities and where more emphasis was placed on stigma, spirituality, culture and the collectivist nature of recovery. They acknowledged that this area is underexplored.[12]

Third, in many African cultures, an individual's personhood is manifested through their agency in relation to spirituality, society and self.[36] This may impact on the person and how they experience mental illness and recovery. Understanding cultural concepts and the dynamics of interconnectedness means recognising that personhood in LMICs is weaved into how people experience health, illness and recovery.[36] Considering the person as a spiritual and social being provides opportunities to collaborate with caregivers and communities in developing appropriate interventions.[36] In countries that are spiritually diverse, multicultural and home to people of different religious backgrounds, it is important to contextualise mental illness and explore health professionals own perspectives on culture, psychiatry and mental health and where mental illness must be contextualised.[37 38] Given the reasons cited previously, further exploration into the recovery journeys of people living with SMI in LMICs and how they view the dimensions of recovery is warranted.[21]

A scoping review is proposed as a means of synthesising knowledge on a broadly defined topic and systematically mapping key concepts, theories, evidence and research gaps in the topic area, while still being rigorous and methodological in its approach.[39 40] While systematic reviews and meta-analyses limit their parameters to research trials and quantitative data synthesis, scoping reviews adopt a broader approach to the narrative integration of evidence.[41] We aim to appraise the definitions of recovery and its current utilisation for people with SMI in LMICs. The review findings could be used to inform future practice applications, which consider the cultural and contextual factors impacting on recovery[15] from SMI outside of HICs. This scoping review title is registered with the Joanna Briggs Institute (JBI) although JBI and the International Prospective Register of Systematic Reviews (PROSPERO) do not provide registry numbers for scoping reviews at this time.[39]

METHODS

A scoping review was deemed as the most suitable method to map existing research on recovery from SMI in LMICs because it allows for the topic to be located, examined, summarised and be presented rapidly and systematically. Additionally, it could serve as a precursor to a systematic review.[42] The proposed review will be conducted using the Arksey and O'Malley,[40] scoping review methodology and will be reported on using the Preferred Reporting Items for Systematic Reviews and Meta-Analyses guidelines[43] with an understanding that there is much development as scoping reviews gain popularity especially in health-related topics.[39 44]

Arksey and O'Malley[40] offer a methodological framework for conducting a scoping review. Their approach describes six stages: (1) identification of the research question, (2) identification of relevant studies, (3) selection of studies, (4) charting the data, (5) collating, summarising

BMJ Open: first published as 10.1136/bmjopen-2019-032912 on 3 February 2020. Downloaded from http://bmjopen.bmj.com/ on February 4, 2020 at University of Cape Town Libraries. Protected by copyright.

For peer review only - http://bmjopen.bmj.com/site/about/guidelines.xhtml

and reporting of results and (6) consultation with stakeholders.[40] The last stage is optional according to Arksey and O'Malley,[40] but others have deemed it a necessary step to aide in methodological rigour.[45] Recommendations to enhance the methodology at each stage will be incorporated into this review.[39 43 45]

Stage 1: identifying the research question

In order to guide the search strategy an iterative process of discussion among authors occurred in order to develop and refine the research question. The question needed to be clear enough to inform the subsequent stages while still reflecting the scope of inquiry.[45] Given that scoping studies are focused on summarising the breadth of evidence on a given topic the authors refined the exploratory question to ask the following:

What is known about recovery from SMIs in LMICs?

This broad question was developed to allow for a comprehensive review of the literature in order to review and appraise the definition of recovery and how it is understood in mental health research and practice in LMICs. While many have written about this in HICs,[23 46] there is a dearth of such information in LMICs .

Stage 2: identifying relevant studies

Given the aim of the study is to identify primary published and unpublished studies which will answer the research question, a comprehensive search strategy was developed to aid this. Two health sciences librarians assisted the first author in the iterative process of developing a search strategy including inclusion and exclusion criteria. Decisions were made about time frames, language, search terms and search strategies. Trial searches were run to assess whether relevant literature could be identified using the proposed strategy. A discussion of the identified databases, search strategy and selection criteria follow.

Databases

Electronic databases will be searched to identify studies published between January 1993 and November 2019. This period was selected as it covers roughly 25 years in the recovery movement. Personal recovery is a construct that has already been defined by Anthony,[17] hence the authors were looking specifically for literature on this construct. Terms used in other disciplines that could also mean recovery were not included as the focus of the search is on recovery as explicitly defined by the recovery movement. Furthermore, recovery knowledge and attitudes among health professionals will be included if reported in the selected studies.

An expert librarian assisted the first author in identifying information sources that are relevant to the research question. The following databases were selected: MEDLINE via PubMed, SCOPUS (which included contents of Embase), PsycINFO, CINAHL, Africa-Wide Information, PsycARTICLES, Health source: Nursing/Academic Edition, Academic Search Premier and SocINDEX all via the EBSCOHOST platform. Additionally, the Latin American and Caribbean Health Sciences Literature, the Cochrane Centre Register of Controlled Trials. Grey literature sources will be identified to expand the search and enhance the data sources. Additional sources will be located by means of handsearching reference lists of relevant papers, contacting study authors, searching trial registers and contacting key personnel involved in recovery-focused programmes in LMICs. In this way, peer-reviewed and grey literature from the biomedical sciences, allied health sciences, social sciences and other disciplinary fields will be included.

Search strategy

Two librarians and the first author developed the eligibility criteria for the scoping review. They identified various definitions of SMIs as well as recovery to include in this search strategy. A list of preliminary search terms and filtering methods was developed. The main filtering methods related to the date range of 25 years (January 1993–November 2019). This period was extended so that new publications would not be missed. The search strategy was refined to include Medical Subject Headings (MeSH terms), filters and Boolean operations to comply with searches across different databases. A search strategy for the PubMed database is included in online supplementary file 1. After the initial search, key words and index terms used across the databases were identified and plotted on a table as indicated below (table 1).

There are no universally accepted guidelines to operationalise the concept of recovery and there is no measure of a gold standard of recovery.[47] The CHIME framework is one proposed guide for the development of recovery measures and identifying recovery outcomes for consideration in clinical practice, but this is limited to HICs. In this review, the key concept is personal recovery as defined by the seminal work of Anthony.[17] In the search strategy, the concept of recovery will be explored through the MeSH terms: Psychiatric Rehabilitation; Mental Health Recovery and Recovery of Function as well as through the free-text terms: Recovery OR recover OR psychosocial rehabilitation OR mental health rehabilitation OR psychiatric rehabilitation.

Selection criteria

Articles will be included in the review if they meet the following inclusion and exclusion criteria (table 2).

Stage 3: study selection

The scoping review guideline for identifying and selecting studies informs article selection and data extraction.[40] Two screening levels are proposed: a title and abstract review and a full-text review. For the first level of screening, two authors (FG and KS) will independently screen the title and abstracts of all retrieved citations against a few select inclusion criteria. Any articles that seem relevant by either or both reviewers will be included in the full-text review. Next, these two reviewers will independently screen the full-text articles to assess whether they meet

BMJ Open: first published as 10.1136/bmjopen-2019-032912 on 3 February 2020. Downloaded from http://bmjopen.bmj.com/ on February 4, 2020 at University of Cape Town Libraries. Protected by copyright.

For peer review only - http://bmjopen.bmj.com/site/about/guidelines.xhtml

Table 1 Search strategy terms

Keyword	Alternative
Recovery	Recovery OR recover OR psychosocial rehabilitation OR mental health rehabilitation OR psychiatric rehabilitation OR mental health recovery OR Recovery of function OR Quality of life
Severe mental illness	Severe mental illness OR bipolar OR delusional disorder OR delusional disorders OR major depressive disorder OR major depressive disorders OR schizophrenia OR manic OR manic-depressive OR paranoid disorder OR paranoid disorders OR psychoses OR psychosis OR psychotic disorder OR psychotic disorders OR schizoaffective disorder OR schizoaffective disorders OR Schizophreniform OR serious mental disorder OR serious mental disorders Schizophrenia Spectrum and Other Psychotic Disorders<br>Bipolar and Related Disorders<br>Depressive Disorder
Low-income and middle- income countries	Deprived Countries OR Deprived Population OR Deprived Populations OR Developing Countries OR Developing Country OR Developing Economies OR Developing Economy OR Developing Nation OR Developing Nations OR Developing Population OR Developing Populations OR Developing World OR LAMI Countries OR LAMI Country OR Less Developed Countries OR Less Developed Country OR Less Developed Economies OR Less Developed Nation OR Less Developed Nations OR Less Developed World OR Lesser Developed Countries OR Lesser Developed Nations OR LMIC OR LMICS OR Low GDP OR Low GNP OR Low Gross Domestic OR Low Gross National OR Low Income Countries OR Low Income Country OR Low Income Economies OR Low Income Economy OR Low Income Nations OR Low Income Population OR Low Income Populations OR Lower GDP OR lower gross domestic OR Lower Income Countries OR Lower Income Country OR Lower Income Nations OR Lower Income Population OR Lower Income Populations OR Middle Income Countries OR Middle Income Country OR Middle Income Economies OR Middle Income Nation OR Middle Income Nations OR Middle Income Population OR Middle Income Populations OR Poor Countries OR Poor Country OR Poor Economies OR Poor Economy OR Poor Nation OR Poor Nations OR Poor Population OR Poor Populations OR poor world OR Poorer Countries OR Poorer Economies OR Poorer Economy OR Poorer Nations OR Poorer Population OR Poorer Populations OR Third World OR Transitional Countries OR Transitional Country OR Transitional Economies OR Transitional Economy OR Under Developed Countries OR Under Developed Country OR under developed nations OR Under Developed World OR Under Served Population OR Under Served Populations OR Underdeveloped Countries OR Underdeveloped Country OR underdeveloped economies OR underdeveloped nations OR underdeveloped population OR Underdeveloped World OR Underserved Countries OR Underserved Nations OR Underserved Population OR Underserved Populations<br>OR<br>Afghanistan OR Albania OR Algeria OR American Samoa OR Angola OR Armenia OR Azerbaijan OR Bangladesh OR Belarus OR Byelarus OR Belorussia OR Belize OR Benin OR Bhutan OR Bolivia OR Bosnia OR Botswana OR Brazil OR Bulgaria OR Burma OR Burkina Faso OR Burundi OR Cabo Verde OR Cape Verde OR Cambodia OR Cameroon OR Central African Republic OR Chad OR China OR Colombia OR Comoros OR Comores OR Comoro OR Congo OR Costa Rica OR Côte d'Ivoire OR Cuba OR Djibouti OR Dominica OR Dominican Republic OR Ecuador OR Egypt OR El Salvador OR Equatorial Guinea OR Eritrea OR Ethiopia OR Fiji OR Gabon OR Gambia OR Gaza OR Georgia OR Georgia Republic OR Ghana OR Grenada OR Grenadines OR Guatemala OR Guinea OR Guinea- Bissau OR Guyana OR Haiti OR Herzegovina OR Hercegovina OR Honduras OR India OR Indonesia OR Iran OR Iraq OR Jamaica OR Jordan OR Kazakhstan OR Kenya OR Kiribati OR Democratic People's Republic of Korea OR Kosovo OR Kyrgyz OR Kirghizia OR Kirghiz OR Kyrgyzstan OR Lao PDR OR Laos OR Lebanon OR Lesotho OR Liberia OR Libya OR Macedonia OR Madagascar OR Malawi OR Malay OR Malaya OR Malaysia OR Maldives OR Mali OR Marshall Islands OR Mauritania OR Mauritius OR Mexico OR Micronesia OR Moldova OR Mongolia OR Montenegro OR Morocco OR Mozambique OR Myanmar OR Namibia OR Nepal OR Nicaragua OR Niger OR Nigeria OR Pakistan OR Palau OR Panama OR Papua New Guinea OR Paraguay OR Peru OR Philippines OR Principe OR Romania OR Ruanda OR Rwanda OR Samoa OR Sao Tome OR Senegal OR Serbia OR Sierra Leone OR Solomon Islands OR Somalia OR South Africa OR South Sudan OR Sri Lanka OR St Lucia OR St Vincent OR Sudan OR Surinam OR Suriname OR Swaziland OR Syria OR Syrian Arab Republic OR Tajikistan OR Tadzhikistan OR Tajikistan OR Tadzhik OR Tanzania OR Thailand OR Timor OR Togo OR Tonga OR Tunisia OR Turkey OR Turkmen OR Turkmenistan OR Tuvalu OR Uganda OR Ukraine OR Uzbek OR Uzbekistan OR Vanuatu OR Venezuela OR Vietnam OR West Bank OR Yemen OR Zambia OR Zimbabwe

BMJ Open: first published as 10.1136/bmjopen-2019-032912 on 3 February 2020. Downloaded from http://bmjopen.bmj.com/ on February 4, 2020 at University of Cape Town Libraries. Protected by copyright.

For peer review only - http://bmjopen.bmj.com/site/about/guidelines.xhtml

Table 2 Inclusion and exclusion criteria

Criteria for inclusion	Criteria for exclusion
▶ Published between January 1993 and November 2019.<br>▶ Qualitative and quantitative empirical (ie, primary research) study designs.<br>▶ Theoretical literature on the construct of recovery.<br>▶ Studies will be included if they have been conducted in LMICs.<br>▶ Peer-reviewed scientific literature.<br>▶ Literature focusing on the population of interest, that is, adults with SMI in LMICs will be included. The diagnosis of SMI includes schizophrenia spectrum disorders, schizoaffective disorders, bipolar affective disorders, major depressive disorder or related psychotic disorders (schizophreniform, delusional disorder, substance induced psychotic disorder or disorders not otherwise specified) as well as those with a comorbid substance use disorder. Studies and reports will be excluded if they do not include the keywords or alternatives as outlined in table 1.<br>▶ Explicit mention of severe mental illness and recovery in LMICS.	▶ The review will be limited to full-text articles written in English. However, the authors acknowledge that this practical decision could mean that potentially relevant papers are missed.<br>▶ Conference abstracts will be excluded although they will be reviewed to see if full reports were published.<br>▶ Children will be excluded as the age range for inclusion is 18 years and older.<br>▶ Duplicate articles from the same study will be excluded.<br>▶ Policy documents will be excluded although they will be reviewed to access research in LMICs that they might have referenced.<br>▶ Studies that occurred only in high-income countries, without an LMIC focus will be excluded.<br>▶ Studies must be conducted in LMIC and not in HIC with participants from LMICs.

LMICs, low-income and middle-income countries.

the inclusion and exclusion criteria. At both levels of screening, inter-rater agreement will be assessed by calculating Cohen's kappa. Any disagreement on study selection will be resolved by discussion with a third rater, who will be a senior member of the research team.

Quality appraisal

The Mixed Methods Appraisal Tool V.2018[48] and the Critical Appraisal Skills Programme Qualitative checklist will be used to assess the quality of included studies although this is not required for a scoping review nor will it influence the inclusion of the study.

Using the aforementioned criteria, the number of studies identified and selected for inclusion in the scoping review will be reported on. A narrative description[39] will accompany the search decision flow chart.[43] The flow chart will show the results from the search, the removal of duplicates, study selection, full retrieval numbers, additional searches from reference lists and a final summary of decisions related to the search decision and an explanation of the review decision process.[39]

Stage 4: charting the data

Next, the two reviewers (FG and KS) will independently extract data from the identified articles and chart it.[40] A draft data charting form will be created in the MS Excel programme to facilitate this process.[40] This form will be pretested and an iterative process of refining the form will be undertaken as the review commences to ensure that all relevant information is extracted.[39]

The form will allow the reviewers to confirm study relevance, record study characteristics and extract information relevant to the review question. The following information will be extracted from the articles: (1) study title; (2) author; (3) study population; (4) participant characteristics; (5) research question; (6) study methodology; (7) study description; (8) intervention type (if applicable); (9) intervention duration (if applicable); (10) outcome measures; 11 summary of findings; (12) definitions of key concepts (recovery and SMIs); (13) conceptual links identified; (14) practice implications and (15) recommendations for further development.

While charting the data, it is important to maintain flexibility around including emerging categories and consulting with the team as this occurs. The final version of the charting form along with definitions of included items will be provided as an appendix to the review. This process will facilitate the timeous completion of the review while also maintaining research rigour.

Stage 5: collating, summarising and reporting the results

All results will be collated, summarised and reported on in order to provide a comprehensive and thorough overview of all the reviewed literature. The authors plan to follow an iterative process and not pre-empt or fit findings into what has already been written about recovery in HICs. This will allow new understandings to be uncovered. While the CHIME framework is considered to offer a comprehensive overview of the process of recovery, it has only been used in HICs with predominantly White populations so its applicability with ethnically and culturally diverse groups is unknown.[12 30] The role of non-government organisations and traditional healers in providing services that facilitate recovery for people living with SMIs will be examined if this arises from the scoping review.

For peer review only - http://bmjopen.bmj.com/site/about/guidelines.xhtml

BMJ Open: first published as 10.1136/bmjopen-2019-032912 on 3 February 2020. Downloaded from http://bmjopen.bmj.com/ on February 4, 2020 at University of Cape Town Libraries. Protected by copyright.

A three-step approach[45] of analysing the data, reporting results and applying meaning to results will be followed to provide methodological rigour. Collating and summarising results will include a summary of the types of studies conducted (including the quantity, types of study design, populations, setting) as well as a thematic analysis of the results. More specifically, we will code how studies describe the concept of recovery from SMI and how it is defined and understood in LMICs. The framework used to report the findings will be developed iteratively through the examination of results. Additionally, the authors will report on the broader implications of the review in terms of research, policy and practice.

Stage 6: consultation

While Arksey and O'Malley[40] view the consultation stage as an optional step, Levac *et al*[45] views it as a requirement to ensure methodological rigour. During each stage of the review process, we will consult with an existing stakeholder advisory committee. This advisory committee consists of the research team and key stakeholders including mental health service providers and service users from within the public health and non-profit organisation sectors. This committee has been constituted from the onset of the project and some members are part of a pre-existing group for public mental health specialists. The advisory committee will be consulted to get their views on the relevance of the review, guidance for accessing grey literature and their perspectives on the data extraction process and preliminary findings. Including an advisory committee in the review is informed by a pragmatic approach[49] where the research is treated as a human experience with the understanding that this consultation process is likely to enhance the relevance of this review. It will also support collaborative efforts to facilitate consumer participation, public involvement, access to information and transfer of knowledge.[45]

Patient and public involvement

There is no patient involvement in the protocol development aspect of the study. Service users forming part of the advisory committee referred to stage 6 will be consulted on findings and dissemination of results.

Ethics and dissemination

Since the scoping review methodology aims at synthesising information from available publications, this study does not require ethical approval. Reviews of primary research allow for the mapping of evidence in fields where the body of literature has not yet been comprehensively reviewed or where working definitions and concepts are still developing. The findings will be made available in different formats to facilitate its dissemination. An article reporting on the results will be submitted for publication in a peer-reviewed journal. It will also be presented at relevant conferences and stakeholder engagements involved in mental health policy and practice. These stakeholders include mental health service users (MHSUs), academics, policy-makers, researcher and clinicians. Findings will also be shared via newsletters, policy briefs and social media forums.

Twitter Fadia Gamieldien @fadiagam

Contributors This protocol was developed by the intellectual contributions of all the authors. All authors were involved in developing the review question and conceptualising the approach. FG developed and tested the search terms in consultation with a subject librarian. FG and KS developed the data extraction guideline, and this was revised based on feedback from the other authors. RG, KS and BM provided substantial critique and review of the draft protocol.

Funding This work was supported by the DELTAS Africa Initiative (DEL-15-01). The DELTAS Africa Initiative is an independent funding scheme of the African Academy of Sciences (AAS)'s Alliance for Accelerating Excellence in Science in Africa (AESA) and supported by the New Partnership for Africa's Development Planning and Coordinating Agency (NEPAD Agency) with funding from the Wellcome Trust (DEL-15-01) and the UK government. BM is funded through the South African Medical Research Council.

Disclaimer The views expressed in this publication are those of the author(s) and not necessarily those of AAS, NEPAD Agency, WellcomeTrust or the UK government.

Competing interests None declared.

Patient consent for publication Not required.

Provenance and peer review Not commissioned; externally peer reviewed.

Open access This is an open access article distributed in accordance with the Creative Commons Attribution 4.0 Unported (CC BY 4.0) license, which permits others to copy, redistribute, remix, transform and build upon this work for any purpose, provided the original work is properly cited, a link to the licence is given, and indication of whether changes were made. See: https://creativecommons.org/licenses/by/4.0/.

ORCID iD
Fadia Gamieldien http://orcid.org/0000-0003-2820-6484

REFERENCES

1 Whiteford HA, Ferrari AJ, Degenhardt L, *et al*. The global burden of mental, neurological and substance use disorders: an analysis from the global burden of disease study 2010. *PLoS One* 2015;10:e0116820.
2 Vigo D, Thornicroft G, Atun R. Estimating the true global burden of mental illness. *Lancet Psychiatry* 2016;3:171–8.
3 Kessler RC, Berglund PA, Bruce ML, *et al*. The prevalence and correlates of untreated serious mental illness. *Health Serv Res* 2001;36:987.
4 Petersen I, Lund C, Stein DJ. Optimizing mental health services in low-income and middle-income countries. *Curr Opin Psychiatry* 2011;24:318–23.
5 Sfetcu R, Musat S, Haaramo P, *et al*. Overview of post-discharge predictors for psychiatric re-hospitalisations: a systematic review of the literature. *BMC Psychiatry* 2017;17.
6 Kohn R, Saxena S, Levav I, *et al*. The treatment gap in mental health care. *Bull World Health Organ* 2004;82:858–66.
7 Demyttenaere K, Bruffaerts R, Posada-Villa J, *et al*. Prevalence, severity, and unmet need for treatment of mental disorders in the World Health Organization world mental health surveys. *JAMA* 2004;291:2581–90.
8 Eaton J, McCay L, Semrau M, *et al*. Scale up of services for mental health in low-income and middle-income countries. *Lancet* 2011;378:1592–603.
9 Thornicroft G, Cooper S, Bortel TV, *et al*. Capacity building in global mental health research. *Harv Rev Psychiatry* 2012;20:13–24.
10 Kumar A. Mental health services in rural India: challenges and prospects. *Health* 2011;03:757–61.
11 Lund C, Oosthuizen P, Flisher AJ, *et al*. Pathways to inpatient mental health care among people with schizophrenia spectrum disorders in South Africa. *PS* 2010;61:235–40.
12 Leamy M, Bird V, Boutillier CL, *et al*. Conceptual framework for personal recovery in mental health: systematic review and narrative synthesis. *Br J Psychiatry* 2011;199:445–52.
13 Slade M. *Personal recovery and mental illness: a guide for mental health professionals*. Cambridge University Press, 2009.

BMJ Open: first published as 10.1136/bmjopen-2019-032912 on 3 February 2020. Downloaded from http://bmjopen.bmj.com/ on February 4, 2020 at University of Cape Town Libraries. Protected by copyright.

For peer review only - http://bmjopen.bmj.com/site/about/guidelines.xhtml

14 Slade M. Mental illness and well-being: the central importance of positive psychology and recovery approaches. *BMC Health Serv Res* 2010;10:26.
15 Myers NL. Culture, stress and recovery from schizophrenia: lessons from the field for global mental health. *Cult Med Psychiatry* 2010;34:500–28.
16 Tew J, Ramon S, Slade M, *et al*. Social factors and recovery from mental health difficulties: a review of the evidence. *Br J Soc Work* 2012;42:443–60.
17 Anthony WA. Recovery from mental illness: the guiding vision of the mental health service system in the 1990s. *Psychiatr Rehabil J* 1993;16:11–23.
18 Deegan PE. Recovery: the lived experience of rehabilitation. *Psychiatr Rehabil J* 1988;11:11–19.
19 Webb L. The recovery model and complex health needs: what health psychology can learn from mental health and substance misuse service provision. *J Health Psychol* 2012;17:731–41.
20 Farkas M. The vision of recovery today: what it is and what it means for services. *World Psychiatry* 2007;6:68.
21 Mathew ST, Nirmala BP, Kommu JVS. Meaning of recovery among persons with schizophrenia: a literature review. *J Psychosoc Rehabil Mental Health* 2018;5:73–81.
22 Kelly M, Lamont S, Brunero S. An occupational perspective of the recovery journey in mental health. *Br J Occup Ther* 2010;73:129–35.
23 Cabassa LJ, Nicasio A, Whitley R. Picturing recovery: a photovoice exploration of recovery dimensions among people with serious mental illness. *Psychiatr Serv* 2013;64:837–42.
24 Aldersey HM, Adeponle AB, Whitley R. Diverse approaches to recovery from severe mental illness. The Palgrave Handbook of sociocultural perspectives on global mental health 2017:109–27.
25 Frost BG, Tirupati S, Johnston S, *et al*. An integrated Recovery-oriented model (IRM) for mental health services: evolution and challenges. *BMC Psychiatry* 2017;17:22.
26 Lysaker PH, Hamm JA, Hasson-Ohayon I, *et al*. Promoting recovery from severe mental illness: implications from research on metacognition and metacognitive reflection and insight therapy. *World J Psychiatry* 2018;8:1–11.
27 Cohen A, Patel V, Thara R, *et al*. Questioning an axiom: better prognosis for schizophrenia in the developing world? *Schizophr Bull* 2008;34:229–44.
28 Patel V, Saxena S, Lund C, *et al*. The Lancet Commission on global mental health and sustainable development. *Lancet* 2018;392:1553–98.
29 van Weeghel J, van Zelst C, Boertien D, *et al*. Conceptualizations, assessments, and implications of personal recovery in mental illness: a scoping review of systematic reviews and meta-analyses. *Psychiatr Rehabil J* 2019;42:169–81.
30 Brijnath B. Applying the CHIME recovery framework in two culturally diverse Australian communities: qualitative results. *Int J Soc Psychiatry* 2015;61:660–7.
31 Leonhardt BL, Huling K, Hamm JA, *et al*. Recovery and serious mental illness: a review of current clinical and research paradigms and future directions. *Expert Rev Neurother* 2017;17:1117–30.
32 Kirst M, Zerger S, Wise Harris D, *et al*. The promise of recovery: narratives of hope among homeless individuals with mental illness participating in a housing first randomised controlled trial in Toronto, Canada: Table 1. *BMJ Open* 2014;4:e004379.
33 Smartt C, Prince M, Frissa S, *et al*. Homelessness and severe mental illness in low- and middle-income countries: scoping review. *BJPsych Open* 2019;5.
34 Schön U-K, Denhov A, Topor A. Social relationships as a decisive factor in recovering from severe mental illness. *Int J Soc Psychiatry* 2009;55:336–47.
35 Llewellyn-Beardsley J, Rennick-Egglestone S, Callard F, *et al*. Characteristics of mental health recovery narratives: systematic review and narrative synthesis. *PLoS One* 2019;14:e0214678.
36 Kpanake L. Cultural concepts of the person and mental health in Africa. *Transcult Psychiatry* 2018;55:198–218.
37 Janse van Rensburg ABR. South African Society of Psychiatrists guidelines for the integration of spirituality in the approach to psychiatric practice. *S Afr J Psychiatr* 2014;20:7–39.
38 Swartz L, Foster D. Images of culture and mental illness : South African psychiatric approaches. *Soc Dyn* 1984;10:17–25.
39 Peters MDJ, Godfrey CM, Khalil H, *et al*. Guidance for conducting systematic scoping reviews. *Int J Evid Based Healthc* 2015;13:141–6.
40 Arksey H, O'Malley L. Scoping studies: towards a methodological framework. *Int J Soc Res Methodol* 2005;8:19–32.
41 Dijkers M. What is a scoping review?. KT Update 2015;4.
42 McKinstry C, Brown T, Gustafsson L. Scoping reviews in occupational therapy: the what, why, and how to. *Aust Occup Ther J* 2014;61:58–66.
43 Tricco AC, Lillie E, Zarin W, *et al*. Prisma extension for scoping reviews (prisma-scr): checklist and explanation. *Ann Intern Med* 2018;169:467–73.
44 Pham MT, Rajić A, Greig JD, *et al*. A scoping review of scoping reviews: advancing the approach and enhancing the consistency. *Res Synth Methods* 2014;5:371–85.
45 Levac D, Colquhoun H, O'Brien KK. Scoping studies: advancing the methodology. *Implement Sci* 2010;5:69.
46 Slade M, Longden E. Empirical evidence about recovery and mental health. *BMC Psychiatry* 2015;15:285.
47 Shanks V, Williams J, Leamy M, *et al*. Measures of personal recovery: a systematic review. *Psychiatr Serv* 2013;64:974–80.
48 Hong QN, Pluye P, Fàbregues S, *et al*. Mixed methods appraisal tool (MMAT), version 2018. *IC Canadian Intellectual Property Office, Industry Canada* 2018.
49 Morgan DL. Pragmatism as a paradigm for social research. *Qual Inq* 2014;20:1045–53.

For peer review only

BMJ Open: first published as 10.1136/bmjopen-2019-032912 on 3 February 2020. Downloaded from http://bmjopen.bmj.com/ on February 4, 2020 at University of Cape Town Libraries. Protected by copyright.

For peer review only - http://bmjopen.bmj.com/site/about/guidelines.xhtml

Supplementary file 1

PubMed Search

Population, Concept, Context (PCC)

**Population:**		
#1	MeSH terms:	Schizophrenia Spectrum and Other Psychotic Disorders<br>Bipolar and Related Disorders<br>Depressive disorder, Major
#2	Free text:	Severe mental illness OR bipolar OR delusional disorder OR delusional disorders OR major depressive disorder OR major depressive disorders OR schizophrenia OR manic OR manic-depressive OR paranoid disorder OR paranoid disorders OR psychoses OR psychosis OR psychotic disorder OR psychotic disorders OR schizoaffective disorder OR schizoaffective disorders OR Schizophreniform OR serious mental disorder OR serious mental disorders
#3	#1 OR #2	
**Concept:**		
#4	MeSH terms:	Psychiatric Rehabilitation<br>Mental Health Recovery<br>Recovery of Function
#5	Free text:	Recovery OR recover OR psychosocial rehabilitation OR mental health rehabilitation OR psychiatric rehabilitation
#6	#4 OR #5	
**Context: Low and Middle Income Countries (LMICS)**		
#7	Free text:	Deprived Countries OR Deprived Population OR Deprived Populations OR Developing Countries OR Developing Country OR Developing Economies OR Developing Economy OR Developing Nation OR Developing Nations OR Developing Population OR Developing Populations OR Developing World OR LAMI Countries OR LAMI Country OR Less Developed Countries OR Less Developed Country OR Less Developed Economies OR Less Developed Nation OR Less Developed Nations OR Less Developed World OR Lesser Developed Countries OR Lesser Developed Nations OR LMIC OR LMICS OR Low GDP OR Low GNP OR Low Gross Domestic OR Low Gross National OR Low Income Countries OR Low Income Country OR Low Income Economies OR Low Income Economy OR Low Income Nations OR Low Income Population OR Low Income Populations OR Lower GDP OR lower gross domestic OR Lower Income Countries OR Lower Income Country OR Lower Income Nations OR Lower Income Population OR Lower Income Populations OR Middle Income Countries OR Middle Income Country OR Middle Income Economies OR Middle Income Nation OR Middle Income Nations OR Middle Income Population OR Middle Income Populations OR Poor Countries OR Poor Country OR Poor Economies OR Poor Economy OR Poor Nation OR Poor Nations OR Poor Population OR Poor Populations OR poor world OR Poorer Countries OR Poorer Economies OR Poorer Economy OR Poorer Nations OR Poorer Population OR Poorer Populations OR Third World OR Transitional Countries OR Transitional Country OR Transitional Economies OR Transitional Economy OR Under Developed Countries OR Under Developed Country OR under developed nations OR Under Developed World OR Under Served Population OR Under Served Populations OR Underdeveloped Countries OR Underdeveloped Country OR

		underdeveloped economies OR underdeveloped nations OR underdeveloped population OR Underdeveloped World OR Underserved Countries OR Underserved Nations OR Underserved Population OR Underserved Populations
#8	Free text:	Afghanistan OR Albania OR Algeria OR American Samoa OR Angola OR Armenia OR Azerbaijan OR Bangladesh OR Belarus OR Byelarus OR Belorussia OR Belize OR Benin OR Bhutan OR Bolivia OR Bosnia OR Botswana OR Brazil OR Bulgaria OR Burma OR Burkina Faso OR Burundi OR Cabo Verde OR Cape Verde OR Cambodia OR Cameroon OR Central African Republic OR Chad OR China OR Colombia OR Comoros OR Comores OR Comoro OR Congo OR Costa Rica OR Côte d'Ivoire OR Cuba OR Djibouti OR Dominica OR Dominican Republic OR Ecuador OR Egypt OR El Salvador OR Equatorial Guinea OR Eritrea OR Ethiopia OR Fiji OR Gabon OR Gambia OR Gaza OR Georgia OR Georgia Republic OR Ghana OR Grenada OR Grenadines OR Guatemala OR Guinea OR Guinea- Bissau OR Guyana OR Haiti OR Herzegovina OR Hercegovina OR Honduras OR India OR Indonesia OR Iran OR Iraq OR Ivory Coast OR Jamaica OR Jordan OR Kazakhstan OR Kenya OR Kiribati OR Democratic People's Republic of Korea OR Kosovo OR Kyrgyz OR Kirghizia OR Kirghiz OR Kyrgyzstan OR Lao PDR OR Laos OR Lebanon OR Lesotho OR Liberia OR Libya OR Macedonia OR Madagascar OR Malawi OR Malay OR Malaya OR Malaysia OR Maldives OR Mali OR Marshall Islands OR Mauritania OR Mauritius OR Mexico OR Micronesia OR Moldova OR Mongolia OR Montenegro OR Morocco OR Mozambique OR Myanmar OR Namibia OR Nepal OR Nicaragua OR Niger OR Nigeria OR Pakistan OR Palau OR Papua New Guinea OR Paraguay OR Peru OR Philippines OR Principe OR Romania OR Ruanda OR Rwanda OR Samoa OR Sao Tome OR Senegal OR Serbia OR Sierra Leone OR Solomon Islands OR Somalia OR South Africa OR South Sudan OR Sri Lanka OR St Lucia OR St Vincent OR Sudan OR Surinam OR Suriname OR Swaziland OR Syria OR Syrian Arab Republic OR Tajikistan OR Tadzhikistan OR Tajikistan OR Tadzhik OR Tanzania OR Thailand OR Timor OR Togo OR Tonga OR Tunisia OR Turkey OR Turkmen OR Turkmenistan OR Tuvalu OR Uganda OR Ukraine OR Uzbek OR Uzbekistan OR Vanuatu OR Venezuela OR Vietnam OR West Bank OR Yemen OR Zambia OR Zimbabwe
#9	#7 OR #8	
#10	#3 AND #6 AND #9	887 Results (290519)
Filter	Only language filter used for PubMed as a preliminary search showed that all recent articles not yet indexed were excluded when filtered for NOT animals and year range	

For peer review only

Preferred Reporting Items for Systematic reviews and Meta-Analyses extension for Scoping Reviews (PRISMA-ScR) Checklist

SECTION	ITEM	PRISMA-ScR CHECKLIST ITEM	REPORTED ON PAGE #
**TITLE**			
Title	1	Identify the report as a scoping review.	1
**ABSTRACT**			
Structured summary	2	Provide a structured summary that includes (as applicable): background, objectives, eligibility criteria, sources of evidence, charting methods, results, and conclusions that relate to the review questions and objectives.	2
**INTRODUCTION**			
Rationale	3	Describe the rationale for the review in the context of what is already known. Explain why the review questions/objectives lend themselves to a scoping review approach.	6
Objectives	4	Provide an explicit statement of the questions and objectives being addressed with reference to their key elements (e.g., population or participants, concepts, and context) or other relevant key elements used to conceptualize the review questions and/or objectives.	1,6
**METHODS**			
Protocol and registration	5	Indicate whether a review protocol exists; state if and where it can be accessed (e.g., a Web address); and if available, provide registration information, including the registration number.	3, JBI supplementary file
Eligibility criteria	6	Specify characteristics of the sources of evidence used as eligibility criteria (e.g., years considered, language, and publication status), and provide a rationale.	1,6
Information sources*	7	Describe all information sources in the search (e.g., databases with dates of coverage and contact with authors to identify additional sources), as well as the date the most recent search was executed.	7
Search	8	Present the full electronic search strategy for at least 1 database, including any limits used, such that it could be repeated.	Supplementary file
Selection of sources of evidence†	9	State the process for selecting sources of evidence (i.e., screening and eligibility) included in the scoping review.	7, 8
Data charting process‡	10	Describe the methods of charting data from the included sources of evidence (e.g., calibrated forms or forms that have been tested by the team before their use, and whether data charting was done independently or in duplicate) and any processes for obtaining and confirming data from investigators.	8
Data items	11	List and define all variables for which data were sought and any assumptions and simplifications made.	8
Critical appraisal of individual	12	If done, provide a rationale for conducting a critical appraisal of included sources of evidence; describe	Click here to enter text.

For peer review only - http://bmjopen.bmj.com/site/about/guidelines.xhtml

SECTION	ITEM	PRISMA-ScR CHECKLIST ITEM	REPORTED ON PAGE #
sources of evidence§		the methods used and how this information was used in any data synthesis (if appropriate).	
Synthesis of results	13	Describe the methods of handling and summarizing the data that were charted.	8, supplementary file
**RESULTS**			
Selection of sources of evidence	14	Give numbers of sources of evidence screened, assessed for eligibility, and included in the review, with reasons for exclusions at each stage, ideally using a flow diagram.	9
Characteristics of sources of evidence	15	For each source of evidence, present characteristics for which data were charted and provide the citations.	9
Critical appraisal within sources of evidence	16	If done, present data on critical appraisal of included sources of evidence (see item 12).	Click here to enter text.
Results of individual sources of evidence	17	For each included source of evidence, present the relevant data that were charted that relate to the review questions and objectives.	11-12
Synthesis of results	18	Summarize and/or present the charting results as they relate to the review questions and objectives.	13-15
**DISCUSSION**			
Summary of evidence	19	Summarize the main results (including an overview of concepts, themes, and types of evidence available), link to the review questions and objectives, and consider the relevance to key groups.	17-21
Limitations	20	Discuss the limitations of the scoping review process.	3, 24
Conclusions	21	Provide a general interpretation of the results with respect to the review questions and objectives, as well as potential implications and/or next steps.	24
**FUNDING**			
Funding	22	Describe sources of funding for the included sources of evidence, as well as sources of funding for the scoping review. Describe the role of the funders of the scoping review.	25

JBI = Joanna Briggs Institute; PRISMA-ScR = Preferred Reporting Items for Systematic reviews and Meta-Analyses extension for Scoping Reviews.

* Where *sources of evidence* (see second footnote) are compiled from, such as bibliographic databases, social media platforms, and Web sites.

† A more inclusive/heterogeneous term used to account for the different types of evidence or data sources (e.g., quantitative and/or qualitative research, expert opinion, and policy documents) that may be eligible in a scoping review as opposed to only studies. This is not to be confused with *information sources* (see first footnote).

‡ The frameworks by Arksey and O'Malley (6) and Levac and colleagues (7) and the JBI guidance (4, 5) refer to the process of data extraction in a scoping review as data charting.

§ The process of systematically examining research evidence to assess its validity, results, and relevance before using it to inform a decision. This term is used for items 12 and 19 instead of "risk of bias" (which is more applicable to systematic reviews of interventions) to include and acknowledge the various sources of evidence that may be used in a scoping review (e.g., quantitative and/or qualitative research, expert opinion, and policy document).

From: Tricco AC, Lillie E, Zarin W, O'Brien KK, Colquhoun H, Levac D, et al. PRISMA Extension for Scoping Reviews (PRISMAScR): Checklist and Explanation. Ann Intern Med. 2018;169:467–473. doi: 10.7326/M18-0850.

For peer review only - http://bmjopen.bmj.com/site/about/guidelines.xhtml

BMJ Open

An exploration of recovery of people living with severe mental illness (SMI) in low- and- middle -income countries (LMIC): A scoping review

Journal:	*BMJ Open*
Manuscript ID	bmjopen-2020-045005.R1
Article Type:	Original research
Date Submitted by the Author:	02-Feb-2021
Complete List of Authors:	Gamieldien, Fadia; Alan J Flisher Centre for Public Mental Health, Psychiatry; University of Cape Town Faculty of Health Sciences, Health and Rehabilitation Sciences<br>Galvaan, Roshan ; University of Cape Town Faculty of Health Sciences, Health and Rehabilitation Sciences<br>Myers, Bronwyn; South African Medical Research Council, Alcohol, Tobacco and Other Drug Research Unit; University of Cape Town Faculty of Health Sciences, Division of Addiction Psychiatry, Department of Psychiatry and Mental Health<br>Syed, Zarina; University of Cape Town Faculty of Health Sciences, Health and Rehabilitation Sciences<br>Sorsdahl, Katherine; Alan J Flisher Centre for Public Mental Health, Department of Psychiatry
**Primary Subject Heading**:	Mental health
Secondary Subject Heading:	Rehabilitation medicine, Mental health, Health services research, Public health
Keywords:	Adult psychiatry < PSYCHIATRY, Schizophrenia & psychotic disorders < PSYCHIATRY, PUBLIC HEALTH

SCHOLARONE™ Manuscripts

I, the Submitting Author has the right to grant and does grant on behalf of all authors of the Work (as defined in the below author licence), an exclusive licence and/or a non-exclusive licence for contributions from authors who are: i) UK Crown employees; ii) where BMJ has agreed a CC-BY licence shall apply, and/or iii) in accordance with the terms applicable for US Federal Government officers or employees acting as part of their official duties; on a worldwide, perpetual, irrevocable, royalty-free basis to BMJ Publishing Group Ltd ("BMJ") its licensees and where the relevant Journal is co-owned by BMJ to the co-owners of the Journal, to publish the Work in this journal and any other BMJ products and to exploit all rights, as set out in our licence.

The Submitting Author accepts and understands that any supply made under these terms is made by BMJ to the Submitting Author unless you are acting as an employee on behalf of your employer or a postgraduate student of an affiliated institution which is paying any applicable article publishing charge ("APC") for Open Access articles. Where the Submitting Author wishes to make the Work available on an Open Access basis (and intends to pay the relevant APC), the terms of reuse of such Open Access shall be governed by a Creative Commons licence – details of these licences and which Creative Commons licence will apply to this Work are set out in our licence referred to above.

Other than as permitted in any relevant BMJ Author's Self Archiving Policies, I confirm this Work has not been accepted for publication elsewhere, is not being considered for publication elsewhere and does not duplicate material already published. I confirm all authors consent to publication of this Work and authorise the granting of this licence.

For peer review only

An exploration of recovery of people living with severe mental illness (SMI) in low- and-middle -income countries (LMIC): A scoping review

***Fadia Gamieldien*[1]*,** *Roshan Galvaan*[2], *Bronwyn Myers,*[3,4] *Zarina Syed*[2]

***Katherine Sorsdahl*[1]**

1. Alan J. Flisher Centre for Public Mental Health, Department of Psychiatry and Mental Health, University of Cape Town, Cape Town, South Africa

2. Division of Occupational Therapy, Department of Health and Rehabilitation Sciences, University of Cape Town, Cape Town, South Africa

3. Alcohol, Tobacco and Other Drug Research Unit, South African Medical Research Council

4. Division of Addiction Psychiatry, Department of Psychiatry and Mental Health, University of Cape Town, Cape Town, South Africa

Corresponding Author: Ms Fadia Gamieldien

Orcid number 0000-0003-2820-6484

Address: Alan J Flisher Centre for Public Mental Health

Department of Psychiatry and Mental Health

University of Cape Town

46 Sawkins Road

Rondebosch, 7700

Cape Town

South Africa

Fadia.Gamieldien@uct.ac.za

+27 82 974 7468

Bronwyn Myers
0000-0003-0235-6716
Roshan Galvaan
0000-0003-0014-1842
Katherine Sorsdahl
0000-0002-5517-1697
Zarina Syed
0000-0002-5849-9524

Keywords

Recovery, severe mental illness, low-and-middle-income countries, scoping review

Conflict of interest: None

For peer review only - http://bmjopen.bmj.com/site/about/guidelines.xhtml

ABSTRACT

Objective

To examine the literature on how recovery of people with SMI is conceptualised in low-and-middle-income countries (LMICs), and in particular what factors are thought to facilitate recovery.

Design:

Scoping review

Data sources and eligibility

We searched 14 electronic databases, handsearched citations and consulted with experts during the period May- December 2019. Eligible studies were independently screened for inclusion and exclusion by two reviewers. Unresolved discrepancies were referred to a third reviewer.

Data extraction and synthesis

All bibliographic data and study characteristics were extracted using a data charting form. Selected studies were analysed through a thematic analysis emerging from extracted data.

Results

The PRISMA flow diagram offers a summary of the results: 4201 titles, 1530 abstracts and 109 full-text articles were screened. Ten articles were selected for inclusion: two from Turkey, two from India, and one each from China, Swaziland, Indonesia, Egypt, South Africa and Vietnam. Although most studies used qualitative methods, data collection and sampling methods were heterogenous. One study reported on service provider perspectives while the rest provided perspectives from a combination of service users and caregivers. Three themes emerged from the data analysis. First, studies frame recovery as a personal journey occurring along a continuum. Second, there was an emphasis on social relationships as a facilitator of recovery. Third, spirituality emerged as both a facilitator and an indicator of recovery. These themes were not mutually exclusive and some overlap exists.

Conclusion

Although there were commanalities with how high income countries describe recovery, we also found differences in conceptualisation. These differences in how recovery was understood reflect the importance of framing the personal recovery concept in relation to local needs and contextual issues found in LMICs. This review highlighted the current sparse evidence base and the need to better understand recovery from SMI in LMICs.

For peer review only - http://bmjopen.bmj.com/site/about/guidelines.xhtml

Protocol information

The scoping review title is registered with the Joanna Briggs Institute. The protocol for this scoping review was published in BMJ Open (doi:10.1136/ bmjopen-2019-032912) (supplementary file 1)

Strengths and limitations of the study

- A comprehensive search strategy was used which allowed the literature to be mapped as a basis for further exploration of the concept of recovery in LMICs so as to inform research, policy and practice.
- The review aimed to analyse conceptual and theoretical underpinnings of recovery from SMI as a construct in LMICs and as such interventions and intervention outcomes were not focused on.
- Data synthesis was limited to full text articles available in English only and published between January 1993 and December 2019.
- Grey literature and unpublished studies not available in English were excluded from the scoping review and this practical decision means that potentially relevant papers were excluded.
- Future reviews should search the grey literature extensively as there are non-government organisations active in providing recovery-oriented support and care to people with SMI in LMICs but do not publish in the peer reviewed literature.

For peer review only

INTRODUCTION

Mental, neurological and substance use (MNS) disorders are significant contributors to the global burden of disease, including severe mental illness (SMI). The lifetime prevalence of severe mental illness (SMIs) ranges between 1% and 4%.[1] Although this is relatively low in comparison to the prevalence of common mental disorders, people with SMIs require complex, long-term interventions.[2] Historically most resources to respond to MNS were allocated to specialised psychiatric hospitals, with limited provision of mental health services at primary levels of care.[3]

Deinstitutionalisation initiatives in high income countries (HICs) shifted people out of institutions to community living. The goal of deinstitutionalisation was to promote social inclusion for people with MNS disorders, a goal which remains in progress in low- and-middle- income countries (LMICs). The drive to de-centralise services has led to an increase in premature discharge rates, shorter hospital stays, and repeated relapses. [4-6] This is exacerbated by a scarcity of community-based resources[7] to address the psychosocial needs of service users.[3 4 8]

Personal recovery for people with SMI is conceptually distinct from clinical recovery which places more value on medication adherence and symptom remission.[6 9 10] At a time when having a diagnosis of SMI was highly stigmatised and the clinical prognosis was poor, the recovery model was initiated by the mental health consumer and survivor movement in HICs.[11] The notion that recovery was impossible for people with SMI was challenged by people living with schizophrenia, some of whom went on to lead meaningful lives post deinstutionalisation.[12 13]

Psychiatric rehabilitation has highlighted the need to manage the functional and disabling consequences of SMI. However, Deegan (a mental health professional and person living with schizophrenia) says that people do not “get rehabilitated”[11:1] by others, instead they are active participants in their journey to recover new and valued personal meaning and purpose. Personal recovery thus requires a shift beyond treating symptoms to the development of meaning and purpose across the lifespan.[6 14 15]

Recovery means different things to different people, consequently there are multiple interpretations and definitions of recovery. For instance, 17 studies included in a review on the meaning of recovery amongst people living with schizophrenia,[16] described recovery as both a process and an outcome with multidimensional indicators. Recovery depended on

For peer review only - http://bmjopen.bmj.com/site/about/guidelines.xhtml

perceptions related to social support, absence of symptoms, minimal to no relapse, regaining regular functioning and resuming responsibilities. This review found that the process of recovery was influenced by acceptance of illness, developing meaning in life, gaining coping mechanisms and regaining functional abilities.[16-18] The complexity embedded in viewing recovery as a process and an outcome with multidimensional indicators has been acknowledged elsewhere.[19-21] Although recovery cannot be defined in a single way, cognisance must be taken that various elements of recovery identified might be common to peoples' understandings of the experience. Further to this, scholars recommended that clinicians, caregivers and researchers conduct more research to qualitatively explore personal narratives of recovery, develop tools needed to measure recovery and design community-based recovery- oriented services.[16]

Most of the research on personal recovery has emerged from a HIC perspective, with little attention given to describing personal recovery from LMIC perspectives. Of the 17 studies in the aforementioned review,[16] the majority (13) were from HICs (n=13). Similarly, all 97 papers included in a systematic review and narrative synthesis of models of personal recovery[22] originated from just 13 HICs. Although some studies did include ethnic minorities. The value of including perspectives from LMICs and from diverse cultural settings is reflected in the richness and additional themes that emerged from the studies that included ethnic minorities. These additional themes included stigma, spirituality, culture and the collectivist aspects of recovery.[22] Given that recovery is influenced by social, cultural, political, economic factors and respect for human rights, recovery in LMICs is likely to be influenced by socio-cultural context.[23] Consequently, a synthesis of the current body of evidence regarding recovery from SMI of people living in LMICs is warranted so as to offer an alternate perspective to the literature predominantly emanating from HICs; this may allow for more diverse perspectives to emerge.[18 24-26] This scoping review thus set out to explore and report on how recovery is described, and the factors that influence personal recovery of people with SMI in LMICs.

METHODS

The protocol for this review[27] (supplementary file 1) provides full details on the methods followed, and the protocol title was registered with the Joanna Briggs Institute. Following Arksey and O'Malley's[25] methodological framework this scoping review comprised six iterative stages, namely: (1) identification of the review question; (2) identification of relevant studies; (3) selection of studies; (4) charting the data; (5) collating, summarising and reporting of results; and (6) consultation with stakeholders. The PRISMA ScR checklist was used as a reporting standard for documenting the process and results.[25 28]

For peer review only - http://bmjopen.bmj.com/site/about/guidelines.xhtml

Stage 1: Identify the research question

We developed a broad research question for our literature search, namely

What is known about recovery from severe mental illness in low-and-middle-income countries?

Stage 2: Identify the relevant studies

A detailed description of the inclusion and exclusion criteria for study selection has been published.[27] (supplementary file 1) Only studies published between 1 January 1993-December 2019 were included. This covers the roughly 25 years of scholarship since Anthony's seminal work on defining personal recovery from mental illness.[14]

To assist us with accessing relevant publications we consulted two mental health service providers working in the field of recovery while conducting an electronic search. A comprehensive search strategy[27] was developed by the first author in collaboration with two librarians (MS and DB). The search strategy and filtering methods were tested using preliminary search terms to comply with searches across different databases. The main filtering methods related to the date range (January 1993- December 2019). (supplementary file 2)

Databases describedin the protocol were searched.[27] Grey literature sources were also pursued through Google Scholar searches, contacting study authors, and connecting with key personnel involved in recovery-focused programmes in LMICs. Reference lists of included full text papers were hand searched for additional sources. Policy documents, conference abstracts, reviews, opinion pieces and commentaries were excluded although they were reviewed to identify published literature.

Stage 3: Literature selection

We followed two independent screening levels for selecting studies for inclusion:(1) title and abstract review; and (2) full-text review. For the first level of review, all citations from the database searches were downloaded into Endnote[29] and duplicates were removed. Thereafter all citations were imported into Rayyan QCRI software[30] which allowed reviewers (FG and ZS) to screen and select titles and abstracts independently according to the inclusion and exclusion criteria. Inter-rater agreement was assessed by calculating Cohen's Kappa. A score of 0.89 was attained, suggesting good interrater reliability.[31]

For peer review only - http://bmjopen.bmj.com/site/about/guidelines.xhtml

One hundred and nine articles (n=109) were selected for full-text review and assessed to determine if they met criteria for study inclusion. Disagreements on study selection were minimal (n=10) and based on interpretations of the outcome, diagnosis or intervention type described in the article. Disagreements were resolved through consensus seeking with a third rater (KS). At this level of screening, inter-rater agreement was calculated as 0.86. Figure 1summarises the literature search and selection process.

Stage 4: Charting the data

All 109 articles selected for full review were read. The two reviewers (FG and ZS) independently extracted and summarised data based on Joanna Briggs Institute data extraction template.[24 25 32] Extracted information included: 1) study title, 2) author, 3) year of study, 4) country, 5) study population, 6) participant characteristics; 7) number of participants, 8) gender distribution, 9) research question and aim, 10) study methodology, 11) study description, 12) data collection methods, 13) data analysis, 14) outcome measures, 15) results, 16) summary of findings; and 17) definitions and conceptual links of recovery. Supplementary file 2 provides information on the questions applied when charting the data. To ensure rigour, there was flexibility regarding emerging categories for data extraction. Ongoing consultation with the team occurred throughout the process.

The Mixed Methods Appraisal Tool (MMAT) version 2018[33] and the Critical Appraisal Skills Programme (CASP) Qualitative checklist[34] were used to assess the quality of the included studies. Additionally, we assessed compliance to ethical standards as part of the quality appraisal process. Indicators included (a) obtaining human research ethics before study commencement, (b) documentation of consent to participate, and (c) management of vulnerability given that the focus was on people living with an SMI.

Stage 5: Collating, summarising and reporting the results

Collated data were extracted and summarised into a descriptive and narrative synthesis of study characteristics that addressed the review questions. Data were summarised to answer the following questions from LMIC settings how recovery is conceptualised; what the descriptors of recovery are; and what influences recovery. FG completed a qualitative data-driven thematic analysis where findings were coded in qualitative software as recommended by Levac et al.[35] using QSR's International NVivo 11 data analysis software.[36] The coding process was recorded in a memo and reviewed with ZS and KS. All authors contributed to reporting the summarised results.

Stage 6: Consultation

For peer review only - http://bmjopen.bmj.com/site/about/guidelines.xhtml

During each stage of the review process the authors consulted with an advisory committee. This advisory committee consists of the research team and key stakeholders including mental health service providers and service users from the public health and non-profit organisation (NPO) sectors. This committee was constituted from the onset of the project and some members are part of a pre-existing group of public mental health specialists. The advisory committee was consulted to gain their opinion on the relevance of the review. They guided access to grey literature, offered their perspectives on the data extraction and charting process, and reviewed the findings. We included the inputs of an advisory committee in this review to enhance its relevance and support collaborative efforts to facilitate consumer participation and public involvement in mental health research.[32 35]

Patient involvement and public involvement

There was no direct patient involvement in this scoping review. Mental health service user representatives were involved in designing the scoping review protocol and were part of the advisory committee.

RESULTS

Search findings

Of the 1530 articles originally identified, 109 articles were selected for full text review. Ten were deemed relevant to the research question. Figure 1 outlines the study selection and exclusion process.

Figure 1: Flow Diagram: PRISMA (preferred Reporting for System Reviews and Meta-Analysis (PRISMA)/ PRISMA Extension for Scoping Review-(PRISMA-ScR)[37]

The results are presented in two sections: (i) a summary of characteristics and quality of the included studies and (ii) the results of the thematic analysis.

Description of included studies

Table 1 summarises the ten studies included in this review. All studies were published between 2014 and 2019 and were conducted in: Turkey (n=2), India (n=2), China (n=1), Swaziland (n=1), Indonesia (n=1), Egypt (n=1), South Africa (n=1) and Vietnam (n=1). According to the World Bank classification system, this covered the range of LMICs. Four studies were conducted in upper-middle income countries, one in a middle-income country and five in lower- middle income countries.

The studies followed different study designs (qualitative: n=8; quantitative n=2). Qualitative studies were guided by the following approaches: grounded theory (n=1), phenomenology

For peer review only - http://bmjopen.bmj.com/site/about/guidelines.xhtml

(n=3), ethnography (n=2) and qualitative descriptive (n=2). In all the qualitative studies, data were collected through semi-structured and unstructured interviews. Five studies focused solely on MHSUs' perspectives[38-42], one described psychiatrists' perspectives[43], and three offered a combination of MHSU and family members perspectives.[44-46] One study reported on multiple stakeholder perspectives by including a combination of MHSUs, family members, community members and healers.[47] Although there was heterogeneity across studies in terms of participants, MHSUs' perspectives were central to conceptualising recovery in most studies.

All participants were adults and the majority of studies focused on a diagnosis of schizophrenia (n=8) while two studies looked at first episode psychosis.[42 45] The age of participants varied between 21-70, participants had been living with an SMI for between one and forty years and recovery was described as occurring over time. [38-41 44 46 47] The participant's gender profile was described in six studies.[38-41 44 46] In studies where the gender profile was reported, more males were included in the service user groups with the exception of one study which had more females.[40] One study included only female service users.[41] In the two quantitative studies, the majority of caregivers were female.

MHSUs in all of the studies were recruited either directly or indirectly from psychiatric services (n=7) or schizophrenia associations (n=2), with inclusion criteria stipulating a DSMIV-R or ICD 10 coding diagnosis. Two studies stipulated that medication adherence was required for participation in the study.[38 39]

For peer review only

Table 1: Studies included in the review (n=)

	Authors (year)	Aim(s)	Country; Setting according to World Bank Classification; Recruitment site	Design and methods	Participants
1	Chen et al.[46] (2018)	To develop an instrument to examine the opinions of consumers and their family members regarding autonomous decision making in family matters	Chengdu, China **Upper- middle income country** Psychiatric Hospital	Quantitative Descriptive	**MHSUs: n=182** Female: n= 85 Male: n=97 **Family members: n= 182** Female: n=103 Male: n=79
2	de Wet et al.[42]	To investigate how MHSUs with first episode psychosis experience their recovery	Cape Town, South Africa **Upper- middle income country** Psychiatric Hospital research Unit	Qualitative Descriptive	**MHSUs: n= 7** Gender not reported
3	Gandhi et al.[40] (2019)	To explore patients' perspectives about factors affecting recovery from schizophrenia	Bengaluru, India **Lower- middle-income country** Psychiatric Hospital (Outpatients department)	Qualitative Grounded theory	**MHSUs: n=18** Males: n= 7 Females: n=11
4	Gopal et al.[44] (2019)	To identify clients and family members' perspectives of what constitutes recovery from schizophrenia in the Indian context and to examine gender differences in recovery indictors	Chennai, India **Lower- middle-income country** Psychiatric Hospital (Outpatients department)	Quantitative Descriptive	**MHSUs n=100** Males: n=55 Females: n=45 **Caregivers: n= 80** Male: n=43 Female: n=37
5	Guner[38] (2014)	To understand patients views and experiences of schizophrenia	Istanbul, Turkey **Upper- middle income country** Schizophrenia Association	Qualitative Descriptive	**MHSUs: n=9** Male: n=8 Female: n=1
6	Humphries et al.[43]	To understand psychiatrists' views on outcomes for people	Danang, Hanoi, HoChi Minh City and Hue, Vietnam	Qualitative Descriptive	**Psychiatrists: n=15**

For peer review only - http://bmjopen.bmj.com/site/about/guidelines.xhtml

	(2015)	with schizophrenia in a developing country	**Middle- income country**<br>Public hospitals		
7	Nxumalo Ngubane et al.[41] (2019)	To explore the experiences and meanings of recovery for Swazi women living with schizophrenia	Swaziland<br>**Lower- middle-income country**<br>Psychiatric Hospital (Outpatients department)	Qualitative Phenomenological	**MHSUs: n=15**<br>Females only
8	Rashed[47] (2015)	To explore the use of spirit-based frameworks to take control over symptoms in their cultural contexts	Dakhla, Western Desert Egypt<br>**Lower- middle-income country**<br>General Hospital with psychiatric service	Qualitative Ethnographic	**MHSUs: n=2**<br>Male: n=1<br>Female: n=1<br>**Family members n= not specified**<br>**Healers**: n=11<br>**Community members**: n=56
9	Soygur[39] (2017)	To identify the factors contributing to recovery, as observed from the perspectives of schizophrenia patients working at a supported employment project	Ankara, Turkey<br>**Upper- middle income country**<br>Schizophrenia Association	Qualitative, Phenomenological;	**MHSUs: n=24**<br>Male: n=15<br>Female: n=9
10	Subandi[45] (2015)	To explore participants' experience of illness and recovery in a Javanese cultural context	Yogyakarta, Java<br>**Lower- middle-income country**<br>Psychiatric Hospital	Qualitative, Ethnographic	**MHSUs: n=7**<br>**Family members**: n= not specified

For peer review only

Table 2: Summary of findings

	Title, Author, Year	Key Findings
1	Family Decision Making and Self-Determination Among Consumers With Schizophrenia in China: Cross-Cultural Implications. Chen et al; 2018	-Autonomy in decision making contributes to the MHSUs functioning during recovery.<br>-In Asian contexts it is accepted practice that family members make decisions on behalf of the MHSUs especially with regard to money management and community and daily living.<br>-Decisions related to psychiatric care are often deferred to health professionals.<br>-The concept of autonomy for MHSUs includes situations where they endorse and agree with decisions made by family members on their behalf.
2	Hearing their voices: The lived experience of recovery from first episode psychosis in schizophrenia in South Africa. de Wet et al; 2015	-Caring for others and being cared for was identified as the biggest contributor to recovery of persons with schizophrenia.<br>Spirituality was seen to build resilience more so than adherence to medication.<br>Understanding the mental illness and managing associated stigma influenced disclosure, especially at work.
3	Perspectives of consumers in India on factors affecting recovery from schizophrenia. Gandhi et al; 2019	-MHSUs in India indicated that recovery was affected by facilitators and barriers related to individual, familial and societal influences.<br>-Holding valued roles in a supportive family structure facilitated the recovery of MHSUs.<br>-MHSUs reported that spirituality and their engagement in prayer and religious rituals anchored them and contributed towards their recovery.<br>-Engaging in activities with others was important for MHSUs as they reported that having meaningful social relationships helped them combat loneliness<br>- Barriers to recovery included side effects of medication, inconsistent treatment approaches and religious beliefs which delayed access to treatment
4	What constitutes recovery in schizophrenia? Client and caregiver perspectives from South India Gopal et al; 2019	-Recovery according to caregivers and MHSUs entailed being symptom free; being able to work; and being independent.<br>-Recovery was equated with no longer needing medication.<br>-Recovery was based on MHSUs subjective feelings and experiences and not on the opinions of family members and health professionals.
5	Illness perception in Turkish schizophrenia patients: A qualitative explorative study. Guner; 2014	-Recovery was variably defined and included a lack of symptoms; being able to work; getting married and having opportunities to became part of community networks.

For peer review only - http://bmjopen.bmj.com/site/about/guidelines.xhtml

		- MHSUs took ownership for their own personal recovery by engaging in meaningful occupations, hobbies and religious practices including prayers.<br>-In Turkey, almost all MHSUs with schizophrenia live with their families<br>For some, having a supportive family facilitated recovery, but recovery was hindered when the family was overprotective.<br>-Recovery was further hindered by difficulties accessing mental health services<br>MHSUs did not disclose their mental illness for fear of stigma
6	Psychiatrists' perceptions of what determines outcomes for people diagnosed with schizophrenia in Vietnam. Humphries et al; 2015	-Access to contemporary treatment was seen as influencing the outcomes for people with schizophrenia. Contemporary treatment is defined as including generic second-generation antipsychotics, staff, facilities and psychosocial interventions.<br>-Psychosocial interventions were not described.<br>-MHSUs feared stigma and this influenced help seeking behaviour.<br>- Psychiatrists reported that in families who hold traditional beliefs, mental illness was attributed to spiritual causes, families access traditional treatment before seeking medical assistance, especially in rural areas.<br>- The extended family system allows family members to share the burden of caring for a MHSU, especially in the absence of formal services<br>-MHSUs were able to access unskilled work in rural parts of Vietnam more readily than MHSUs in urban settings.
7	The experiences and meanings of recovery for Swazi women living with "schizophrenia". Nxumalo Ngubane et al; 2019	--In Swaziland, MHSUs referred to any mental illness as "an illness of the brain"<br>-Schizophrenia is not a known term in Swaziland<br>-Diagnostic labels such as schizophrenia were used by health professionals but not shared with MHSUs.<br>-Families and significant others provided emotional and financial support to MHSUs.<br>-The presence of therapeutic rapport between MHSUs and health professionals contributed to personal recovery.
8	From Powerlessness to Control: Psychosis, spirit possession & recovery in the Western desert of Egypt; Rashed 2015	-Mental illness was contextualised through spirit-based understandings of the illness and its symptoms.<br>-The relationship MHSUs had with the spirit world was used as a means of describing their recovery journeys<br>- Clinical recovery was contested as it suggested that mental illness' can be cured whereas personal recovery was described as an ongoing process that did not require medical intervention.<br>-Personal recovery involved MHSUs gaining control over their symptoms.

For peer review only - http://bmjopen.bmj.com/site/about/guidelines.xhtml

		-MHSUs were positioned as being actively involved in their recovery rather than as passive recipients of services.<br>-Spirituality was foregrounded through the religion of Islam and Quranic healing in the lives of people with schizophrenia.
9	Lessons learned from experiencing Mavi at Café (Blue Horse Café) during six years: A qualitative analysis of factors contributing to recovery from the perspective of schizophrenia patients. Soygur; 2016	-This study was located in a therapeutic community and supported employment setting.<br>- Long term relational support between MHSUs and stakeholders involved in health service delivery was seen as being central to recovery.<br>-Recovery was promoted through a number of factors which were embedded in supportive relationships and unrestrictive environments.<br>-MHSUs reported that having work gave them a sense of purpose, responsibility and motivation which added meaning to their lives.
10	Bangkit: The Processes of Recovery from First Episode Psychosis in Java. Subandi; 2015	-MHSUs viewed their recovery in the cultural context in which they lived<br>-Recovery had different aspects to it and involved medical treatment, family care in the home and integration into natural community settings.<br>-MHSUs reported being guided by spirituality which required their active participation in the performance of Islamic religious practices.

For peer review only

Themes from included studies

Table 2 provides a summary if the key findings of each of the ten included studies. The thematic analysis yielded three themes describing recovery as a personal and complex social process involving healthcare providers, family and community members.[38-44 46 47] These themes, namely (i) recovery is a personal journey occurring along a continuum, (ii) aspects of social relationships supportive of recovery, and (iii) finding meaning and hope through spirituality and religion are elaborated upon below.

1. Recovery is a personal journey occurring along a continuum

The majority of studies included in this review, conceptualised recovery as an ongoing, non-linear process rather than an as an event with a finite end.[38-41 44 45 47] The age at onset of illness and duration of illness was highlighted.[38-41 44 46 47] as being a signal of when the recovery process began. Onset of illness was usually noticed by family members first and they facilitated health-seeking behaviour on behalf of the MHSU.

Some studies[41 43 45-47] defined the starting point for the recovery process as being from the time that people were back in their homes and communities and not from the start of their admission to hospital. This was confirmed by the methodological decisions taken in all of the included studies to not include in-patients or make reference to in-patient services as facilitating recovery. One study described an outpatient service where the programme included psychosocial skills training, family and group therapy sessions and group outings.[38] It is notable that recognising symptoms as cues of relapse was not explored since it appeared that the emphasis was on managing symptoms within the home and community settings after the onset of illness.[40 42 44] This approach occurred after a diagnosis had been established for affected MHSUs.[41 43 45-47]

Since the process of recovery was described from the point of discharge, coping strategies related to managing symptoms of their illness and their re-integration within their families and communities emerged as critical facilitators to recovery. MHSUs needed strategies to facilitate this recovery process. Strategies identified in the studies included the development of self-awareness; being able to self-regulate; spirituality including prayer; attending to basic needs; understanding the mental illness; having purpose and hope for the future; gaining a sense of autonomy; engagement in meaningful occupations; contributing to community; and adopting a positive attitude to living with a mental illness.[38-42 44 46 47] The studies did not describe the development of these coping strategies. It was also noted that learning to self-

For peer review only - http://bmjopen.bmj.com/site/about/guidelines.xhtml

manage facilitated the recovery process.[38 41 45] Key aspects that MHSUs had to learn to self-manage included their temperament, learning to live with and derive meaning from having a mental illness and being hopeful for their future.

There was a lack of consensus across the studies regarding the use of medication as an indicator of recovery.[38-41 43-47] Some studies emphasised treatment adherence as the first steps towards recovery,[38-41 43 46] while others viewed the choice to stop taking medication as an indicator of recovery.[44 45 47] When describing recovery as it relates to the medical model and clinical recovery, studies appeared to focus on the individual and described contemporary treatment as including a combination of access to medication, health personnel, mental health services at health facilities and psychosocial interventions.[40 43] According to Rashed,[47] clinical recovery foregrounds biological aspects, implicitly highlighting a curative approach to the illness, often demonstrated through administering medication. However, in exploring psychosis and spirit possession, Rashed[47] found that recovery was not dependent solely on medication or medical intervention. Instead, recovery was conceptualised as an opportunity to expand personal agency in the way MHSU's respond to their illness, with or without medication.[47]

In the study conducted by Subandi,[45] the recovery process involved different phases of participation and integration across social spheres. Recovery as an active process started as MHSUs gained insight into the social isolation that resulted from their mental illness. Social isolation and withdrawal was viewed as the opposite of recovery as it represented inactivity and passivity.[45 47] Across studies, the effort exerted by MHSUs to expand their social interactions from their immediate physical environment, to engaging with family members and other stakeholders in health and community spaces was as an indicator of recovery.[38-42 44-47]

From the aforementioned it can be seen that the people involved in the recovery process included healthcare providers, family and community members. It is also noteworthy that personal recovery required action in a range of interconnected contexts. MHSUs ability to navigate these different environments indicated what was considered as successful recovery. Indicators of recovery described in the selected articles included: being symptom free, returning to work or supported employment, being independent, getting married, being self-sufficient, making decisions, integrating into social or community networks, and involvement in spiritual and religious activities. Through this, recovery was described as active participation in daily activities. This participation was viewed as an indicator of recovery.[38-47]

For peer review only

2. Aspects of social relationships supportive of recovery

Across included studies, the perspectives and contributions of healthcare providers, family and community members were identified as crucial to the MHSUs recovery process. From these studies it seems that the ways in which these stakeholders engaged with the recovery process potentially facilitated or hindered the recovery journey.[38-47]

Relational support from health professionals was identified as a facilitator of recovery.[38 39 41 42 46] Access to service providers was influenced by the availability of and access to psychiatric treatment.[43 46] Health professionals supported recovery when they communicated clearly, spent time with service users, offered alternative medication options and provided opportunities for engagement in activities.[38 41] On the contrary, the poor continuity of psychiatric care together with limited and brief consultations with a doctor and a focus on access and adherence to medication was seen to limit the recovery process.[38 40 41 47]

Socio-cultural values and variations in explanatory models of mental illness between the MHSUs and health professionals as well as family members influenced views of recovery.[43 46 47] Family members were more likely to seek help from traditional or spiritual healers before seeking contemporary psychiatric treatment.[43 45] The differences between socio-cultural values and explanatory models of mental illness included perceived causes of mental illness, traditional beliefs attributing mental illness to spiritual causes, fear of stigma and community attitudes. For example, discrepancies in the language used to refer to mental illness obscured a common understanding of recovery.[41] MHSUs[41] referred to any mental illness as an "illness of the brain"(p156) while the diagnostic label of schizophrenia was used by health professionals. For one participant the voices of her grandmother was soothing to her but unacceptable to her family and health service providers[41].There was an element of dissonance between how the MHSUs, their families and service providers made sense of the mental illness, with a disconnect occurring around how their symptoms were understood by different parties.[47] When the family, community or health professionals held different understandings and attitudes towards mental illness, recovery was obstructed.

The studies reported that all participants lived with family members or significant others.[38 40-44 47] Although the complexity of large families was acknowledged,[40 43] the extended family supported recovery through sharing the burden of care in the absence of formal health services. The studies highlighted that family and cultural beliefs along with societal values and expectations were used as indicators of recovery[40 45 46]. These indicators were described

For peer review only - http://bmjopen.bmj.com/site/about/guidelines.xhtml

in different ways and included whether the individual could take care of or provide for their family as they recovered.[38 40-44 47]

Families provided emotional and financial support needed by the MHSUs for daily living during their journey of recovery.[38-45 47] While the family assumed responsibility for decision making when the MHSUs were acutely ill, they gave up some of this responsibility as the MHSUs recovered.[38 46 47] The families role as stewards of recovery continued in different ways as they provided flexible access to social networks for MHSUs on an ongoing basis.[38-45 47] Once MHSUs started engaging in spheres such as personal and social functioning, community and daily living and money management; the family's decision making in these spheres decreased.[41 46]

The importance of social support was recognised as a facilitator of recovery. MHSUs accessed support from different sources which included professionals, family members, therapeutic community networks and supported employment.[38 43 47] Peer support from fellow MHSUs was mentioned once as an important facilitator of recovery.[40] Professional support was symptom related, family support involved assisting with tangible aspects such as financial management or with support for community reintegration. Different networks thus facilitated recovery as they provided relational and emotional support to MHSUs.

MHSUs described having social connections and being able to engage in activities alone and with others as key components of personal recovery.[40 44] The MHSUs' social identity and social position in relation to their family, their community and broader society was seen to influence their functioning and social inclusion.[40 44] Stigma was mentioned as a barrier to recovery but it was not elaborated on in the studies.[38 41-43]

In four studies, MHSUs reported that being able to work facilitated access to social networks.[38 39 41 42] For MHSUs, recovery meant becoming part of a social network where they felt valued and which allowed them to meaningfully contribute to their family and community, whether this was in paid employment or not.[38 39 41 42] One study focussed on MHSUs working in a therapeutic community and supported employment setting.[39] The emphasis in this study[39] was on the relational support and social interactions provided to MHSUs via the supported employment project. While employment was important as it gave MHSUs the opportunity to contribute to the financial situation of the family, its role as a facilitator of social support networks was foregrounded. Recovery as a social process which is influenced positively by collaboration between MHSUs and stakeholders through long term relational support and encouragement was emphasised.[39]

For peer review only - http://bmjopen.bmj.com/site/about/guidelines.xhtml

3. Finding meaning and hope through spirituality and religion

Spirituality and religion emerged as resources that the MHSUs and caregivers used for understanding mental illness and the recovery process in eight of the ten studies.[38 40-43 45-47] Five studies[38 40 42 43 46] referred to the significance of spirituality and religion. With regards to religion, Islam was referred to in two studies[45 47] and Christianity in one study.[41] Across these studies, spiritual and religious practices were seen to build resilience and were identified as contributing to recovery when they were equally valued by MHSUs, their families and the community.[38 40-43 45-47]

While in recovery, MHSUs underwent internal spiritual struggles when participating in religious practices outside of their homes. The ways in which this was enacted included directly sharing feelings with God through prayer and not through an intermediary; use of religious rituals to relieve stress; surrendering healing to God's will; resisting spirit possession as an act of faith and personal struggle; and by practically supporting places of worship through engaging in cleaning chores as part of church maintenance.[38 40-42 45 47]

MHSUs relationship with and understanding of the spirit world was seen as an indicator of recovery in one study.[47] Recovery was aided by MHSUs ability to manage the psychotic symptoms of the mental illness while drawing on the philosophical explanations offered by spirituality. In Egypt, spirit possession featured as part of the experience of psychotic phenomenon and psychosis was not necessarily viewed as a medical problem.[47] According to this study, when MHSUs actively engage in accepted individual and communal religious practices, they are acknowledged as positively navigating personal recovery.

MHSUs reported that spirituality and religious practices provided an anchor for MHSUs and their families as it offered hope for recovery through their own efforts while also having faith and trust in a divine power. Engaging in spiritual practices offered MHSUs structured opportunities to relax, relieve their stress and be hopeful about their future.[40 45] Being hopeful about the future required MHSUs to have faith in themselves and their religion as this allowed them to find peace while living with the challenges of having a mental illness.[38 41 42 47] Furthermore, spirituality was viewed as a resource which contributed positively to the recovery process when MHSUs used it to instil hope for the future and as a mechanism for making sense of the mental illness.

For peer review only - http://bmjopen.bmj.com/site/about/guidelines.xhtml

The views held by MHSUs and their family members regarding the influence of spirituality and religious practices on recovery differed from the views held by health professionals.[39-41 43 47] In one study, psychiatrists indicated that religious beliefs and practices could hinder the recovery process particularly where symptoms were attributed to spiritual causes and where traditional treatment was sought before psychiatric treatment.[43] MHSUs viewed professional explanations of mental illness as unhelpful when they discounted the role of spirituality or religion in recovery, especially if they used coercion to support their own ideas of recovery.[41 47] Relying only on medication dispensed by healthcare providers positioned MHSUs as patients and service recipients while adopting a spiritual and cultural interpretation of mental illness, MHSUs were viewed as people who were in control of their illness and who were agents in their own personal recovery process.[45 47] The focus on clinical recovery and the medical interpretation of mental illness minimised the contribution of spirituality to personal recovery.

DISCUSSION

This review contributes to the growing body of research exploring the meaning of recovery from severe mental lillness[15 16 23] by synthesizing qualitative and quantitative research from LMICs. No papers from LMICs fulfilled inclusion criteria before 2014, which suggests that the interest in recovery from SMI has been slower in LMICs than in HICs. The ten articles selected for inclusion were published between 2014-2019 suggesting recent and possibly growing interest in understanding perceptions of recovery in these contexts. While there was heterogeneity in terms of design and setting, all studies endorsed the power of individual narrative accounts of recovery. This review highlighted commonalities but also differences between how studies from HICs and LMICs (1) conceptualise the recovery process; (2) outlines the individual, interpersonal and social factors that facilitate or impede recovery; (3) define indicators of recovery; and (4) view the role of spirituality and religion in the recovery process.

Findings suggest that recovery for MHSUs is conceptualised as a personal, non-linear, and complex process. While this view is echoed in the literature from HICs,[11 16 22 26] the starting point of the process differs between HICs and LMICs. In HICs, MHSUs generally have access to specialised in-patient mental health care and their recovery journeys were thought to begin pre-discharge.[48] In the included studies, none of the MHSUs were in-patients at the time of the study; their recovery journeys were described post discharge. The conceptualisation of recovery as being non-linear in nature incorporates the frequent occurrence of relapses among this population[4] and explains the growing interest in the socio- cultural contexts where recovery takes place,[49] particularly in LMICs.

For peer review only - http://bmjopen.bmj.com/site/about/guidelines.xhtml

Given the variability in socio-cultural contexts, a range of individual, interpersonal and social factors were found to facilitate or impede the process of recovery. First, these publications highlighted the role of explanatory models of mental illness including pathways to care and perceptions regarding the use of medication in the process of recovery. Explanatory models of mental illness[50] provide a framework for understanding SMI and treatment seeking behaviour in a particular context. Systematic reviews have highlighted how in many instances traditional healers are often the first point of contact in many LMICs, including African countries.[51 52] Unlike literature from HICs, views were mixed regarding the role of clinical recovery emphasising medication adherence. While some studies highlighted the role of medication in facilitating recovery,[53-55] others emphasised the additional importance of having an awareness of socio-cultural and explanatory models of illness as these factors influence MHSUs, caregivers and community members and can either facilitate or impede recovery.[52 56 57]

Second, included studies highlighted how recovery requires long term relational and emotional support provided by family and friends. The included studies did not draw a distinction between Eastern and Western societies but the findings indicate that socio-cultural and religious beliefs favour collectivist perspectives which require the involvement of the family throughout MHSUs recovery journeys. The role of the family in MHSUs recovery journeys are emphasised differently in studies from HICs and those from LMICs. In HICs, the deinstitutionalisation process shifted care for MHSUs from hospital to community care. This increased the responsibility placed on family members,[58] but in LMICs access to mental healthcare is a challenge which necessitates that families are involved in the MHSUs recovery journey from the onset, given the limited availability of community care services.[19]

According to several of the included studies,[38 40 41 43-46] the family, including significant others, are responsible for the establishment of supportive living arrangements which involved living with family members, another key facilitator in the process of recovery. These living arrangements seem to reflect norms that are embedded in particular cultures globally and were not viewed as a lack of independence or aspiration. In Western society, gaining independence is seen an indicator of recovery.[11] In contrast, in LMICs, living with supportive family provided MHSUs with opportunities for social cohesion which improved recovery outcomes for MHSUs.[19 59 60] Although supported housing is viewed as a key facilitator of recovery in HICs,[49 61 62] it was not mentioned in any of the included studies described here. Studies in LMICs have examined supported housing for people who are homeless,[63] in the absence of family support. Despite the potentially important role that families can play in the

For peer review only - http://bmjopen.bmj.com/site/about/guidelines.xhtml

recovery of MHSUs, there is limited literature available on how living with family or extended family facilitates recovery in LMICs.[64]

Similar to HICs, accessing opportunities to build social connectedness and community relationships emerged as a facilitator to recovery. Although research indicates that people with SMI have poorer social connections than the general population[22] social relationships allows MHSUs to connect with their family, community and work environments. Employment in the skilled or unskilled labour market, was found to promote personal recovery of people with SMI.[65-69] Although MHSUs have difficulty accessing employment opportunities, in a number of included studies,[39 41 43-46] the ability to work was described as a significant indicator of recovery as well as a means of accessing social networks and supportive relationships which facilitated recovery.

Third, spirituality emerged not only as an important facilitator of the recovery process, but a significant indicator of recovery for MHSUs in LMICs. This is in keeping with a review on religion, spirituality and mental health[70] in HIC and LMICs which concluded that engagement in religious practices was a facilitator of recovery. On an individual level, religion was seen as a source of emotional support,[71] but the influence of religion on family and cultural perspectives and practices, even for ethnic minorities in HICs was not reported on in this review. Included studies in the current review described community religious groups as accessible social networks which provide support to MHSUs and their families.[72] As a facilitator of recovery, spirituality was also seen as a way of finding meaning and hope for MHSUs and their families thought the rituals and social connections embedded in practice.[57 73]

The findings of this scoping review need to be considered in light of some limitations. The review aimed to analyse conceptual and theoretical underpinnings of recovery from SMI in LMICs; as such interventions and intervention outcomes were not focused on. Data synthesis was limited to full text articles available in English and published between January 1993 and December 2019. This practical decision may have led to potentially relevant unpublished papers in other langauges being excluded.

CONCLUSION

This review aimed to examine the literature on how recovery of people with SMI is conceptualised in LMICs, and factors thought to facilitate the recovery process. In this review, we found that understandings of recovery overlapped with those from HICs, but there were important differences. Unlike HIC where independence and autonomy were

For peer review only - http://bmjopen.bmj.com/site/about/guidelines.xhtml

emphasised, social connectedness and interdependence were emphasised as important indicators of recovery in LMICs.[19] In addition, family support played a much larger role in LMICs than in HICs. Peer support services from a range of mental health service providers and MHSUs as a strategy for supporting the recovery process are promoted in HICs.[74] Additionally, spirituality emerged as both a facilitator and an indicator of recovery. In HICs, spirituality is rarely considered in the context of recovery, whereas interdependence with the spiritual realm is viewed as an important indicator and facilitator of recovery in LMICs. These differences highlight the importance of assessing whether tools and measures of recovery from SMI, developed in HICs capture all of the key indicators of recovery that are important for MHSUs living in LMICs. In conclusion, this review highlights the need to expand the recovery concept to be more inclusive of cultural conceptualisations of recovery and illness in LMICs that extend beyond the individual.

Author Contributions

This scoping review involved the intellectual contributions of all the authors. All authors were involved in developing the review question and conceptualising the approach. FG developed and tested the search terms in consultation with a subject librarian. FG, ZS and KS developed the data extraction guideline, and this was revised based on feedback from the other authors. FG and ZS reviewed all the articles for inclusion and discrepancies were referred to KS. RG, KS and BM provided substantial critique and review of the article prior to submission.

Acknowledgements

The two Faculty of Health Science librarians who assisted with the protocol for this review were Mary Shelton and Dilshaad Brey. Our appreciation also goes to Dr Edina Amponsah-Dacosta for her technical insight during the scoping review process.

Funding

This work was supported by the DELTAS Africa Initiative [DEL-15-01]. The DELTAS Africa Initiative is an independent funding scheme of the African Academy of Sciences (AAS)'s Alliance for Accelerating Excellence in Science in Africa (AESA) and supported by the New Partnership for Africa's Development Planning and Coordinating Agency (NEPAD Agency) with funding from the Wellcome Trust [DEL-15-01] and the UK government. The views expressed in this publication are those of the author(s) and not necessarily those of AAS, NEPAD Agency, WellcomeTrust or the UK government. The funder has no role in the design, writing, or submission of the manuscript. This paper is part of a research project

For peer review only - http://bmjopen.bmj.com/site/about/guidelines.xhtml

funded by DELTAS, awarded to FG. BM is funded through the South African Medical Research Council.

Disclosure statement

No potential conflict of interest was reported by the authors

Data availability statement

No additional data available

Paper context

To our knowledge, this is the first scoping review to examine the English, published peer reviewed literature specifically related to recovery from SMI in LMICs. The findings suggest that there is growing interest in understanding recovery outside of HICs contexts.

References

1. Kessler RC, Berglund PA, Bruce ML, et al. The prevalence and correlates of untreated serious mental illness. *Health Serv Res* 2001;36(6 Pt 1):987.
2. Vigo D, Thornicroft G, Atun R. Estimating the true global burden of mental illness. *Lancet Psychiatry* 2016;3(2):171-78. doi: 10.1016/S2215-0366(15)00505-2
3. Eaton J, McCay L, Semrau M, et al. Scale up of services for mental health in low-income and middle-income countries. *Lancet* 2011;378(9802):1592-603. doi: https://doi.org/10.1016/S0140-6736(11)60891-X
4. Petersen I, Lund C, Stein DJ. Optimizing mental health services in low-income and middle-income countries. *Curr Opin Psychiatry* 2011;24(4):318-23. doi: 10.1097/YCO.0b013e3283477afb
5. Sfetcu R, Musat S, Haaramo P, et al. Overview of post-discharge predictors for psychiatric re-hospitalisations: a systematic review of the literature. *BMC Psychiatry* 2017;17(1) doi: 10.1186/s12888-017-1386-z
6. Slade M. Personal recovery and mental illness: A guide for mental health professionals: Cambridge University Press 2009.
7. Razzouk D. Accommodation and Health Costs of Deinstitutionalized People with Mental Illness Living in Residential Services in Brazil. *Pharmacoecon Open* 2019;3(1):31-42. doi: 10.1007/s41669-018-0078-z [published Online First: 2018/05/02]
8. Lund C, Oosthuizen P, Flisher AJ, et al. Pathways to inpatient mental health care among people with schizophrenia spectrum disorders in South Africa. *Psychiatr Serv* 2010;61(3):235-40. doi: doi:10.1176/ps.2010.61.3.235
9. Slade M. Mental illness and well-being: the central importance of positive psychology and recovery approaches. *BMC Health Serv Res* 2010;10:26-26. doi: 10.1186/1472-6963-10-26
10. Myers NL. Culture, stress and recovery from schizophrenia: lessons from the field for global mental health. *Cult Med Psychiatry* 2010;34(3):500-28. doi: 10.1007/s11013-010-9186-7

For peer review only - http://bmjopen.bmj.com/site/about/guidelines.xhtml

11. Deegan PE. Recovery: The lived experience of rehabilitation. *Psychiatr Rehabil J* 1988;11(4):11.
12. Webb L. The recovery model and complex health needs: what health psychology can learn from mental health and substance misuse service provision. *J Health Psychol* 2012;17(5):731-41. doi: 10.1177/1359105311425276
13. Farkas M. The vision of recovery today: what it is and what it means for services. *World Psychiatry* 2007;6(2):68.
14. Anthony WA. Recovery from mental illness: the guiding vision of the mental health service system in the 1990s. *Psychiatr Rehabil J* 1993;16(4):11. doi: 10.1037/h0095655
15. Tew J, Ramon S, Slade M, et al. Social factors and recovery from mental health difficulties: a review of the evidence. *Br J Soc Work* 2012;42(3):443-60. doi: 10.1093/bjsw/bcr076
16. Mathew ST, Nirmala BP, Kommu JVS. Meaning of Recovery Among Persons with Schizophrenia: A Literature Review. *J Psychosoc Rehabil Mental Health* 2018;5(1):73-81. doi: 10.1007/s40737-018-0111-7
17. Kelly M, Lamont S, Brunero S. An Occupational Perspective of the Recovery Journey in Mental Health. *Br J Occup Ther* 2010;73(3):129-35. doi: 10.4276/030802210x12682330090532
18. Cabassa LJ, Nicasio A, Whitley R. Picturing Recovery: A Photovoice Exploration of Recovery Dimensions Among People With Serious Mental Illness. *Psychiatr Serv* 2013;64(9):10.1176/appi.ps.201200503. doi: 10.1176/appi.ps.201200503
19. Aldersey HM, Adeponle AB, Whitley R. Diverse Approaches to Recovery from Severe Mental Illness. The Palgrave Handbook of Sociocultural Perspectives on Global Mental Health2017:109-27.
20. Frost BG, Tirupati S, Johnston S, et al. An Integrated Recovery-oriented Model (IRM) for mental health services: evolution and challenges. *BMC Psychiatry* 2017;17:22. doi: 10.1186/s12888-016-1164-3
21. Lysaker PH, Hamm JA, Hasson-Ohayon I, et al. Promoting recovery from severe mental illness: Implications from research on metacognition and metacognitive reflection and insight therapy. *World J Psychiatry* 2018;8(1):1-11. doi: 10.5498/wjp.v8.i1.1 [published Online First: 2018/03/24]
22. Leamy M, Bird V, Le Boutillier C, et al. Conceptual framework for personal recovery in mental health: systematic review and narrative synthesis. *Br J Psychiatry* 2011;199(6):445-52. doi: 10.1192/bjp.bp.110.083733
23. Llewellyn-Beardsley J, Rennick-Egglestone S, Callard F, et al. Characteristics of mental health recovery narratives: Systematic review and narrative synthesis. *PLoS One* 2019;14:e0214678. doi: 10.1371/journal.pone.0214678
24. Peters MD, Godfrey CM, Khalil H, et al. Guidance for conducting systematic scoping reviews. *Int J Evid Based Healthc* 2015;13(3):141-46.
25. Arksey H, O'Malley L. Scoping studies: towards a methodological framework. *Int J Soc Res* 2005;8(1):19-32. doi: 10.1080/1364557032000119616
26. Slade M, Longden E. Empirical evidence about recovery and mental health. *BMC Psychiatry* 2015;15:285. doi: 10.1186/s12888-015-0678-4
27. Gamieldien F, Galvaan R, Myers B, et al. Exploration of recovery of people living with severe mental illness (SMI) in low-income and middle-income countries (LMIC): a

For peer review only

scoping review protocol. *BMJ Open* 2020;10(2):e032912. doi: 10.1136/bmjopen-2019-032912
28. Tricco AC, Lillie E, Zarin W, et al. Prisma extension for scoping reviews (prisma-scr): Checklist and explanation. *Ann Intern Med* 2018;169(7):467-73. doi: 10.7326/M18-0850
29. Analytics C. EndNote version X8. San Francisco, CA: Clarivate Analytics, 2018.
30. Ouzzani M, Hammady H, Fedorowicz Z, et al. Rayyan—a web and mobile app for systematic reviews. *Systematic reviews* 2016;5(1):210.
31. Blackman NJ-M, Koval JJ. Interval estimation for Cohen's kappa as a measure of agreement. *Stat Med* 2000;19(5):723-41. doi: 10.1002/(sici)1097-0258(20000315)19:5<723::Aid-sim379>3.0.Co;2-a
32. Morgan DL. Pragmatism as a Paradigm for Social Research. *Qual Inq* 2014;20(8):1045-53. doi: 10.1177/1077800413513733
33. Hong QN, Pluye P, Fàbregues S, et al. Mixed methods appraisal tool (MMAT), version 2018. *IC Canadian Intellectual Property Office, Industry Canada* 2018
34. Singh J. Critical appraisal skills programme. *Journal of pharmacology and Pharmacotherapeutics* 2013;4(1):76.
35. Levac D, Colquhoun H, O'Brien KK. Scoping studies: advancing the methodology. *Implement Sci* 2010;5(1):69.
36. Castleberry A. NVivo 10 [software program]. Version 10. QSR International; 2012. *Am J Pharm Educ* 2014;78(1) doi: 10.5688/ajpe78125
37. Moher D, Shamseer L, Clarke M, et al. Preferred reporting items for systematic review and meta-analysis protocols (PRISMA-P) 2015 statement. *Systematic reviews* 2015;4(1):1.
38. Güner P. Illness perception in Turkish schizophrenia patients: A qualitative explorative study. *Arch Psychiatr Nurs* 2014;28(6):405-12. doi: 10.1016/j.apnu.2014.08.016
39. Soygür H, Yüksel Avcioğlu MM, Tekin Eraslan P, et al. Lessons learned from experiencing Mavi at Café (Blue Horse Café) during six years: A qualitative analysis of factors contributing to recovery from the perspective of schizophrenia patients. *Turkish Journal of Psychiatry* 2017;28(2):1-7.
40. Gandhi S, Jose D, Desai G. Perspectives of consumers in India on factors affecting recovery from schizophrenia. *Int J Soc Psychiatry* 2019:20764019877425. doi: 10.1177/0020764019877425 [published Online First: 2019/09/24]
41. Nxumalo Ngubane S, McAndrew S, Collier E. The experiences and meanings of recovery for Swazi women living with "schizophrenia". *J Psychiatr Ment Health Nurs* 2019;26(5-6):153-62. doi: 10.1111/jpm.12520 [published Online First: 2019/05/03]
42. de Wet A, Swartz L, Chiliza B. Hearing their voices: The lived experience of recovery from first-episode psychosis in schizophrenia in South Africa. *Int J Soc Psychiatry* 2015;61(1):27-32. doi: 10.1177/0020764014535753 [published Online First: 2014/05/31]
43. Humphries SH, King RJ, Dunne MP, et al. Psychiatrists' perceptions of what determines outcomes for people diagnosed with schizophrenia in Vietnam. *ASEAN Journal of Psychiatry* 2015;16(2):181-92.
44. Gopal S, Mohan G, John S, et al. What constitutes recovery in schizophrenia? Client and caregiver perspectives from South India. *Int J Soc Psychiatry* 2019;0(0):0020764019886339. doi: 10.1177/0020764019886339

For peer review only - http://bmjopen.bmj.com/site/about/guidelines.xhtml

45. Subandi MA. Bangkit: The Processes of Recovery from First Episode Psychosis in Java. *Cult Med Psychiatry* 2015;39(4):597-613. doi: 10.1007/s11013-015-9427-x [published Online First: 2015/01/21]
46. Chen Y, Lam CS, Deng H, et al. Family Decision Making and Self-Determination Among Consumers With Schizophrenia in China: Cross-Cultural Implications. *Psychiatr Serv* 2018;69(4):462-68. doi: 10.1176/appi.ps.201500255
47. Rashed MA. From Powerlessness to Control: Psychosis, spirit possession & recovery in the Western desert of Egypt. *Health, Culture & Society* 2015;8(2):10-26. doi: 10.5195/hcs.2015.194
48. van Weeghel J, van Zelst C, Boertien D, et al. Conceptualizations, assessments, and implications of personal recovery in mental illness: A scoping review of systematic reviews and meta-analyses. *Psychiatr Rehabil J* 2019;42(2):169.
49. Doroud N, Fossey E, Fortune T. Place for being, doing, becoming and belonging: A meta-synthesis exploring the role of place in mental health recovery. *Health Place* 2018;52:110-20. doi: 10.1016/j.healthplace.2018.05.008
50. Makanjuola V, Esan Y, Oladeji B, et al. Explanatory model of psychosis: impact on perception of self-stigma by patients in three sub-saharan African cities. *Soc Psychiatry Psychiatr Epidemiol* 2016;51(12):1645-54. doi: 10.1007/s00127-016-1274-8
51. Burns JK, Tomita A. Traditional and religious healers in the pathway to care for people with mental disorders in Africa: a systematic review and meta-analysis. *Soc Psychiatry Psychiatr Epidemiol* 2015;50(6):867-77. doi: 10.1007/s00127-014-0989-7
52. Green B, Colucci E. Traditional healers' and biomedical practitioners' perceptions of collaborative mental healthcare in low- and middle-income countries: A systematic review. *Transcult Psychiatry* 2020;57(1):94-107. doi: 10.1177/1363461519894396 [published Online First: 2020/01/16]
53. Winsper C, Crawford-Docherty A, Weich S, et al. How do recovery-oriented interventions contribute to personal mental health recovery? A systematic review and logic model. *Clin Psychol Rev* 2020;76:101815.
54. Le Boutillier C, Chevalier A, Lawrence V, et al. Staff understanding of recovery-orientated mental health practice: a systematic review and narrative synthesis. *Implement Sci* 2015;10:87. doi: 10.1186/s13012-015-0275-4
55. Chester P, Ehrlich C, Warburton L, et al. What is the work of recovery oriented practice? A systematic literature review. *International journal of mental health nursing* 2016;25(4):270-85.
56. Thornicroft G, Deb T, Henderson C. Community mental health care worldwide: current status and further developments. *World Psychiatry* 2016;15(3):276-86. doi: 10.1002/wps.20349
57. Bhikha AG, Farooq S, Chaudhry N, et al. A systematic review of explanatory models of illness for psychosis in developing countries. *Int Rev Psychiatry* 2012;24(5):450-62.
58. Ward-Griffin C, Schofield R, Vos S, et al. Canadian families caring for members with mental illness: A vicious cycle. *J Fam Nurs* 2005;11(2):140-61.
59. Ran MS, Weng X, Liu YJ, et al. Changes in treatment status of patients with severe mental illness in rural China, 1994-2015. *BJPsych Open* 2019;5(2):e31. doi: 10.1192/bjo.2019.13 [published Online First: 2019/05/10]
60. Chan SKW, Hui CLM, Chang WC, et al. Ten-year follow up of patients with first-episode schizophrenia spectrum disorder from an early intervention service: Predictors of

For peer review only

clinical remission and functional recovery. *Schizophr Res* 2019;204:65-71. doi: 10.1016/j.schres.2018.08.022
61. McPherson P, Krotofil J, Killaspy H. Mental health supported accommodation services: A systematic review of mental health and psychosocial outcomes. *BMC Psychiatry* 2018;18(1) doi: 10.1186/s12888-018-1725-8
62. Carpenter-Song E, Hipolito MMS, Whitley R. 'Right here is an oasis': How 'recovery communities' contribute to recovery for people with serious mental illnesses. *Psychiatric Rehabilitation Journal* 2012;35(6):435-40. doi: 10.1037/h0094576
63. Padmakar A, De Wit EE, Mary S, et al. Supported Housing as a recovery option for long-stay patients with severe mental illness in a psychiatric hospital in South India: Learning from an innovative de-hospitalization process. *PLoS One* 2020;15(4):e0230074. doi: 10.1371/journal.pone.0230074
64. Gopal S, Henderson AR. Trans-cultural study of recovery from severe enduring mental illness in Chennai, India and Perth, Western Australia. *J Psychosoc Rehabil Mental Health* 2015;2(1):51-57.
65. Young DK, Cheng D, Ng P. Predictors of Personal Recovery of People with Severe Mental Illness in a Chinese Society: a Cross-Sectional Study with a Random Sample. *International Journal of Mental Health and Addiction* 2019 doi: 10.1007/s11469-019-00134-w
66. Thomas TL, Prasad Muliyala K, Jayarajan D, et al. Vocational challenges in severe mental illness: A qualitative study in persons with professional degrees. *Asian J Psychiatr* 2019;42:48-54. doi: 10.1016/j.ajp.2019.03.011 [published Online First: 2019/04/07]
67. Doroud N, Fossey E, Fortune T. Recovery as an occupational journey: A scoping review exploring the links between occupational engagement and recovery for people with enduring mental health issues. *Aust Occup Ther J* 2015;62(6):378-92. doi: 10.1111/1440-1630.12238
68. Lund C, Brooke-Sumner C, Baingana F, et al. Social determinants of mental disorders and the Sustainable Development Goals: a systematic review of reviews. *Lancet Psychiatry* 2018;5(4):357-69. doi: 10.1016/s2215-0366(18)30060-9
69. Gamieldien F, van Niekerk L. Street vending in South Africa: An entrepreneurial occupation. *South African Journal of Occupational Therapy* 2017;47(1):24-29. doi: 10.17159/2310-3833/2017/vol47n1a5.
70. Borthwick CHDKA. The relevance of moral treatment to contemporary mental health care. *Journal of Mental Health* 2001;10(4):427-39. doi: 10.1080/09638230124277
71. Koenig HG. Research on Religion, Spirituality, and Mental Health: A Review. *The Canadian Journal of Psychiatry* 2009;54(5):283-91. doi: 10.1177/070674370905400502
72. Griffith JL, Myers N, Compton MT. How Can Community Religious Groups Aid Recovery for Individuals with Psychotic Illnesses? *Community Ment Health J* 2016;52(7):775-80. doi: 10.1007/s10597-015-9974-1 [published Online First: 2015/12/30]
73. Abdaleati NS, Mohd Zaharim N, Mydin YO. Religiousness and Mental Health: Systematic Review Study. *Journal of Religion and Health* 2016;55(6):1929-37. doi: 10.1007/s10943-014-9896-1
74. Lloyd-Evans B, Mayo-Wilson E, Harrison B, et al. A systematic review and meta-analysis of randomised controlled trials of peer support for people with severe mental illness. *BMC Psychiatry* 2014;14:39-39. doi: 10.1186/1471-244X-14-39

For peer review only - http://bmjopen.bmj.com/site/about/guidelines.xhtml

For peer review only

For peer review only - http://bmjopen.bmj.com/site/about/guidelines.xhtml

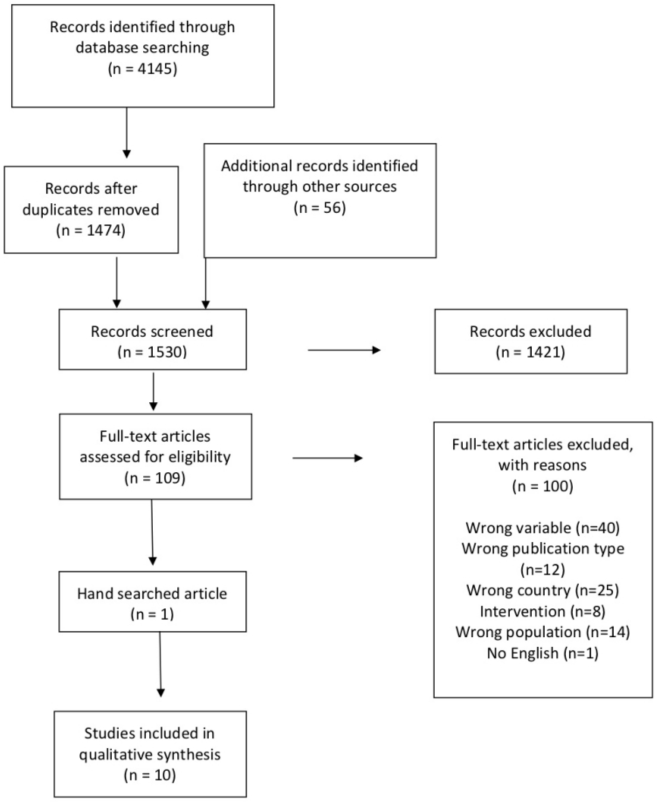

Flow Diagram: PRISMA (preferred Reporting for System Reviews and Meta- Analysis (PRISMA)/ PRISMA Extension for Scoping Review-(PRISMA-ScR

209x297mm (150 x 150 DPI)

Open access Protocol

BMJ Open

Exploration of recovery of people living with severe mental illness (SMI) in low-income and middle-income countries (LMIC): a scoping review protocol

Fadia Gamieldien ,[1,2] Roshan Galvaan,[2] Bronwyn Myers,[3,4] Katherine Sorsdahl[1]

To cite: Gamieldien F, Galvaan R, Myers B, *et al*. Exploration of recovery of people living with severe mental illness (SMI) in low-income and middle-income countries (LMIC): a scoping review protocol. *BMJ Open* 2020;**10**:e032912. doi:10.1136/bmjopen-2019-032912

► Prepublication history and additional material for this paper are available online. To view these files, please visit the journal online (http://dx.doi.org/10.1136/bmjopen-2019-032912).

Received 11 July 2019
Revised 24 December 2019
Accepted 06 January 2020

Check for updates

© Author(s) (or their employer(s)) 2020. Re-use permitted under CC BY. Published by BMJ.

[1]Alan J. Flisher Centre for Public Mental Health, Department of Psychiatry and Mental Health, University of Cape Town, Cape Town, South Africa
[2]Division of Occupational Therapy, Department of Health and Rehabilitation Sciences, University of Cape Town, Cape Town, South Africa
[3]Alcohol, Tobacco and Other Drug Research Unit, South African Medical Research Council, Cape Town, South Africa
[4]Division of Addiction Psychiatry, Department of Psychiatry and Mental Health, University of Cape Town, Cape Town, South Africa

Correspondence to
Fadia Gamieldien;
fadia.gamieldien@uct.ac.za

ABSTRACT

Introduction The construct of recovery was conceptualised in high-income countries and its applicability in low-income and middle- income countries is underexplored. A scoping review is proposed to synthesise knowledge, review conceptual overlap and map key elements of recovery from severe mental illness in low-income and middle-income countries. We aim to appraise the literature so as to inform future recovery-oriented services that consider the cultural and contextual influences on recovery from severe mental illness.

Methods and analysis The following electronic databases: MEDLINE via PubMed, SCOPUS (which included contents of Embase), PsycINFO, CINAHL, Africa-Wide Information, PsycARTICLES, Health source: Nursing/Academic Edition, Academic Search Premier and SocINDEX all via the EBSCOHOST platform, the Latin American and Caribbean Health Sciences Literature, the Cochrane Centre Register of Controlled Trials) and grey literature sources will be searched between May and December 2019. Eligible studies will be independently screened for inclusion and exclusion by two reviewers using a checklist developed for this purpose. Studies published between January 1993 and November 2019 that focus on recovery from severe mental illness in a low-income and middle-income country will be included. Findings will be compared and discrepancies will be discussed. Unresolved discrepancies will be referred to a third reviewer. All bibliographic data and study characteristics will be extracted and thematically analysed using a tool developed through an iterative process by the research team. Indicators will be classified according to a predefined conceptual framework and categorised and described using qualitative content analysis.

Ethics and dissemination The review aims to synthesise information from available publications, hence it does not require ethical approval. The results will be disseminated through publications, conference presentations and future workshops with stakeholders involved within the recovery paradigm of mental health policy and practice. The scoping review title is registered with the Joanna Briggs Institute.

Strengths and limitations of this study

- This scoping review allows for the systematic synthesis of knowledge on recovery from severe mental illness in low-income and middle-income countries (LMICs) in a rigorous and methodological manner.
- This review will highlight the diversity of severe mental illness since there is no agreed on criteria for including mental illnesses into this category.
- Knowledge gaps related to recovery as a multidimensional construct in LMICs will be identified.
- Only conceptual and theoretical underpinnings of recovery as a construct in LMIC will be discussed without a focus on intervention outcomes.
- Data synthesis will be limited to full-text articles available in English only and published between January 1993 and November 2019. Grey literature and unpublished studies in Latin America will be affected by the decision to exclude articles not available in English.

INTRODUCTION

Mental, neurological and substance use (MNS) disorders contribute significantly to the global burden of disease. Whiteford *et al*[1] report that MNS disorders account for 10% of disability-adjusted life years (DALYs). However, it has been argued that the true global burden of mental health problems may be underestimated by more than one-third and that DALYs are closer to 13%.[2] The lifetime prevalence of severe mental illness (SMIs) ranges between 1% and 4%.[3] Although this is relatively low in comparison to common mental disorders, SMIs are more disabling and require complex, long-term interventions for the person and their family.[2] Given the move towards the deinstitutionalisation of people with psychiatric disorders, shorter hospital stays are resulting in premature discharge rates and repeated relapse, creating a revolving door phenomenon.[4] These frequent hospital readmissions are costly to the healthcare system.[5]

In low-income and middle-income countries (LMICs), many people living with an SMI are not detected and as a result do not receive the treatment they need.[3 6 7] In a service

For peer review only - http://bmjopen.bmj.com/site/about/guidelines.xhtml

For peer review only

BMJ Open: first published as 10.1136/bmjopen-2019-032912 on 3 February 2020. Downloaded from http://bmjopen.bmj.com/ on February 4, 2020 at University of Cape Town Libraries. Protected by copyright.

utilisation review of WHO member countries, it was found that the lifetime prevalence of mental disorders among adults was between 12% and 49%.[6] The review found the treatment gap to be wide across the psychiatric disorders examined. The treatment gap for schizophrenia and non-affective psychosis was 32%. The gap for other disorders was: major depressive disorder, 56%; bipolar disorder, 50%; and alcohol abuse and dependence, 78%.[6] While present in all countries, this treatment gap is greatest in LMICs where there is a need to scale up mental health services.[8]

A systematic review on the scaling up of mental health services in LMICs found that in many countries the majority of resources for mental healthcare are rendered on an inpatient or outpatient basis at large psychiatric hospitals, with little provision of services at primary healthcare facilities or via integration between hospitals and the community.[8] Although there are some nascent programmes to provide services for SMI at primary care facilities, these are generally limited because of a poor integration of mental health into primary healthcare. Additionally, while the lack of coordination between sectors providing mental health services has seen some countries, such as India, have made progress in adopting the District Mental Health Programme, the treatment gap continues in rural areas.[9 10]

Despite the drive for deinstitutionalisation and for the rendering of services at decentralised locations, in many countries most of the mental healthcare budget continues to be allocated to large psychiatric hospitals. In these hospitals, the treatment approach is primarily through medication to alleviate acute clinical symptoms, giving little to no attention to the psychosocial needs of service users.[4 8 11] Unfortunately, the primary focus on clinical recovery and the limited availability of interventions that focus on rehabilitation and personal recovery further exacerbates the revolving door phenomenon and ongoing treatment gap.[4 12]

Recovery-oriented approaches offer an extension to medical models of intervention, as they are not solely focused on symptomatic improvement as a marker of clinical recovery.[13] Personal recovery for people with SMI is conceptually distinct from the present medical definition of recovery, which is clinical in nature and mostly values symptom remission.[14 15] Personal recovery proposes that a person develops new meaning and purpose in their life over the longitudinal course of their illness.[13 16] Psychiatric rehabilitation has highlighted the need to move beyond treating the symptoms of the illness, to treating its functional and disabling consequences as well.[17] Deegan, a mental health professional and person living with schizophrenia, suggested that mental health service users do not 'get rehabilitated' by others, but instead they are active participants in their journey to recover new and valued personal meaning and purpose.[18]

The recovery model was initiated in high-income countries (HICs) with the mental health consumer and survivor movement at a time when having a diagnosis of SMI was highly stigmatised and the prognosis was poor.[18] The idea that people with an SMI cannot recover was challenged by people living with schizophrenia who were leading meaningful lives in their communities after deinstitutionalisation, highlighting that recovery was not dependant on long-term psychiatric treatment.[19 20]For a person living with an SMI, receiving a diagnosis and psychiatric treatment focused on clinical recovery is a necessary step towards recovery, but personal recovery is also critical given the cyclical, non-linear, long-term course of the illness. In Anthony's seminal work,[17] he defined recovery as:

> a deeply personal, unique process of changing ones' attitude, values, feelings, goals, skills and/or roles. It is a way of living a satisfying, hopeful and contributing life even within the limitations caused by illness

Research exploring the meaning of recovery among people living with SMI[21–23] found that recovery means different things to different people, supporting a view that there is no definition of recovery that applies to everyone. A literature review conducted in 2018 explored the meaning of recovery among people living with schizophrenia.[21] This review included 17 studies explaining the subjective meaning of recovery. A majority of the included studies were qualitative, with a few taking a quantitative approach to exploring the meaning of recovery. Of the 17 papers, only four studies were from LMICs (India (n=2), China and South Africa). The results revealed that recovery is complex, non-linear and perceived as both a process and an outcome. The process was viewed as being individual and it was recommended that more consumer perspectives to inform recovery-oriented interventions, which are aligned to individuals' personal goals and aspirations were needed. The complexity embedded in viewing recovery as a process and an outcome with multidimensional indicators has been documented by others.[24–26] Further to this, it was recommended that clinicians, caregivers and researchers explore more qualitative and personal narratives of recovery, the tools needed to measure recovery and the development of community-based recovery- oriented services.[21]

The aforementioned review was limited to recovery in people living with schizophrenia and only included four studies from LMICs. There is current debate around whether people with SMI in LMICs have a better prognosis and a call to re-examine the realities of living with an SMI amidst social, cultural and economic changes in LMICs.[27 28]

Recovery has been viewed as a process or an outcome, with current debates and research seeking to offer conceptual clarity.[12 29] A systematic review conducted by Leamy *et al*[12] led to a proposed conceptual framework for personal recovery in mental illness. The review focused on 97 papers from 13 HICs and identified characteristics, processes and stages of recovery. While the review included individuals from black and minority ethnic groups, it did not include any LMICs.[12] The CHIME

 Gamieldien F, *et al*. *BMJ Open* 2020;**10**:e032912. doi:10.1136/bmjopen-2019-032912

For peer review only - http://bmjopen.bmj.com/site/about/guidelines.xhtml

BMJ Open: first published as 10.1136/bmjopen-2019-032912 on 3 February 2020. Downloaded from http://bmjopen.bmj.com/ on February 4, 2020 at University of Cape Town Libraries. Protected by copyright.

framework emerged from this review process. This frameworkconceptualises personal recovery to incorporate the following concepts: **c**onnectedness, **h**ope and **o**ptimism about the future, **i**dentity, **m**eaning in life and **e**mpowerment.[12] This highlights that literature on personal recovery is dominated by research conducted in HICs and that there is a gap in understanding recovery from culturally diverse groups.[30]

Research seeking to understand recovery as a process and an outcome is ongoing.[31] The complexity of the concept and the range of factors impacting recovery has been the topic of recent studies. One such focal area has been on the relationship between homelessness among people living with SMIs and the contribution of stable housing towards recovery.[32 33] Additionally, a recent exploration of metacognition and recovery from mental illness challenged conventional treatment to embrace the notion that recovery is unique, individualised and self-directed and that service users need to be active participants in their recovery processes.[26] A call has been made for mental health service users to be partners in conceptualising recovery as a concept and treatment orientation in order to integrate the notion of recovery into mainstream psychiatry.[31]

A scoping review of systematic reviews and meta-analyses conducted by van Weeghel *et al*[29] mapped the concept of recovery and its assessment. It found that recovery is a personal process that is dynamic and evolves over time.[29] Although 25 studies were included in the review, only one study was conducted by authors from India. Conducting a scoping review focusing specifically on studies conducted in LMICs will contribute to the literature on recovery in these specific contexts and will be useful for the following reasons. First, little attention has been given to describing recovery as a personal and individual journey from LMIC perspectives. As recovery for the person with an SMI takes place in a social context through a range of relationships, more understanding about how contexts can help or hinder recovery is needed.[34]

Second, individual meaning making within recovery is influenced by social, political, economic and human rights factors but further research is needed to understand the diversity of recovery narratives from the perspectives of different population groups.[35] A systematic review and narrative analysis synthesising descriptions and models of personal recovery was undertaken to inform the development of a conceptual framework to guide recovery oriented research and practice.[12] Studies conducted in HIC specifically the USA featured predominantly in the review which included 97 papers. The review culminated in the emergence of a conceptual framework to describe the characteristics of recovery, the processes of recovery and the stages of recovery. The authors reported a difference in studies which included ethnic minorities and where more emphasis was placed on stigma, spirituality, culture and the collectivist nature of recovery. They acknowledged that this area is underexplored.[12]

Third, in many African cultures, an individual's personhood is manifested through their agency in relation to spirituality, society and self.[36] This may impact on the person and how they experience mental illness and recovery. Understanding cultural concepts and the dynamics of interconnectedness means recognising that personhood in LMICs is weaved into how people experience health, illness and recovery.[36] Considering the person as a spiritual and social being provides opportunities to collaborate with caregivers and communities in developing appropriate interventions.[36] In countries that are spiritually diverse, multicultural and home to people of different religious backgrounds, it is important to contextualise mental illness and explore health professionals own perspectives on culture, psychiatry and mental health and where mental illness must be contextualised.[37 38] Given the reasons cited previously, further exploration into the recovery journeys of people living with SMI in LMICs and how they view the dimensions of recovery is warranted.[21]

A scoping review is proposed as a means of synthesising knowledge on a broadly defined topic and systematically mapping key concepts, theories, evidence and research gaps in the topic area, while still being rigorous and methodological in its approach.[39 40] While systematic reviews and meta-analyses limit their parameters to research trials and quantitative data synthesis, scoping reviews adopt a broader approach to the narrative integration of evidence.[41] We aim to appraise the definitions of recovery and its current utilisation for people with SMI in LMICs. The review findings could be used to inform future practice applications, which consider the cultural and contextual factors impacting on recovery[15] from SMI outside of HICs. This scoping review title is registered with the Joanna Briggs Institute (JBI) although JBI and the International Prospective Register of Systematic Reviews (PROSPERO) do not provide registry numbers for scoping reviews at this time.[39]

METHODS

A scoping review was deemed as the most suitable method to map existing research on recovery from SMI in LMICs because it allows for the topic to be located, examined, summarised and be presented rapidly and systematically. Additionally, it could serve as a precursor to a systematic review.[42] The proposed review will be conducted using the Arksey and O'Malley,[40] scoping review methodology and will be reported on using the Preferred Reporting Items for Systematic Reviews and Meta-Analyses guidelines[43] with an understanding that there is much development as scoping reviews gain popularity especially in health-related topics.[39 44]

Arksey and O'Malley[40] offer a methodological framework for conducting a scoping review. Their approach describes six stages: (1) identification of the research question, (2) identification of relevant studies, (3) selection of studies, (4) charting the data, (5) collating, summarising

For peer review only - http://bmjopen.bmj.com/site/about/guidelines.xhtml

BMJ Open: first published as 10.1136/bmjopen-2019-032912 on 3 February 2020. Downloaded from http://bmjopen.bmj.com/ on February 4, 2020 at University of Cape Town Libraries. Protected by copyright.

and reporting of results and (6) consultation with stakeholders.[40] The last stage is optional according to Arksey and O'Malley,[40] but others have deemed it a necessary step to aide in methodological rigour.[45] Recommendations to enhance the methodology at each stage will be incorporated into this review.[39 43 45]

Stage 1: identifying the research question

In order to guide the search strategy an iterative process of discussion among authors occurred in order to develop and refine the research question. The question needed to be clear enough to inform the subsequent stages while still reflecting the scope of inquiry.[45] Given that scoping studies are focused on summarising the breadth of evidence on a given topic the authors refined the exploratory question to ask the following:

What is known about recovery from SMIs in LMICs?

This broad question was developed to allow for a comprehensive review of the literature in order to review and appraise the definition of recovery and how it is understood in mental health research and practice in LMICs. While many have written about this in HICs,[23 46] there is a dearth of such information in LMICs .

Stage 2: identifying relevant studies

Given the aim of the study is to identify primary published and unpublished studies which will answer the research question, a comprehensive search strategy was developed to aid this. Two health sciences librarians assisted the first author in the iterative process of developing a search strategy including inclusion and exclusion criteria. Decisions were made about time frames, language, search terms and search strategies. Trial searches were run to assess whether relevant literature could be identified using the proposed strategy. A discussion of the identified databases, search strategy and selection criteria follow.

Databases

Electronic databases will be searched to identify studies published between January 1993 and November 2019. This period was selected as it covers roughly 25 years in the recovery movement. Personal recovery is a construct that has already been defined by Anthony,[17] hence the authors were looking specifically for literature on this construct. Terms used in other disciplines that could also mean recovery were not included as the focus of the search is on recovery as explicitly defined by the recovery movement. Furthermore, recovery knowledge and attitudes among health professionals will be included if reported in the selected studies.

An expert librarian assisted the first author in identifying information sources that are relevant to the research question. The following databases were selected: MEDLINE via PubMed, SCOPUS (which included contents of Embase), PsycINFO, CINAHL, Africa-Wide Information, PsycARTICLES, Health source: Nursing/Academic Edition, Academic Search Premier and SocINDEX all via the EBSCOHOST platform. Additionally, the Latin American and Caribbean Health Sciences Literature, the Cochrane Centre Register of Controlled Trials. Grey literature sources will be identified to expand the search and enhance the data sources. Additional sources will be located by means of handsearching reference lists of relevant papers, contacting study authors, searching trial registers and contacting key personnel involved in recovery-focused programmes in LMICs. In this way, peer-reviewed and grey literature from the biomedical sciences, allied health sciences, social sciences and other disciplinary fields will be included.

Search strategy

Two librarians and the first author developed the eligibility criteria for the scoping review. They identified various definitions of SMIs as well as recovery to include in this search strategy. A list of preliminary search terms and filtering methods was developed. The main filtering methods related to the date range of 25 years (January 1993–November 2019). This period was extended so that new publications would not be missed. The search strategy was refined to include Medical Subject Headings (MeSH terms), filters and Boolean operations to comply with searches across different databases. A search strategy for the PubMed database is included in online supplementary file 1. After the initial search, key words and index terms used across the databases were identified and plotted on a table as indicated below (table 1).

There are no universally accepted guidelines to operationalise the concept of recovery and there is no measure of a gold standard of recovery.[47] The CHIME framework is one proposed guide for the development of recovery measures and identifying recovery outcomes for consideration in clinical practice, but this is limited to HICs. In this review, the key concept is personal recovery as defined by the seminal work of Anthony.[17] In the search strategy, the concept of recovery will be explored through the MeSH terms: Psychiatric Rehabilitation; Mental Health Recovery and Recovery of Function as well as through the free-text terms: Recovery OR recover OR psychosocial rehabilitation OR mental health rehabilitation OR psychiatric rehabilitation.

Selection criteria

Articles will be included in the review if they meet the following inclusion and exclusion criteria (table 2).

Stage 3: study selection

The scoping review guideline for identifying and selecting studies informs article selection and data extraction.[40] Two screening levels are proposed: a title and abstract review and a full-text review. For the first level of screening, two authors (FG and KS) will independently screen the title and abstracts of all retrieved citations against a few select inclusion criteria. Any articles that seem relevant by either or both reviewers will be included in the full-text review. Next, these two reviewers will independently screen the full-text articles to assess whether they meet

For peer review only - http://bmjopen.bmj.com/site/about/guidelines.xhtml

BMJ Open: first published as 10.1136/bmjopen-2019-032912 on 3 February 2020. Downloaded from http://bmjopen.bmj.com/ on February 4, 2020 at University of Cape Town Libraries. Protected by copyright.

Table 1 Search strategy terms

Keyword	Alternative
Recovery	Recovery OR recover OR psychosocial rehabilitation OR mental health rehabilitation OR psychiatric rehabilitation OR mental health recovery OR Recovery of function OR Quality of life
Severe mental illness	Severe mental illness OR bipolar OR delusional disorder OR delusional disorders OR major depressive disorder OR major depressive disorders OR schizophrenia OR manic OR manic-depressive OR paranoid disorder OR paranoid disorders OR psychoses OR psychosis OR psychotic disorder OR psychotic disorders OR schizoaffective disorder OR schizoaffective disorders OR Schizophreniform OR serious mental disorder OR serious mental disorders Schizophrenia Spectrum and Other Psychotic Disorders<br>Bipolar and Related Disorders<br>Depressive Disorder
Low-income and middle- income countries	Deprived Countries OR Deprived Population OR Deprived Populations OR Developing Countries OR Developing Country OR Developing Economies OR Developing Economy OR Developing Nation OR Developing Nations OR Developing Population OR Developing Populations OR Developing World OR LAMI Countries OR LAMI Country OR Less Developed Countries OR Less Developed Country OR Less Developed Economies OR Less Developed Nation OR Less Developed Nations OR Less Developed World OR Lesser Developed Countries OR Lesser Developed Nations OR LMIC OR LMICS OR Low GDP OR Low GNP OR Low Gross Domestic OR Low Gross National OR Low Income Countries OR Low Income Country OR Low Income Economies OR Low Income Economy OR Low Income Nations OR Low Income Population OR Low Income Populations OR Lower GDP OR lower gross domestic OR Lower Income Countries OR Lower Income Country OR Lower Income Nations OR Lower Income Population OR Lower Income Populations OR Middle Income Countries OR Middle Income Country OR Middle Income Economies OR Middle Income Nation OR Middle Income Nations OR Middle Income Population OR Middle Income Populations OR Poor Countries OR Poor Country OR Poor Economies OR Poor Economy OR Poor Nation OR Poor Nations OR Poor Population OR Poor Populations OR poor world OR Poorer Countries OR Poorer Economies OR Poorer Economy OR Poorer Nations OR Poorer Population OR Poorer Populations OR Third World OR Transitional Countries OR Transitional Country OR Transitional Economies OR Transitional Economy OR Under Developed Countries OR Under Developed Country OR under developed nations OR Under Developed World OR Under Served Population OR Under Served Populations OR Underdeveloped Countries OR Underdeveloped Country OR underdeveloped economies OR underdeveloped nations OR underdeveloped population OR Underdeveloped World OR Underserved Countries OR Underserved Nations OR Underserved Population OR Underserved Populations<br>OR<br>Afghanistan OR Albania OR Algeria OR American Samoa OR Angola OR Armenia OR Azerbaijan OR Bangladesh OR Belarus OR Byelarus OR Belorussia OR Belize OR Benin OR Bhutan OR Bolivia OR Bosnia OR Botswana OR Brazil OR Bulgaria OR Burma OR Burkina Faso OR Burundi OR Cabo Verde OR Cape Verde OR Cambodia OR Cameroon OR Central African Republic OR Chad OR China OR Colombia OR Comoros OR Comores OR Comoro OR Congo OR Costa Rica OR Côte d'Ivoire OR Cuba OR Djibouti OR Dominica OR Dominican Republic OR Ecuador OR Egypt OR El Salvador OR Equatorial Guinea OR Eritrea OR Ethiopia OR Fiji OR Gabon OR Gambia OR Gaza OR Georgia OR Georgia Republic OR Ghana OR Grenada OR Grenadines OR Guatemala OR Guinea OR Guinea- Bissau OR Guyana OR Haiti OR Herzegovina OR Hercegovina OR Honduras OR India OR Indonesia OR Iran OR Iraq OR Jamaica OR Jordan OR Kazakhstan OR Kenya OR Kiribati OR Democratic People's Republic of Korea OR Kosovo OR Kyrgyz OR Kirghizia OR Kirghiz OR Kyrgyzstan OR Lao PDR OR Laos OR Lebanon OR Lesotho OR Liberia OR Libya OR Macedonia OR Madagascar OR Malawi OR Malay OR Malaya OR Malaysia OR Maldives OR Mali OR Marshall Islands OR Mauritania OR Mauritius OR Mexico OR Micronesia OR Moldova OR Mongolia OR Montenegro OR Morocco OR Mozambique OR Myanmar OR Namibia OR Nepal OR Nicaragua OR Niger OR Nigeria OR Pakistan OR Palau OR Panama OR Papua New Guinea OR Paraguay OR Peru OR Philippines OR Principe OR Romania OR Ruanda OR Rwanda OR Samoa OR Sao Tome OR Senegal OR Serbia OR Sierra Leone OR Solomon Islands OR Somalia OR South Africa OR South Sudan OR Sri Lanka OR St Lucia OR St Vincent OR Sudan OR Surinam OR Suriname OR Swaziland OR Syria OR Syrian Arab Republic OR Tajikistan OR Tadzhikistan OR Tajikistan OR Tadzhik OR Tanzania OR Thailand OR Timor OR Togo OR Tonga OR Tunisia OR Turkey OR Turkmen OR Turkmenistan OR Tuvalu OR Uganda OR Ukraine OR Uzbek OR Uzbekistan OR Vanuatu OR Venezuela OR Vietnam OR West Bank OR Yemen OR Zambia OR Zimbabwe

BMJ Open: first published as 10.1136/bmjopen-2019-032912 on 3 February 2020. Downloaded from http://bmjopen.bmj.com/ on February 4, 2020 at University of Cape Town Libraries. Protected by copyright.

For peer review only - http://bmjopen.bmj.com/site/about/guidelines.xhtml

Table 2 Inclusion and exclusion criteria

Criteria for inclusion	Criteria for exclusion
► Published between January 1993 and November 2019.<br>► Qualitative and quantitative empirical (ie, primary research) study designs.<br>► Theoretical literature on the construct of recovery.<br>► Studies will be included if they have been conducted in LMICs.<br>► Peer-reviewed scientific literature.<br>► Literature focusing on the population of interest, that is, adults with SMI in LMICs will be included. The diagnosis of SMI includes schizophrenia spectrum disorders, schizoaffective disorders, bipolar affective disorders, major depressive disorder or related psychotic disorders (schizophreniform, delusional disorder, substance induced psychotic disorder or disorders not otherwise specified) as well as those with a comorbid substance use disorder. Studies and reports will be excluded if they do not include the keywords or alternatives as outlined in table 1.<br>► Explicit mention of severe mental illness and recovery in LMICS.	► The review will be limited to full-text articles written in English. However, the authors acknowledge that this practical decision could mean that potentially relevant papers are missed.<br>► Conference abstracts will be excluded although they will be reviewed to see if full reports were published.<br>► Children will be excluded as the age range for inclusion is 18 years and older.<br>► Duplicate articles from the same study will be excluded.<br>► Policy documents will be excluded although they will be reviewed to access research in LMICs that they might have referenced.<br>► Studies that occurred only in high-income countries, without an LMIC focus will be excluded.<br>► Studies must be conducted in LMIC and not in HIC with participants from LMICs.

LMICs, low-income and middle-income countries.

the inclusion and exclusion criteria. At both levels of screening, inter-rater agreement will be assessed by calculating Cohen's kappa. Any disagreement on study selection will be resolved by discussion with a third rater, who will be a senior member of the research team.

Quality appraisal

The Mixed Methods Appraisal Tool V.2018[48] and the Critical Appraisal Skills Programme Qualitative checklist will be used to assess the quality of included studies although this is not required for a scoping review nor will it influence the inclusion of the study.

Using the aforementioned criteria, the number of studies identified and selected for inclusion in the scoping review will be reported on. A narrative description[39] will accompany the search decision flow chart.[43] The flow chart will show the results from the search, the removal of duplicates, study selection, full retrieval numbers, additional searches from reference lists and a final summary of decisions related to the search decision and an explanation of the review decision process.[39]

Stage 4: charting the data

Next, the two reviewers (FG and KS) will independently extract data from the identified articles and chart it.[40] A draft data charting form will be created in the MS Excel programme to facilitate this process.[40] This form will be pretested and an iterative process of refining the form will be undertaken as the review commences to ensure that all relevant information is extracted.[39]

The form will allow the reviewers to confirm study relevance, record study characteristics and extract information relevant to the review question. The following information will be extracted from the articles: (1) study title; (2) author; (3) study population; (4) participant characteristics; (5) research question; (6) study methodology; (7) study description; (8) intervention type (if applicable); (9) intervention duration (if applicable); (10) outcome measures; 11 summary of findings; (12) definitions of key concepts (recovery and SMIs); (13) conceptual links identified; (14) practice implications and (15) recommendations for further development.

While charting the data, it is important to maintain flexibility around including emerging categories and consulting with the team as this occurs. The final version of the charting form along with definitions of included items will be provided as an appendix to the review. This process will facilitate the timeous completion of the review while also maintaining research rigour.

Stage 5: collating, summarising and reporting the results

All results will be collated, summarised and reported on in order to provide a comprehensive and thorough overview of all the reviewed literature. The authors plan to follow an iterative process and not pre-empt or fit findings into what has already been written about recovery in HICs. This will allow new understandings to be uncovered. While the CHIME framework is considered to offer a comprehensive overview of the process of recovery, it has only been used in HICs with predominantly White populations so its applicability with ethnically and culturally diverse groups is unknown.[12 30] The role of non-government organisations and traditional healers in providing services that facilitate recovery for people living with SMIs will be examined if this arises from the scoping review.

For peer review only - http://bmjopen.bmj.com/site/about/guidelines.xhtml

BMJ Open: first published as 10.1136/bmjopen-2019-032912 on 3 February 2020. Downloaded from http://bmjopen.bmj.com/ on February 4, 2020 at University of Cape Town Libraries. Protected by copyright.

Open access

A three-step approach[45] of analysing the data, reporting results and applying meaning to results will be followed to provide methodological rigour. Collating and summarising results will include a summary of the types of studies conducted (including the quantity, types of study design, populations, setting) as well as a thematic analysis of the results. More specifically, we will code how studies describe the concept of recovery from SMI and how it is defined and understood in LMICs. The framework used to report the findings will be developed iteratively through the examination of results. Additionally, the authors will report on the broader implications of the review in terms of research, policy and practice.

Stage 6: consultation

While Arksey and O'Malley[40] view the consultation stage as an optional step, Levac *et al*[45] views it as a requirement to ensure methodological rigour. During each stage of the review process, we will consult with an existing stakeholder advisory committee. This advisory committee consists of the research team and key stakeholders including mental health service providers and service users from within the public health and non-profit organisation sectors. This committee has been constituted from the onset of the project and some members are part of a pre-existing group for public mental health specialists. The advisory committee will be consulted to get their views on the relevance of the review, guidance for accessing grey literature and their perspectives on the data extraction process and preliminary findings. Including an advisory committee in the review is informed by a pragmatic approach[49] where the research is treated as a human experience with the understanding that this consultation process is likely to enhance the relevance of this review. It will also support collaborative efforts to facilitate consumer participation, public involvement, access to information and transfer of knowledge.[45]

Patient and public involvement

There is no patient involvement in the protocol development aspect of the study. Service users forming part of the advisory committee referred to stage 6 will be consulted on findings and dissemination of results.

Ethics and dissemination

Since the scoping review methodology aims at synthesising information from available publications, this study does not require ethical approval. Reviews of primary research allow for the mapping of evidence in fields where the body of literature has not yet been comprehensively reviewed or where working definitions and concepts are still developing. The findings will be made available in different formats to facilitate its dissemination. An article reporting on the results will be submitted for publication in a peer-reviewed journal. It will also be presented at relevant conferences and stakeholder engagements involved in mental health policy and practice. These stakeholders include mental health service users (MHSUs), academics, policy-makers, researcher and clinicians. Findings will also be shared via newsletters, policy briefs and social media forums.

Twitter Fadia Gamieldien @fadiagam

Contributors This protocol was developed by the intellectual contributions of all the authors. All authors were involved in developing the review question and conceptualising the approach. FG developed and tested the search terms in consultation with a subject librarian. FG and KS developed the data extraction guideline, and this was revised based on feedback from the other authors. RG, KS and BM provided substantial critique and review of the draft protocol.

Funding This work was supported by the DELTAS Africa Initiative (DEL-15-01). The DELTAS Africa Initiative is an independent funding scheme of the African Academy of Sciences (AAS)'s Alliance for Accelerating Excellence in Science in Africa (AESA) and supported by the New Partnership for Africa's Development Planning and Coordinating Agency (NEPAD Agency) with funding from the Wellcome Trust (DEL-15-01) and the UK government. BM is funded through the South African Medical Research Council.

Disclaimer The views expressed in this publication are those of the author(s) and not necessarily those of AAS, NEPAD Agency, WellcomeTrust or the UK government.

Competing interests None declared.

Patient consent for publication Not required.

Provenance and peer review Not commissioned; externally peer reviewed.

Open access This is an open access article distributed in accordance with the Creative Commons Attribution 4.0 Unported (CC BY 4.0) license, which permits others to copy, redistribute, remix, transform and build upon this work for any purpose, provided the original work is properly cited, a link to the licence is given, and indication of whether changes were made. See: https://creativecommons.org/licenses/by/4.0/.

ORCID iD
Fadia Gamieldien http://orcid.org/0000-0003-2820-6484

REFERENCES

1 Whiteford HA, Ferrari AJ, Degenhardt L, *et al*. The global burden of mental, neurological and substance use disorders: an analysis from the global burden of disease study 2010. *PLoS One* 2015;10:e0116820.
2 Vigo D, Thornicroft G, Atun R. Estimating the true global burden of mental illness. *Lancet Psychiatry* 2016;3:171–8.
3 Kessler RC, Berglund PA, Bruce ML, *et al*. The prevalence and correlates of untreated serious mental illness. *Health Serv Res* 2001;36:987.
4 Petersen I, Lund C, Stein DJ. Optimizing mental health services in low-income and middle-income countries. *Curr Opin Psychiatry* 2011;24:318–23.
5 Sfetcu R, Musat S, Haaramo P, *et al*. Overview of post-discharge predictors for psychiatric re-hospitalisations: a systematic review of the literature. *BMC Psychiatry* 2017;17.
6 Kohn R, Saxena S, Levav I, *et al*. The treatment gap in mental health care. *Bull World Health Organ* 2004;82:858–66.
7 Demyttenaere K, Bruffaerts R, Posada-Villa J, *et al*. Prevalence, severity, and unmet need for treatment of mental disorders in the World Health Organization world mental health surveys. *JAMA* 2004;291:2581–90.
8 Eaton J, McCay L, Semrau M, *et al*. Scale up of services for mental health in low-income and middle-income countries. *Lancet* 2011;378:1592–603.
9 Thornicroft G, Cooper S, Bortel TV, *et al*. Capacity building in global mental health research. *Harv Rev Psychiatry* 2012;20:13–24.
10 Kumar A. Mental health services in rural India: challenges and prospects. *Health* 2011;03:757–61.
11 Lund C, Oosthuizen P, Flisher AJ, *et al*. Pathways to inpatient mental health care among people with schizophrenia spectrum disorders in South Africa. *PS* 2010;61:235–40.
12 Leamy M, Bird V, Boutillier CL, *et al*. Conceptual framework for personal recovery in mental health: systematic review and narrative synthesis. *Br J Psychiatry* 2011;199:445–52.
13 Slade M. *Personal recovery and mental illness: a guide for mental health professionals*. Cambridge University Press, 2009.

BMJ Open: first published as 10.1136/bmjopen-2019-032912 on 3 February 2020. Downloaded from http://bmjopen.bmj.com/ on February 4, 2020 at University of Cape Town Libraries. Protected by copyright.

For peer review only - http://bmjopen.bmj.com/site/about/guidelines.xhtml

14 Slade M. Mental illness and well-being: the central importance of positive psychology and recovery approaches. *BMC Health Serv Res* 2010;10:26.
15 Myers NL. Culture, stress and recovery from schizophrenia: lessons from the field for global mental health. *Cult Med Psychiatry* 2010;34:500–28.
16 Tew J, Ramon S, Slade M, *et al*. Social factors and recovery from mental health difficulties: a review of the evidence. *Br J Soc Work* 2012;42:443–60.
17 Anthony WA. Recovery from mental illness: the guiding vision of the mental health service system in the 1990s. *Psychiatr Rehabil J* 1993;16:11–23.
18 Deegan PE. Recovery: the lived experience of rehabilitation. *Psychiatr Rehabil J* 1988;11:11–19.
19 Webb L. The recovery model and complex health needs: what health psychology can learn from mental health and substance misuse service provision. *J Health Psychol* 2012;17:731–41.
20 Farkas M. The vision of recovery today: what it is and what it means for services. *World Psychiatry* 2007;6:68.
21 Mathew ST, Nirmala BP, Kommu JVS. Meaning of recovery among persons with schizophrenia: a literature review. *J Psychosoc Rehabil Mental Health* 2018;5:73–81.
22 Kelly M, Lamont S, Brunero S. An occupational perspective of the recovery journey in mental health. *Br J Occup Ther* 2010;73:129–35.
23 Cabassa LJ, Nicasio A, Whitley R. Picturing recovery: a photovoice exploration of recovery dimensions among people with serious mental illness. *Psychiatr Serv* 2013;64:837–42.
24 Aldersey HM, Adeponle AB, Whitley R. Diverse approaches to recovery from severe mental illness. The Palgrave Handbook of sociocultural perspectives on global mental health 2017:109–27.
25 Frost BG, Tirupati S, Johnston S, *et al*. An integrated Recovery-oriented model (IRM) for mental health services: evolution and challenges. *BMC Psychiatry* 2017;17:22.
26 Lysaker PH, Hamm JA, Hasson-Ohayon I, *et al*. Promoting recovery from severe mental illness: implications from research on metacognition and metacognitive reflection and insight therapy. *World J Psychiatry* 2018;8:1–11.
27 Cohen A, Patel V, Thara R, *et al*. Questioning an axiom: better prognosis for schizophrenia in the developing world? *Schizophr Bull* 2008;34:229–44.
28 Patel V, Saxena S, Lund C, *et al*. The Lancet Commission on global mental health and sustainable development. *Lancet* 2018;392:1553–98.
29 van Weeghel J, van Zelst C, Boertien D, *et al*. Conceptualizations, assessments, and implications of personal recovery in mental illness: a scoping review of systematic reviews and meta-analyses. *Psychiatr Rehabil J* 2019;42:169–81.
30 Brijnath B. Applying the CHIME recovery framework in two culturally diverse Australian communities: qualitative results. *Int J Soc Psychiatry* 2015;61:660–7.
31 Leonhardt BL, Huling K, Hamm JA, *et al*. Recovery and serious mental illness: a review of current clinical and research paradigms and future directions. *Expert Rev Neurother* 2017;17:1117–30.
32 Kirst M, Zerger S, Wise Harris D, *et al*. The promise of recovery: narratives of hope among homeless individuals with mental illness participating in a housing first randomised controlled trial in Toronto, Canada: Table 1. *BMJ Open* 2014;4:e004379.
33 Smartt C, Prince M, Frissa S, *et al*. Homelessness and severe mental illness in low- and middle-income countries: scoping review. *BJPsych Open* 2019;5.
34 Schön U-K, Denhov A, Topor A. Social relationships as a decisive factor in recovering from severe mental illness. *Int J Soc Psychiatry* 2009;55:336–47.
35 Llewellyn-Beardsley J, Rennick-Egglestone S, Callard F, *et al*. Characteristics of mental health recovery narratives: systematic review and narrative synthesis. *PLoS One* 2019;14:e0214678.
36 Kpanake L. Cultural concepts of the person and mental health in Africa. *Transcult Psychiatry* 2018;55:198–218.
37 Janse van Rensburg ABR. South African Society of Psychiatrists guidelines for the integration of spirituality in the approach to psychiatric practice. *S Afr J Psychiatr* 2014;20:7–39.
38 Swartz L, Foster D. Images of culture and mental illness : South African psychiatric approaches. *Soc Dyn* 1984;10:17–25.
39 Peters MDJ, Godfrey CM, Khalil H, *et al*. Guidance for conducting systematic scoping reviews. *Int J Evid Based Healthc* 2015;13:141–6.
40 Arksey H, O'Malley L. Scoping studies: towards a methodological framework. *Int J Soc Res Methodol* 2005;8:19–32.
41 Dijkers M. What is a scoping review?. KT Update 2015;4.
42 McKinstry C, Brown T, Gustafsson L. Scoping reviews in occupational therapy: the what, why, and how to. *Aust Occup Ther J* 2014;61:58–66.
43 Tricco AC, Lillie E, Zarin W, *et al*. Prisma extension for scoping reviews (prisma-scr): checklist and explanation. *Ann Intern Med* 2018;169:467–73.
44 Pham MT, Rajić A, Greig JD, *et al*. A scoping review of scoping reviews: advancing the approach and enhancing the consistency. *Res Synth Methods* 2014;5:371–85.
45 Levac D, Colquhoun H, O'Brien KK. Scoping studies: advancing the methodology. *Implement Sci* 2010;5:69.
46 Slade M, Longden E. Empirical evidence about recovery and mental health. *BMC Psychiatry* 2015;15:285.
47 Shanks V, Williams J, Leamy M, *et al*. Measures of personal recovery: a systematic review. *Psychiatr Serv* 2013;64:974–80.
48 Hong QN, Pluye P, Fàbregues S, *et al*. Mixed methods appraisal tool (MMAT), version 2018. *IC Canadian Intellectual Property Office, Industry Canada* 2018.
49 Morgan DL. Pragmatism as a paradigm for social research. *Qual Inq* 2014;20:1045–53.

For peer review only

BMJ Open: first published as 10.1136/bmjopen-2019-032912 on 3 February 2020. Downloaded from http://bmjopen.bmj.com/ on February 4, 2020 at University of Cape Town Libraries. Protected by copyright.

For peer review only - http://bmjopen.bmj.com/site/about/guidelines.xhtml

Supplementary file 2

PubMed Search

Population, Concept, Context (PCC)

**Population:**		
#1	MeSH terms:	Schizophrenia Spectrum and Other Psychotic Disorders<br>Bipolar and Related Disorders<br>Depressive disorder, Major
#2	Free text:	Severe mental illness OR bipolar OR delusional disorder OR delusional disorders OR major depressive disorder OR major depressive disorders OR schizophrenia OR manic OR manic-depressive OR paranoid disorder OR paranoid disorders OR psychoses OR psychosis OR psychotic disorder OR psychotic disorders OR schizoaffective disorder OR schizoaffective disorders OR Schizophreniform OR serious mental disorder OR serious mental disorders
#3	#1 OR #2	
**Concept:**		
#4	MeSH terms:	Psychiatric Rehabilitation<br>Mental Health Recovery<br>Recovery of Function
#5	Free text:	Recovery OR recover OR psychosocial rehabilitation OR mental health rehabilitation OR psychiatric rehabilitation
#6	#4 OR #5	
**Context: Low and Middle Income Countries (LMICS)**		
#7	Free text:	Deprived Countries OR Deprived Population OR Deprived Populations OR Developing Countries OR Developing Country OR Developing Economies OR Developing Economy OR Developing Nation OR Developing Nations OR Developing Population OR Developing Populations OR Developing World OR LAMI Countries OR LAMI Country OR Less Developed Countries OR Less Developed Country OR Less Developed Economies OR Less Developed Nation OR Less Developed Nations OR Less Developed World OR Lesser Developed Countries OR Lesser Developed Nations OR LMIC OR LMICS OR Low GDP OR Low GNP OR Low Gross Domestic OR Low Gross National OR Low Income Countries OR Low Income Country OR Low Income Economies OR Low Income Economy OR Low Income Nations OR Low Income Population OR Low Income Populations OR Lower GDP OR lower gross domestic OR Lower Income Countries OR Lower Income Country OR Lower Income Nations OR Lower Income Population OR Lower Income Populations OR Middle Income Countries OR Middle Income Country OR Middle Income Economies OR Middle Income Nation OR Middle Income Nations OR Middle Income Population OR Middle Income Populations OR Poor Countries OR Poor Country OR Poor Economies OR Poor Economy OR Poor Nation OR Poor Nations OR Poor Population OR Poor Populations OR poor world OR Poorer Countries OR Poorer Economies OR Poorer Economy OR Poorer Nations OR Poorer Population OR Poorer Populations OR Third World OR Transitional Countries OR Transitional Country OR Transitional Economies OR Transitional Economy OR Under Developed Countries OR Under Developed Country OR under developed nations OR Under Developed World OR Under Served Population OR Under Served Populations OR Underdeveloped Countries OR Underdeveloped Country OR

For peer review only

		underdeveloped economies OR underdeveloped nations OR underdeveloped population OR Underdeveloped World OR Underserved Countries OR Underserved Nations OR Underserved Population OR Underserved Populations
#8	Free text:	Afghanistan OR Albania OR Algeria OR American Samoa OR Angola OR Armenia OR Azerbaijan OR Bangladesh OR Belarus OR Byelarus OR Belorussia OR Belize OR Benin OR Bhutan OR Bolivia OR Bosnia OR Botswana OR Brazil OR Bulgaria OR Burma OR Burkina Faso OR Burundi OR Cabo Verde OR Cape Verde OR Cambodia OR Cameroon OR Central African Republic OR Chad OR China OR Colombia OR Comoros OR Comores OR Comoro OR Congo OR Costa Rica OR Côte d'Ivoire OR Cuba OR Djibouti OR Dominica OR Dominican Republic OR Ecuador OR Egypt OR El Salvador OR Equatorial Guinea OR Eritrea OR Ethiopia OR Fiji OR Gabon OR Gambia OR Gaza OR Georgia OR Georgia Republic OR Ghana OR Grenada OR Grenadines OR Guatemala OR Guinea OR Guinea- Bissau OR Guyana OR Haiti OR Herzegovina OR Hercegovina OR Honduras OR India OR Indonesia OR Iran OR Iraq OR Ivory Coast OR Jamaica OR Jordan OR Kazakhstan OR Kenya OR Kiribati OR Democratic People's Republic of Korea OR Kosovo OR Kyrgyz OR Kirghizia OR Kirghiz OR Kyrgyzstan OR Lao PDR OR Laos OR Lebanon OR Lesotho OR Liberia OR Libya OR Macedonia OR Madagascar OR Malawi OR Malay OR Malaya OR Malaysia OR Maldives OR Mali OR Marshall Islands OR Mauritania OR Mauritius OR Mexico OR Micronesia OR Moldova OR Mongolia OR Montenegro OR Morocco OR Mozambique OR Myanmar OR Namibia OR Nepal OR Nicaragua OR Niger OR Nigeria OR Pakistan OR Palau OR Papua New Guinea OR Paraguay OR Peru OR Philippines OR Principe OR Romania OR Ruanda OR Rwanda OR Samoa OR Sao Tome OR Senegal OR Serbia OR Sierra Leone OR Solomon Islands OR Somalia OR South Africa OR South Sudan OR Sri Lanka OR St Lucia OR St Vincent OR Sudan OR Surinam OR Suriname OR Swaziland OR Syria OR Syrian Arab Republic OR Tajikistan OR Tadzhikistan OR Tajikistan OR Tadzhik OR Tanzania OR Thailand OR Timor OR Togo OR Tonga OR Tunisia OR Turkey OR Turkmen OR Turkmenistan OR Tuvalu OR Uganda OR Ukraine OR Uzbek OR Uzbekistan OR Vanuatu OR Venezuela OR Vietnam OR West Bank OR Yemen OR Zambia OR Zimbabwe
#9	#7 OR #8	
#10	#3 AND #6 AND #9	887 Results (290519)
Filter	Only language filter used for PubMed as a preliminary search showed that all recent articles not yet indexed were excluded when filtered for NOT animals and year range	

For peer review only

Preferred Reporting Items for Systematic reviews and Meta-Analyses extension for Scoping Reviews (PRISMA-ScR) Checklist

SECTION	ITEM	PRISMA-ScR CHECKLIST ITEM	REPORTED ON PAGE #
**TITLE**			
Title	1	Identify the report as a scoping review.	1
**ABSTRACT**			
Structured summary	2	Provide a structured summary that includes (as applicable): background, objectives, eligibility criteria, sources of evidence, charting methods, results, and conclusions that relate to the review questions and objectives.	2
**INTRODUCTION**			
Rationale	3	Describe the rationale for the review in the context of what is already known. Explain why the review questions/objectives lend themselves to a scoping review approach.	5
Objectives	4	Provide an explicit statement of the questions and objectives being addressed with reference to their key elements (e.g., population or participants, concepts, and context) or other relevant key elements used to conceptualize the review questions and/or objectives.	1,5
**METHODS**			
Protocol and registration	5	Indicate whether a review protocol exists; state if and where it can be accessed (e.g., a Web address); and if available, provide registration information, including the registration number.	3,5 JBI registration, supplementary file 1
Eligibility criteria	6	Specify characteristics of the sources of evidence used as eligibility criteria (e.g., years considered, language, and publication status), and provide a rationale.	1,5, 6
Information sources*	7	Describe all information sources in the search (e.g., databases with dates of coverage and contact with authors to identify additional sources), as well as the date the most recent search was executed.	5,6, Protocol (supplementary file 1)
Search	8	Present the full electronic search strategy for at least 1 database, including any limits used, such that it could be repeated.	6, (supplementary file 2)
Selection of sources of evidence†	9	State the process for selecting sources of evidence (i.e., screening and eligibility) included in the scoping review.	6-9
Data charting process‡	10	Describe the methods of charting data from the included sources of evidence (e.g., calibrated forms or forms that have been tested by the team before their use, and whether data charting was done independently or in duplicate) and any processes for obtaining and confirming data from investigators.	7
Data items	11	List and define all variables for which data were sought and any assumptions and simplifications made.	7

For peer review only

SECTION	ITEM	PRISMA-ScR CHECKLIST ITEM	REPORTED ON PAGE #
Critical appraisal of individual sources of evidence§	12	If done, provide a rationale for conducting a critical appraisal of included sources of evidence; describe the methods used and how this information was used in any data synthesis (if appropriate).	7
Synthesis of results	13	Describe the methods of handling and summarizing the data that were charted.	7
**RESULTS**			
Selection of sources of evidence	14	Give numbers of sources of evidence screened, assessed for eligibility, and included in the review, with reasons for exclusions at each stage, ideally using a flow diagram.	8
Characteristics of sources of evidence	15	For each source of evidence, present characteristics for which data were charted and provide the citations.	8
Critical appraisal within sources of evidence	16	If done, present data on critical appraisal of included sources of evidence (see item 12).	7
Results of individual sources of evidence	17	For each included source of evidence, present the relevant data that were charted that relate to the review questions and objectives.	8-11
Synthesis of results	18	Summarize and/or present the charting results as they relate to the review questions and objectives.	12-20
**DISCUSSION**			
Summary of evidence	19	Summarize the main results (including an overview of concepts, themes, and types of evidence available), link to the review questions and objectives, and consider the relevance to key groups.	15-22
Limitations	20	Discuss the limitations of the scoping review process.	3, 22
Conclusions	21	Provide a general interpretation of the results with respect to the review questions and objectives, as well as potential implications and/or next steps.	22
**FUNDING**			
Funding	22	Describe sources of funding for the included sources of evidence, as well as sources of funding for the scoping review. Describe the role of the funders of the scoping review.	23

JBI = Joanna Briggs Institute; PRISMA-ScR = Preferred Reporting Items for Systematic reviews and Meta-Analyses extension for Scoping Reviews.

* Where *sources of evidence* (see second footnote) are compiled from, such as bibliographic databases, social media platforms, and Web sites.

† A more inclusive/heterogeneous term used to account for the different types of evidence or data sources (e.g., quantitative and/or qualitative research, expert opinion, and policy documents) that may be eligible in a scoping review as opposed to only studies. This is not to be confused with *information sources* (see first footnote).

‡ The frameworks by Arksey and O'Malley (6) and Levac and colleagues (7) and the JBI guidance (4, 5) refer to the process of data extraction in a scoping review as data charting.

§ The process of systematically examining research evidence to assess its validity, results, and relevance before using it to inform a decision. This term is used for items 12 and 19 instead of "risk of bias" (which is more applicable to systematic reviews of interventions) to include and acknowledge the various sources of evidence that may be used in a scoping review (e.g., quantitative and/or qualitative research, expert opinion, and policy document).

For peer review only - http://bmjopen.bmj.com/site/about/guidelines.xhtml

From: Tricco AC, Lillie E, Zarin W, O'Brien KK, Colquhoun H, Levac D, et al. PRISMA Extension for Scoping Reviews (PRISMAScR): Checklist and Explanation. Ann Intern Med. 2018;169:467–473. doi: 10.7326/M18-0850.

For peer review only
